# Engineering of donor-acceptor-donor curcumin analogues as near-infrared fluorescent probes for *in vivo* imaging of amyloid-β species

**DOI:** 10.7150/thno.68679

**Published:** 2022-04-04

**Authors:** Daqing Fang, Xidan Wen, Yuqi Wang, Yidan Sun, Ruibing An, Yu Zhou, Deju Ye, Hong Liu

**Affiliations:** 1 Key Laboratory of Structure-Based Drug Design and Discovery, Ministry of Education, Shenyang Pharmaceutical University, Shenyang, 110016, China.; 2 State Key Laboratory of Analytical Chemistry for Life Sciences, Chemistry and Biomedicine Innovation Center (ChemBIC), School of Chemistry and Chemical Engineering, Nanjing University, Nanjing, 210023, China.; 3 State Key Laboratory of Drug Research, Shanghai Institute of Materia Medica, Chinese Academy of Sciences, Shanghai, 201203, China.

**Keywords:** Alzheimer's disease, Amyloid-β, NIR fluorescence probe, *in vivo* imaging, D-A-D molecules

## Abstract

Near-infrared (NIR) fluorescent imaging of both soluble and insoluble Aβ species in the brain of Alzheimer's disease (AD) is crucial for the early diagnosis and intervention of AD. To date, a variety of NIR fluorescent probes have been reported for the detection of Aβ species. Among these probes, CRANAD-58 was reported to have the capability to detect both soluble and insoluble Aβ species, which is vital to monitor the changes of Aβ species during the pathological course of the disease. Though CRANAD-58 has shown promise to noninvasively detect Aβ species in transgenic AD mice, the emission wavelength (~670 nm) is still too short for further applications. Therefore, new probes with longer emission wavelength and improved physiological properties are in highly demand. Herein, we report the design and engineering of nine donor-acceptor-donor molecules as “off-on” near-infrared fluorescent probes for *in vivo* imaging of both soluble and insoluble Aβ species in living AD mice owing to its improved *in vitro* properties and *in vivo* performance.

**Methods:** We report a two-round strategy to develop nine “off-on” NIR fluorescence probes via structural modification of a curcumin analogue-based donor-acceptor-donor architecture. In round one, probes **1** and **2** were synthesized, and probe** 2** was identified to be an optimum probe as it showed distinct “off-on” NIR fluorescence at > 690 nm upon binding to Aβ monomers, oligomers and aggregates. To further improve the* in vivo* performance, further structural modification of probe **2** into probes **3**-**9** was then conducted. The fluorescence response with Aβ species and histological staining *in vitro* and *in vivo* imaging of Aβ species in APP/PS1 transgenic AD mice and age-matched wild-type mice were performed.

**Results:** We demonstrate that, compared to probe** 2**, probe **9** with improved physiological properties hold the fastest kinetics (~10 min) to produce not only higher brain fluorescence intensity in 10-month-old APP/PS1 transgenic AD mice, but also afford a higher discrepancy in brain fluorescence to discriminate AD mice from wild-type (WT) mice. Probe **9** also hold the ability to detect soluble Aβ species in 6-month-old APP/PS1 transgenic mice. Probe **9** was further applied for dynamic visualization of Aβ plaques in a skull-thinning 14-month-old APP/PS1 mouse, which revealed its immediate penetration into brain parenchyma and selective labeling of both parenchymal and angiopathic Aβ plaques. In addition, probe** 9** possessed significantly high attenuation effect on the aggregation of Aβ monomers.

**Conclusion:** Our results demonstrate the good potential of probe **9** for longitudinal NIR fluorescence imaging of soluble and insoluble Aβ species in APP/PS1 transgenic AD mice, which may act as a useful tool for early diagnosis and intervention of AD.

## Introduction

Alzheimer's disease (AD) is one of the most notorious neurodegenerative diseases, which progressively causes cognitive decline, irreversible memory loss, and disorientation. AD occurs with several pathological hallmarks, such as aggregation of amyloid-β (Aβ) peptides into fibrils and plaques, formation of tau protein tangles, and upregulation of reactive oxygen species (ROS) 1-3. It has been recognized that Aβ species, including soluble monomers, dimers, oligomers, and insoluble fibrils/aggregates and plaques, can exert high toxicity against neuronal cells 4, 5. During the course of AD progression, all types of Aβ species are present, with the predominance of the subspecies progressively changing from soluble species to insoluble fibrils and plaques 6. Methods capable of detecting both soluble and insoluble Aβ species can provide the potential to monitor the progression of AD, which are desirable for early definitive diagnosis of AD.

Over the past decades, tremendous efforts have been devoted to detect Aβ species, and molecular imaging techniques, such as magnetic resonance imaging (MRI) 7, single-photon emission-computed tomography (SPECT) 8, positron emission computed tomography (PET) 9, and optical imaging 10 have shown promise due to the advantages of noninvasiveness, and real-time manner, allowing to monitor the progression of AD at molecular and cellular levels. Till now, a few PET tracers, such as ^18^F-labeled florbetapir, flutemetamol and florbetaben, have been approved for the detection of Aβ plaques in clinics 11-14, while the use of radioactive isotopes requires expensive cyclotron and well skilled chemists, which will place high cost to the patients; additionally, the unavoidable exposure to radiation from administrated radiotracers has also impeded their routine applications. Alternately, people have developed a number of fluorescent probes, such as Congo Red (CR), Thioflavin T (ThT) and Pittsburgh B (PIB), for the detection of Aβ species as fluorescence imaging possesses much lower cost, easier operation and avoidance of radiation exposure compared to PET imaging 15. Particularly, many fluorescent probes with their emission in the near infrared (NIR) region (λ > 650 nm) capable of improving tissue penetration depth and reducing autofluorescence of biological tissues, have emerged as promising tools for *in vivo* imaging of Aβ deposits and helping diagnosis of AD 16-34. Most NIR fluorescent probes for Aβ species have been designed to have an electron donor (D)-acceptor (A) or D-A-D architecture, which showed weak NIR fluorescence in aqueous solution, but strong fluorescence upon binding to Aβ species 10. For example, Swager reported NIAD-4 with a D-A architecture as the first NIR fluorescent probe for *in vivo* imaging of Aβ plaques 35, however, the relatively short absorption and emission wavelength (λ_ex/em_ = 475/612 nm) could allow it to work only in cranial window-implanted transgenic AD mice under two-photon excitation. Saji and coworkers lately employed a boron dipyrromethane (BODIPY) fluorophore as the acceptor and a dimethylamino styryl group as the donor, and reported a new D-A type of NIR fluorescent probe (BAP-1) 36, which showed longer absorption and emission wavelengths (λ_ex/em_ = 604/648 nm) than that of NIAD-4 35; however, the nonspecific distribution in the scalp largely lowered the *in vivo* imaging ability. Cui and coworkers reported another series of D-A molecules (DANIRs) by linking the electron-donating *N*,*N′*-dimethylamino group (D) and electron-accepting malononitrile group (A) through varying lengths of π-conjugated bonds 22-27, 32, 37; probe (3c) 24 showed a remarkably enhanced NIR fluorescence (λ_em_ = 678 nm) upon binding with Aβ aggregates and could efficiently penetrate the blood brain barrier (BBB) to differentiate transgenic AD mice from wild-type mice through noninvasive fluorescence imaging. Ran *et al.* 17-21, 38 reported curcumin analogues with a D-A-D architecture as prominent NIR fluorescent probes, and among them, CRANAD-58 displayed high affinities not only to the insoluble Aβ aggregates, but also to soluble Aβ monomers and oligomers, offering enhanced NIR fluorescence emission (λ_ex/em_ ≈ 580/670 nm) to noninvasively detect Aβ species in transgenic AD mice 18. Despite encouraging progresses made, there is still challenging to design high performance NIR fluorescence probes capable of fast crossing BBB and noninvasively detecting Aβ species, particularly soluble Aβ monomers and oligomers with high sensitivity and specificity. Such probes could be amenable for the detection of AD at an early stage, grateful for early intervention of AD prior to the appearance of obvious symptoms.

**Figure 1:**
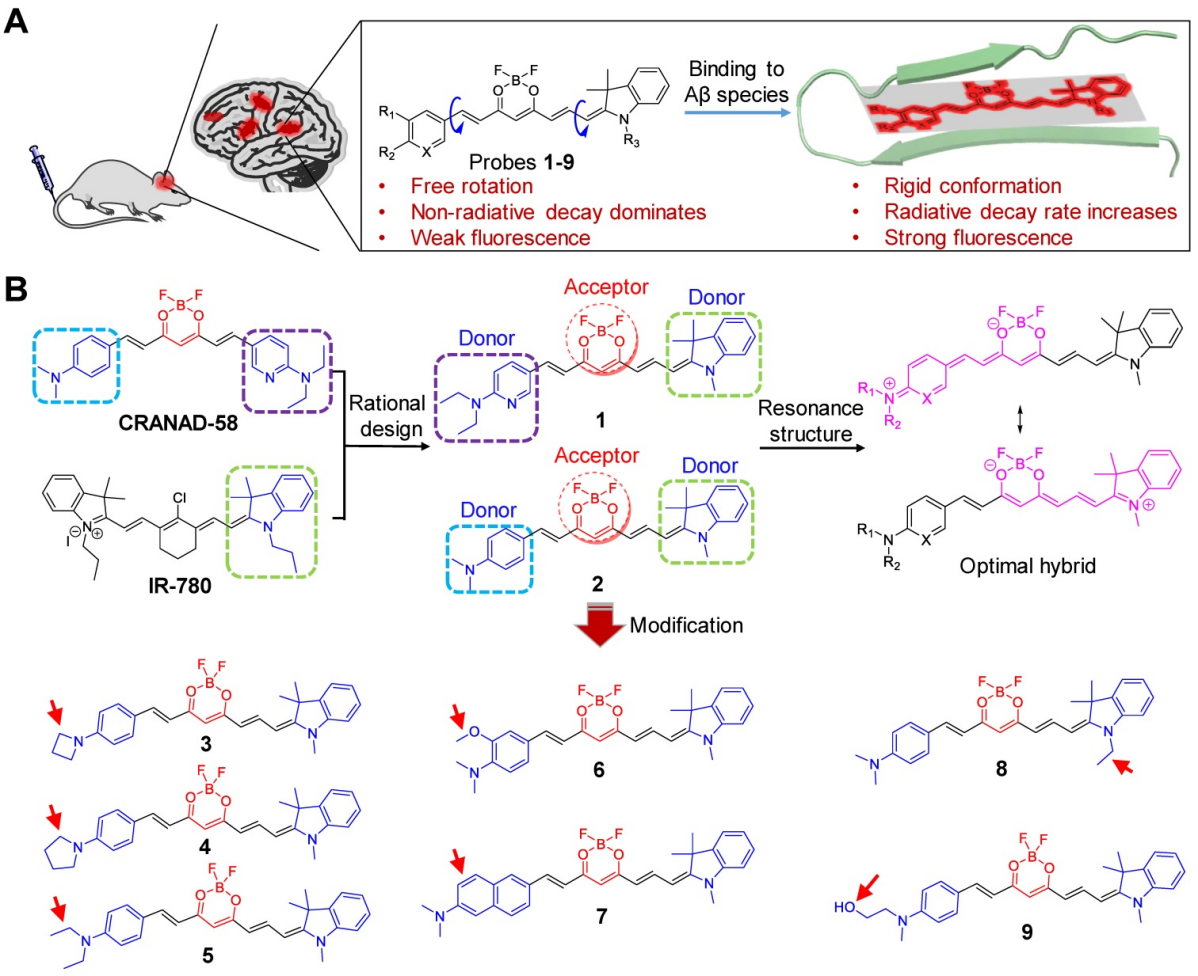
** (A)** The proposed mechanism for *in vivo* imaging of Aβ species via binding-induced fluorescence “turn on”. **(B)** Design of NIR fluorescent probes **1** and **2** by hybridizing CRANAD-58 and NIR-780, and chemical modification of probe **2** into probes** 3**-**9**.

Herein, we report a two-round strategy to develop sensitive “off-on” NIR fluorescence probes by modifying CRANAD-58 with a D-A-D chemical architecture. In round one, we designed probes **1** and **2**, and identified probe **2** was an optimum probe, which showed improved fluorescence quantum yield (*Φ*_f_) and red-shifted absorption and fluorescence emission over CRANAD-58 upon binding to either insoluble Aβ aggregates or soluble Aβ monomers and oligomers. Based on probe **2**, the second round of modification allowed us to design probes **3**-**9** by improving binding affinity, augmenting fluorescence turn-on ratio and balancing lipophilicity to improve brain uptake. Following systemic administration, we demonstrated that probes **2**,** 4**, **6** and **9** with good *in vitro* performance could rapidly cross BBB and produce significantly brighter NIR fluorescence in the brains of APP/PS1 transgenic AD mice compared to the age-matched WT mice. We further employed an upright microscope to dynamically visualize the entry of probe **9** into brain parenchyma and selective labeling of both parenchymal and angiopathic Aβ plaques in a skull-thinning 14-month-old APP/PS1 mouse, not in WT mice. Additionally, as shown in Figure 1A, the mechanism of designed probes for fluorescence imaging of Aβ species in living mice of AD model was proposed. Following intravenous administration, the probes can cross the BBB and enter into brain. In the absence of Aβ species, these probes with the D-A-D architecture possess a substantial degree of conformational freedom and a non-radiative decay process dominates, thus exhibiting weak fluorescence. However, in the brain with Aβ species existence, the probes can bind to the Aβ species and the conformation is restricted, which substantially decreases the vibrational-rotational processes, leading to an increase in the radiative decay rate. Thus, enhanced NIR fluorescence appeared, which can provide sensitive signals to noninvasively visualize the Aβ species in the brains of living AD mice.

## Materials and Methods

### Materials

All chemical reagents were reagent grade and used as purchased from commercial sources (such as Aldrich, Adamas) without further purification. Aβ (1-42) monomer was purchased from Nanjing Peptide Biotech Ltd. (Nanjing, China). High glucose Dulbecco's Modified Eagle's Medium (DMEM), fetal bovine serum (FBS), penicillin/streptomycin were purchased from Thermo (Shanghai, China). 3-(4,5-Dimethylthiazol-2-yl)-2,5-diphenyltetrazolium bromide (MTT) kit was obtained from KeyGen Biotech. Co. Ltd. (Nanjing, China). Normal ICR mice (5 weeks, male), BALB/c mice (5 weeks, female), Transgenic mice (C57BL6, APPswe/PSEN1, 4-14 months old, male), and its littermates were purchased from the Model Animal Research Center (MARC) of Nanjing University (Nanjing, China). All animal experiments were approved by the Institutional Animal Care and Use Committee (IACUC) of Nanjing University.

### Preparation of Aβ42 monomers, oligomers and aggregates

Aβ monomers were prepared by dissolving commercial Aβ_42_ peptide in hexafluoroisopropanol at a concentration of 250 μΜ as a stock.

Aβ_42_ oligomers was prepared according to the procedure reported previously 39, and confirmed by TEM. Briefly, Soluble oligomers were prepared by dissolving 1.0 mg Aβ in 400 μL hexafluoroisopropanol (HFIP) at room temperature and stayed for 15 min. 100 μL of the resulting Aβ solution was added to 900 μL D.I. H_2_O in a siliconized Eppendorf tube. After incubation at room temperature for another 15 min, the samples were centrifuged at 14,000 × G for 15 min, and the supernatant was transferred to a new siliconized tube and subjected to a gentle stream of N_2_ for 10 min to evaporate the HFIP. The samples were then stirred at 500 rpm using a Teflon coated micro stir bar at ~22 °C for 24-48 h.

To prepare Aβ_42_ aggregates, the Aβ_42_ peptide (1.0 mg) was suspended in 1% ammonia hydroxyl solution (1.0 mL). One hundred microliters of the resulting solution were diluted 10-fold with PBS buffer (pH = 7.4), and kept stirring at room temperature for 3 days. TEM analysis was applied to confirm the formation of Aβ aggregates.

### Response of probes toward Aβ species

To test interaction of each probe with Aβ species, the following procedure was utilized. (1) Each probe was added to 2.0 mL PBS buffer (pH = 7.4) (250 nM of final concentration); (2) Aβ species (25 μΜ stock solution in HFIP for monomers, and 25 μΜ stock solution in PBS buffer or D.I. water for oligomers and aggregates) were added to the probe solution to make the final Aβ species concentration of 250 nM; (3) the mixture was transferred to quartz cuvette and its fluorescence spectra were recorded by HORIBA Jobin Yvon Fluoromax-4 fluorometer; (4) the fluorescence spectra of PBS buffer or each probe in PBS buffer alone was also measured using the same parameters in (3). The fold increase of the fluorescence intensity after binding to the Aβ species was calculated by the following equation:

Fold enhancement = (FI_test_-FI_PBS_)/(FI_probe_-FI_PBS_)

Where FI_test_, FI_probe_ and FI_PBS_ represent the fluorescence intensities of probes upon binding to Aβ species, the solution of probes in PBS, and PBS alone, respectively.

### TEM Measurement

Ten microliters of 250 nM of Aβ_42_ oligomers or aggregates in PBS solution were pipetted onto a carbon-coated copper grid, followed by the addition of 10 μL of a PTA staining solution to the grid. After 1 min, the liquid on the grid was carefully dried with a corner of filter paper, and the resulting grid was further dried in the air for 10 min. The TEM images were obtained with the JEM-1011 TEM.

### Confocal fluorescence imaging of Aβ oligomers or Aβ aggregates

To a PBS buffer (pH = 7.4) solution of Aβ oligomers or Aβ aggregates (25 μM), probe **2** was added to be a final concentration of 2.5 μM. After being mixed, the solution of Aβ oligomers or Aβ aggregates was added to a glass slide and covered with a cover glass respectively. Fluorescence images were captured on a Leica TCS SP8 confocal laser scanning microscope, with the excitation wavelength at 620 nm, and the emission wavelength from 650 nm to 750 nm.

### ^1^H-NMR studies with KLVFF segment

^1^H-NMR spectrum of DMSO-*d*_6_ solution of KLVFF (2.0 mM) was recorded at 25 °C using 500 MHz Bruker Avance III HD 500 spectrometer followed by addition of probe **2** (2.0 mM) and probe **9** (2.0 mM). The resulting solution was kept at room temperature overnight, and then subjected to ^1^H-NMR spectrum acquirement. The ppm reference peaks were set at 2.49 ppm with DMSO-*d*_6_ as the reference.

### Binding constant (*K*_d_) measurement

The concentration of each probe was determined by weight method. The stock solution of each probe (10 mM in DMSO) was diluted to the concentration of 10 μM using DMSO for the following binding constant measurement.

Various amounts of probes **1**-**9** (10 μM in DMSO) was added (final concentration to be 2.5 nM, 5.0 nM, 10.0 nM, 20.0 nM, 40.0 nM, 60.0 nM, 100.0 nM and 150.0 nM) into 2.0 mL PBS buffer (pH = 7.4) containing 2.5 μM Aβ monomers, oligomers or aggregates respectively. After being mixed with pipette, the fluorescence spectrum of solution was recorded using a HORIBA Jobin Yvon Fluoromax-4 fluorometer. The *K*_d_ value of each probe against different Aβ species was calculated based on the fluorescence enhancement (ΔFI = F_(C(Aβ))_-F_0_, where F_0_ is the fluorescence intensity of Aβ species without probes, and F_(C(Aβ))_ is the fluorescence intensity of Aβ species upon addition with a tested concentration of probe). The *K*_d_ binding curve was generated using Prism 5.0 software with nonlinear one-site binding regression.

### Log*P* measurement

First, the standard curve of each probe was obtained for the following quantification. Second, probe **1**-**9** (0.1 mM) in 1.0 mL octanol with was subjected to partition with 1.0 mL octanol-saturated water. The resulting mixture was stirred vigorously for 5 min, and centrifuged at 3,000 rpm for 5 min. The octanol layer was separated from water layer, and its fluorescence spectrum was recorded. The concentration of each probe in octanol layer was calculated using the equation generated from its standard curve. The concentration in water layer was deduced by subtracting the amount of each probe in octanol layer from its total added amount. The log*P* value was calculated by the ratio of each probe's concentration in octanol layer and water layer respectively.

### Cell culture

U87MG glioblastoma cells and PC-12 cells were purchased from Stem Cell Bank, Chinese Academy of Sciences (Shanghai, China). U87MG glioblastoma cells were grown in high glucose Dulbecco's modified Eagle's medium (DMEM) (Gibco) containing 10% fetal bovine serum (FBS) (Gibco), and 1% penicillin/streptomycin (Gibco) and cultured in a 5% CO_2_ humidified incubator at 37 °C. PC-12 cells were cultured in Roswell Park Memorial Institute 1640 medium (RPMI-1640) supplemented with 10% fetal bovine serum (FBS) (Gibco), and 1% penicillin/streptomycin (Gibco) and cultured in a 5% CO_2_ humidified incubator at 37 °C.

### Cytotoxicity studies

U87MG cells were seeded on flat-bottomed 96-well plates (5000 cells/well) and incubated at 37 °C for 24 h. Varying concentrations of probe **2** or probe **9** (0, 0.25, 0.5, 1.0, 2.5, 5.0, 10 μM) in the DMEM medium were then added. After being incubation for 24 h, 50 μL MTT solution (1 mg/mL in PBS) was added into each well. The cells were kept at 37 °C for another 4 h, and the medium in each well was then removed carefully. The resulting purple crystals in the wells were dissolved by addition of 150 μL DMSO. The absorbance (OD) of formazan at 490 nm in each well was recorded on a microplate reader (Tcan). The absorbance of cells without any treatment (OD_control_) were used as the control, and the percentage of cell viability in each treatment was calculated by dividing OD to OD_control_. Every experiment was repeated three times.

The cytotoxicity of probe **2** and probe **9** towards PC-12 cells were performed by adopting the same way except that the medium was changed with 1640 medium.

### Animal Models

Normal ICR mice (5 weeks, male) and BALB/c mice (5 weeks, female) were purchased from the Model Animal Research Center (MARC) of Nanjing University (Nanjing, China). Transgenic mice (C57BL6, APPswe/PSEN1, 4-14 months old, male), used as Alzheimer's models, and its littermates were also purchased from the Model Animal Research Center (MARC) of Nanjing University (Nanjing, China). All animal experiments were approved by the Institutional Animal Care and Use Committee (IACUC) of Nanjing University.

### Detection of exogenous Aβ species in mouse brain homogenates

A 5-week old ICR mouse was sacrificed. The brain was dissected and homogenized with 2.0 mL PBS buffer (pH = 7.4). 0.1 mL of the resultant homogenate was added to a 96-well plate, followed by the addition of probe **2** (5 μM of final concentration) and the Aβ monomers, oligomers and aggregates (5 μM of final concentration). Then the resulting brain homogenates were recorded using an IVIS Lumina XR III animal imaging system (Caliper LifeSciences, PerkinElmer). The parameter is E_x_/E_m_ = 620 nm/710 nm.

### *In vitro* fluorescent staining of brain slice

Paraffin-embedded 10 μm brain tissue sections from APP/PS1 transgenic mouse (C57BL6, APP/PS1, 14 months old, male) and age-matched wild-type mice (C57BL6, 14 months old, male) were used for *in vitro* fluorescent staining. Before staining, the slices were deparaffinized by washing with ethanol for 5 min after 15 min immersion in xylene. After washing with 50% ethanol, DD water and PBS buffer (pH = 7.4) respectively, the slices were incubated in aqueous solution of each probe (10 μM) for 20 min at room temperature and then washed with ethanol/water (v/v = 50%:50%) followed by washing with PBS buffer (pH = 7.4). After removing the residual liquid with dust free paper, the slice was co-stained with 1% Thioflavin T (30% ethanol solution) for 10 min. Next, the slice was covered with VectaShield mounting media. Florescence images were observed using Olympus VS200 microscope.

### *In vivo* NIR fluorescence imaging

*In vivo* NIR fluorescence imaging was performed using IVIS Lumina XR Ⅲ animal imaging system (Caliper LifeSciences, PerkinElmer). Images for probes** 2**, **4** and **9** were acquired with a 620 nm excitation filter and a 710 nm emission filter. Images for probe **6** were acquired with a 600 nm excitation filter and a 710 nm emission filter. Images for CRANAD-58 were acquired with a 580 nm excitation filter and a 670 nm emission filter. Data analysis was performed using Living Image Software (4.5.2, PerkinElmer, MA, U.S.A.). The heads of 10 months old mice (male transgenic APP/PS1, n = 3 and age-matched male wild-type control mice, n = 3) were shaved before background imaging. The solutions of probe **2**,** 4**,** 6**, **9** and CRANAD-58 (1.0 mg/kg) were freshly prepared in 20% DMSO, 20% cremorphor, and 60% PBS buffer (pH = 7.4), and the solutions were stabilized for 20 min before i.v. injection into mice. Fluorescence signals from the brain were recorded before and at 10, 30, 60, 120, 240 and 360 min after intravenous injection of the probes. To evaluate the imaging results, a region of interest (ROI) was drawn around the brain region. Intensity of brain fluorescence was calculated from the radiant efficiency. Note: For different probes, the mice in its corresponding groups (e.g. APP/PS1 mice group or age-matched control mice group) might be used repeatedly.

The *in vivo* NIR fluorescence imaging with probe **9** in 6-month-old APP/PS1 mice and age-matched control mice was conducted in the same way.

### Thinning skull surgery

The APP/PS1 mouse or age-matched control mouse (14 months old) was anesthetized with a solution of Midazolam (5.0 mg/kg), and Medetomidine (0.5 mg/kg), and a thin-skull imaging window was surgically prepared according to previously reported approach 40.

### Upright fluorescence microscopic imaging

Probe **9** (1.0 mg/kg in a fresh solution containing 20% cremorphor, 20% DMSO and 60% PBS buffer) was injected intravenously at time 0 min by a bolus injection during image acquisition. The fluorescence excitation was 615 nm (ANDOR, Sona). Imaging was performed using an upright microscope (Nikon technologies) equipped with a 4 × water immersion objective (Nikon NIR Apo). Images were collected every 5 seconds per frame 512×512 μm matrix, and last for 20 min. Images were analyzed with ImageJ software.

### Statistical analysis

Statistical comparison between two groups was evaluated by Student's t-test. All the results were analyzed using Prism 7 (Prism GraphPad Software, Inc., San Diego). Results were expressed as mean ± SD, and p < 0.05 was considered statistically significant.

## Results

### Design and Synthesis of NIR Fluorescent probes for Aβ species

Figure 1B illustrates the general design of the NIR fluorescent probes (**1** and** 2**) on the basis of a D-A-D architecture by rationally hybridizing CRANAD-58 and IR-780, a widely used cyanine-based NIR fluorophore with the maximum fluorescence emission at 820 nm 41-44. Considering that the electron-donating ability of the indoline moiety in IR-780 was superior to that of either 4-*N*,*N′*-dimethylaminophenyl or 6-*N*,*N′*-diethylaminopyridyl group in CRANAD-58 45, we envisioned that the presence of 1,3,3-trimethyl-indoline group in probes **1** and **2** could allow a more efficient delocalization of electrons through the π conjugated system, thereby contributing to longer absorption and emission wavelengths than that of CRANAD-58. After screening of probe **2** to be optimum over probe **1**, further modification of probe **2** was then conducted. First, to augment the bathochromic effect and improve binding affinity toward Aβ species, probes **3**-**6** were designed by substituting the 4-*N*,*N′*-dimethylaminophenyl moiety with other stronger electron-donating groups, such as 4-azetidinylphenyl, 4-pyrrolidinylphenyl ring, 4-*N*,*N′*-diethylaminophenly group, and 3- methoxyl-4-*N*,*N′*-dimethylaminophenyl group 46. Second, to elongate the π conjugation for red-shifting fluorescence emission, we designed probe **7** with a 6-*N,N′*-dimethyaminonaphthyl group in placing the 4-*N*,*N′*-diethylaminophenly group of probe **2**. Third, to examine the steric effect of indoline on detecting Aβ species, probe **8** was designed by replacing the 1,3,3-trimethyl-indoline group with *N*-ethyl-3,3-dimethyl-indoline group. Fourth, to optimize the physiological properties for improving fluorescence imaging of Aβ species *in vivo*, probe **9** was designed with introduction of an *N*-methyl-*N′*-hydroxyethyl group 25, 47.

Probes **1**-**9** were synthesized according to the protocol outlined in Scheme S1. Condensation of 2,2-difluoro-1,3-dioxaboryl-pentadione (**A1**) with substituted aromatic aldehyde (**B1**-**8**) in the presence of acetic acid and tetrahydroisoquinoline afforded intermediates (**C1**-**8**). The subsequent condensation with Fischer's aldehyde **D1** or **D2** in acetic anhydride yielded the desired probes **1**-**9** as dark blue solids with purity >97% (Figure S1).

### Investigation of photophysical properties of probes 1 and 2

**Figure 2:**
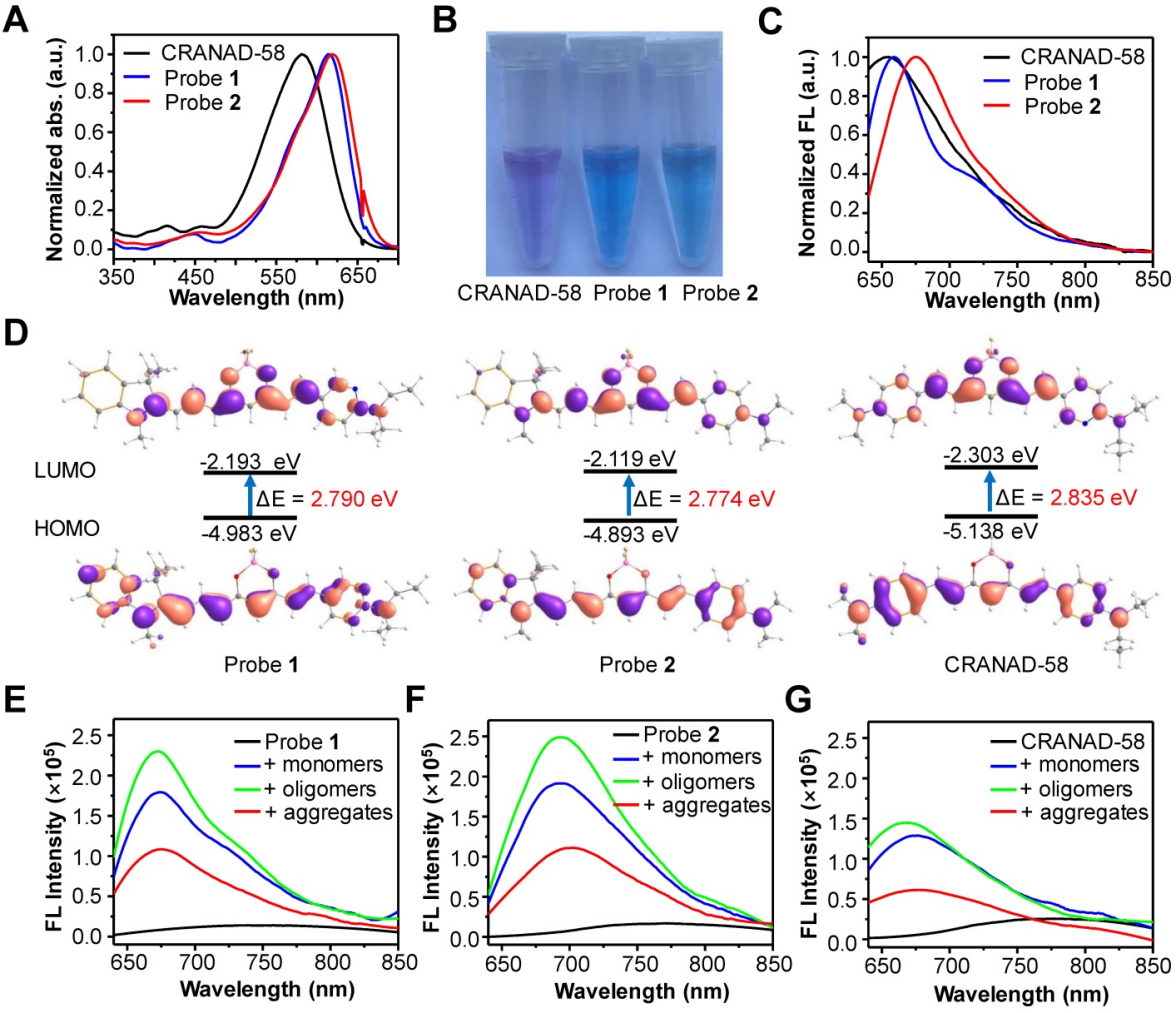
** (A)** Normalized UV-vis absorption (abs.), (B) photograph and **(C)** normalized fluorescence (FL) spectra of CRANAD-58, probes** 1** and **2** in CH_2_Cl_2_. **(D)** Frontier molecular orbitals of the HOMO and LUMO of CRANAD-58, probe** 1** and** 2**. The energy gap between HOMO and LUMO decreased at an order of probe** 2**, probe** 1** and CRANAD-58. **(E, F, G)** FL spectra of probe **1** (E), probe** 2** (F) or CRANAD-58 (G) upon incubation with 250 nM Aβ_42_ monomers, Aβ_42_ oligomers and Aβ_42_ aggregates in PBS buffer, respectively. The concentration of each probe is 250 nM.

We first investigated the optical properties of probes **1** and **2**. As shown in Figure 2A, the maximum UV-vis absorbance of probes **1** and **2** in CH_2_Cl_2_ was found to be 614 nm and 619 nm, respectively, which were more than 30 nm longer than that of CRANAD-58 (λ_abs_ = 581 nm). Owing to the bathochromic shift in UV-vis absorption, both probes **1** and **2** displayed a blue color in CH_2_Cl_2_, whereas CRANAD-58 showed a purple color (Figure 2B). The fluorescence emission of probe **2** in CH_2_Cl_2_ appeared in the NIR region (λ_em_ = 675 nm), which was longer than that of probe **1** (λ_em_ = 659 nm) and CRANAD-58 (λ_em_ = 653 nm) (Figure 2C). These results demonstrate that probe **2** containing the 1,3,3-trimethyl-indoline group displayed a more obvious bathochromic shift in fluorescence emission compared with probe** 1** or CRANAD-58, according with their HOMO-LUMO gaps theoretically calculated (Figure 2D). We then examined the fluorescence emissions of probes **1** and **2** in solvent with different polarity. As expected, their fluorescence was very weak in aqueous solution (e.g. PBS buffer), which shifted blue with fluorescence intensity remarkably increased when the polarity of solvent decreased, similar to that of CRANAD-58 (Figure S2). The subsequent measurement of the absolute quantum yields (*Φ*_f_) showed that the *Φ*_f_ value of probe **2** was ~26.3% in CH_2_Cl_2_, higher than that of probe **1** (~13.2%) or CRANAD-58 (~9.6%), whereas the *Φ*_f_ values of them in PBS buffer were all less than 0.1% (Table S1). As CH_2_Cl_2_ is a typical solvent to mimic the hydrophobic “binding pocket” of the Aβ species 48, we envisioned that such dramatic different* Φ*_f_ values between CH_2_Cl_2_ and PBS buffer might endow probes **1** and **2** “off-on” NIR fluorescence toward Aβ species.

We next tested the fluorescence response of probes **1** and **2** toward both soluble Aβ species (Aβ_42_ monomers and Aβ_42_ oligomers) and insoluble Aβ species (Aβ_42_ aggregates). Akin to CRANAD-58, both probes **1** and **2** displayed weak fluorescence in PBS buffer alone; upon binding to Aβ species, a significant enhancement in fluorescence intensity together with a blue shift in fluorescence emission occurred (Figure 2E-G). There were significantly ~34-fold, ~44-fold and ~15-fold increments in fluorescence intensity upon binding of probe **2** (250 nM) with one equivalent of Aβ_42_ monomers, oligomers and aggregates, respectively, larger than that of probe **1** or CRANAD-58 (Table 1). Moreover, upon interaction with the Aβ_42_ species, the fluorescence wavelengths of probe **2** appeared at 693 nm for Aβ monomers, 692 nm for Aβ oligomers, and 700 nm for Aβ aggregates, which were also revealed from the contour maps of fluorescence spectra (Figure S3). All the fluorescence wavelengths were longer than that CRANAD-58 upon binding with Aβ monomers (λ_em_ = 674 nm), Aβ oligomers (λ_em_ = 667 nm), or Aβ aggregates (λ_em_ = 675 nm). In contrast, the fluorescence wavelengths of probe **1** after binding with the Aβ_42_ species were all much shorter than that of probe **2**, which could be presumably owing to the reduced electron- donating ability of 6-*N*,*N*′-diethylaminopyridyl in probe **1** compared with that of 4-*N*,*N*′-dimethylaminophenyl group in probe **2**, thus weakening the bathochromic effect. These findings accorded with the aforementioned fluorescence emission in CH_2_Cl_2_ (Figure 2C). The examination of binding affinity against Aβ species showed that both probes** 1** and **2** could bind strongly with all the Aβ species, with *K*_d_ values at a nM level, similar to that of CRANAD-58 (Figure S4-6 and Table 1). Considering the longer emission wavelengths and higher fluorescence “turn-on” ratios upon binding with Aβ species, probe **2** deemed to be optimum over probe** 1** and CRANAD-58 for subsequent fluorescence imaging of Aβ species.

### Detection of Aβ species with probe 2* in vitro*

**Figure 3:**
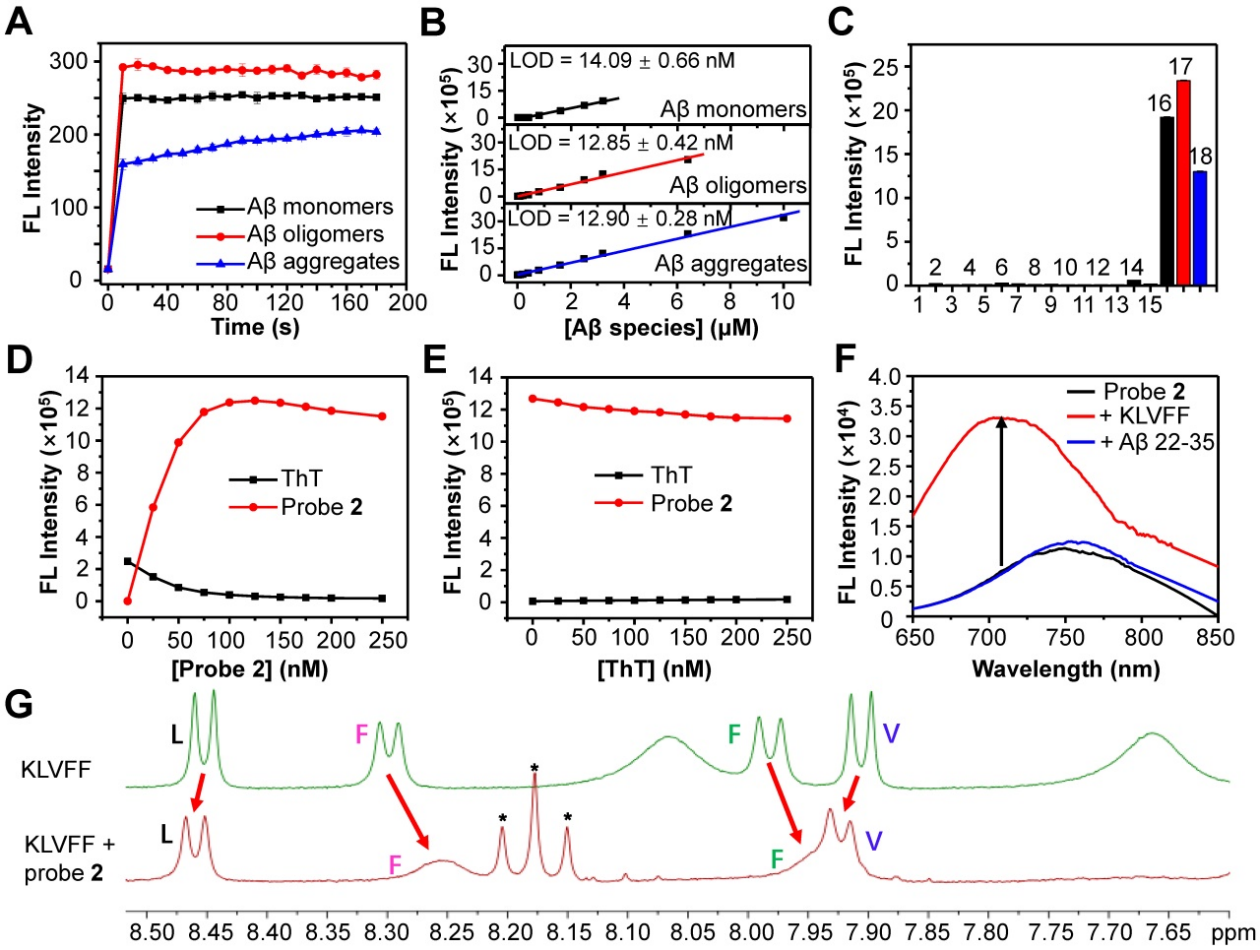
** (A)** FL intensity (λ_ex/em_=620/700 nm) of probe **2** (250 nM) following incubation with 250 nM Aβ_42_ monomers (black), oligomers (red) and aggregates (blue) for 0-180 s. **(B)** Plots of the mean FL intensity of probe **2** (250 nM) versus varying concentration of Aβ_42_ monomers (black), oligomers (red) and aggregates (blue). **(C)** FL intensity of 250 nM probe **2** upon incubation with Aβ42 species (10 µg/ml) and other representative endogenous biological species (10 µg/ml) in PBS buffer (1: PBS, 2: OH· (200 µM Fe^2+^ + 1 mM H_2_O_2_), 3: ^1^O_2_ (1 mM H_2_O_2_ + 1 mM ClO^-^), 4: O_2_^·-^ (100 µM xanthine + 22 mU xanthine oxidase), 5: H_2_O_2_ (1 mM H_2_O_2_), 6: hMAO-A, 7: β-Galactosidase, 8: AChE, 9: BuChE, 10: _L_-Cysteine, 11: GSH, 12: Cytochrome C, 13: Vitamin C, 14: Amylin, 15: BSA, 16: Aβ_42_ monomers, 17: Aβ_42_ oligomers, 18: Aβ_42_ aggregates).** (D, E)** Change in the FL intensities of ThT (λ_ex/em_ = 445/482 nm) and probe **2** (λ_ex/em_ = 620/700 nm) upon titrating (D) probe **2** to ThT-Aβ_42_ aggregates mixture or (E) ThT to probe **2**-Aβ_42_ aggregates mixture in PBS buffer. **(F)** FL spectra of probe** 2** (black) and probe **2** (250 nM) incubating with KLVFF (250 nM) or Aβ22-35 (250 nM) peptides. **(G)** Comparison of the ^1^H-NMR spectra (DMSO-*d*_6_, 500 MHz) of KLVFF (2.0 mM) in the presence (red) or absence (green) of probe **2** (2.0 mM). Red arrows indicated the change of chemical shifts of the amide protons of L, V and F residues. * indicating the ^1^H-NMR peaks from probe **2**.

We then chose probe **2** as the optimum to investigate the ability to detect Aβ species *in vitro*. First, we measured the binding kinetics of probe **2** against Aβ monomers, oligomers and aggregates. As shown in Figure 3A, the fluorescence intensity of probe **2** (250 nM) immediately increased upon mixing with each Aβ species (250 nM), which could reach the plateau within 10 s for Aβ monomers and Aβ oligomers, and about 150 s for Aβ aggregates, indicating that the binding of probe **2** with the Aβ species was kinetically fast. Second, the limit of detection (LOD) of probe **2** toward different Aβ species was determined by measuring the fluorescence spectra of probe **2** (250 nM) after being incubated with varying concentrations of Aβ species (0-15 μM) (Figure 3B and Figure S7). As shown in Figure 3B, the fluorescence intensity of probe **2** could linearly correlate with Aβ monomers, oligomers and aggregates at a concentration range of 100 nM-6.4 μM, 50 nM-3.2 μM and 100 nM-10 μM, respectively. The LOD (3σ/k) was then calculated to be ~14.1, ~12.8 and ~12.9 nM for the monomers, oligomers and aggregates, respectively, which were comparable to that of other previously reported fluorescent probes for Aβ aggregates 28, 49-53. Third, the examination of the selectivity toward the Aβ proteins over other representative endogenous species, including _L_-Cysteine, glutathione, vitamin C, Cytochrome C, BSA, AChE, BuChE, Amylin, hMAO-A, β-Galactosidase, and reactive oxygen species (hydroxyl radical, singlet oxygen, superoxide radical, and H_2_O_2_) showed that strong fluorescence could be observed only in the presence of Aβ monomers, oligomers or aggregates; negligible fluorescence appeared toward other examined species, especially Amylin or BSA, which were found to be notorious species potentially competing with the Aβ species in the brain (Figure 3C). These results suggest that probe **2** hold high kinetics, sensitivity and specificity to detect both soluble and insoluble Aβ species. Moreover, the confocal fluorescence microscopy imaging of Aβ oligomers or aggregates stained with probe **2** showed the occurrence of bright NIR fluorescence, which could clearly delineate the different morphology of Aβ oligomers and aggregates, confirming the good ability for fluorescence imaging of Aβ species (Figure S8).

To further gain insights into the binding sites between probe **2** and Aβ species, three different experiments were then carried out. First, competition assay by titrating probe **2** against ThT (250 nM, a gold standard agent for the Aβ plaques) bound Aβ_42_ fibrillar aggregates (2.5 μM) showed that the fluorescence of ThT at 482 nm decreased and nearly returned to the background upon gradual addition of probe **2**, while the fluorescence of probe **2** at ~700 nm increased concurrently, suggesting effective displacement of ThT from the Aβ_42_ aggregates by probe **2** (Figure 3D). By contrast, a reverse competition test by titrating ThT against probe **2** (250 nM) bound Aβ_42_ fibrillar aggregates (2.5 μM) showed that the strong NIR fluorescence of probe **2** was kept, while the fluorescence of ThT was negligibly enhanced, suggesting ineffective displacement of probe **2** from the Aβ_42_ aggregates by ThT (Figure 3E). These findings imply that (1) probe **2** and ThT could probably bind to the same sites in the Aβ_42_ aggregates, and (2) probe **2** showed a much higher binding affinity than that of ThT toward the Aβ_42_ aggregates. Second, we investigated whether probe** 2** could bind with the Aβ16-20 segment (KLVFF) and induce fluorescence enhancement as it has been previously reported that the KLVFF sequence was the major hydrophobic fragment for ThT binding. After incubating probe **2** (250 nM) with the KLVFF peptide (250 nM) in a PBS buffer, the fluorescence of probe **2** blue shifted to ~700 nm and the intensity increased about 4.4-fold, while the incubation of probe **2** with the KLVFF free segment Aβ 22-35 (EDVGSNKGAIIGLM) showed neither blue shift nor intensity enhancement in fluorescence, suggesting that the KLVFF fragment was probably the core site for probe **2** binding (Figure 3F). Third,^ 1^H-NMR spectroscopy showed obvious changes in the chemical shifts of amide protons of L, V and F residues in the solution containing probe **2** and KLVFF peptide, supporting the interaction of probe **2** with the hydrophobic KLVFF peptide fragment (Figure 3G). We envisioned that the insertion of probe **2** into the hydrophobic site of Aβ proteins could help to restrain double-bond rotation and increase radiative decay rate of probe **2** after excitation, consequently enhancing fluorescence emission. This phenomenon was further validated by the obvious blue-shift and enhancement in fluorescence emission of probe **2** when the solvent viscosity increased (Figure S9). In all, these results revealed that the KLVFF core fragment was likely the key site for the interaction between probe **2** and the Aβ species, effectively switching on the NIR fluorescence of probe **2**.

To examine the ability of probe **2** for the fluorescence imaging of endogenous Aβ species in biological environment, we first demonstrated that probe **2** hold good stability in PBS buffer (Figure S10), mouse serum (Figure S11) and good photostability under continuous irradiation of lamplight (Figure S12). Next, the biocompatibility of probe** 2** was examined to show negligible cytotoxicity against human glioma U87MG cells and PC-12 cells after being incubated with probe **2** at 10 μM for 24 h (Figure S13). Furthermore, we investigated the ability of the probe to detect exogenous Aβ species in mouse brain homogenates via fluorescence imaging. As shown in Figure S14, mouse brain homogenates containing 5 μM Aβ monomers, Aβ oligomers or Aβ aggregates displayed much brighter NIR fluorescence images compared to that of native mouse brain homogenates after its incubation with probe **2** (5 μM), indicating that probe **2** could well detect the exogenous Aβ species in brain homogenates.

### NIR fluorescence imaging of Aβ species* in vivo*

**Figure 4:**
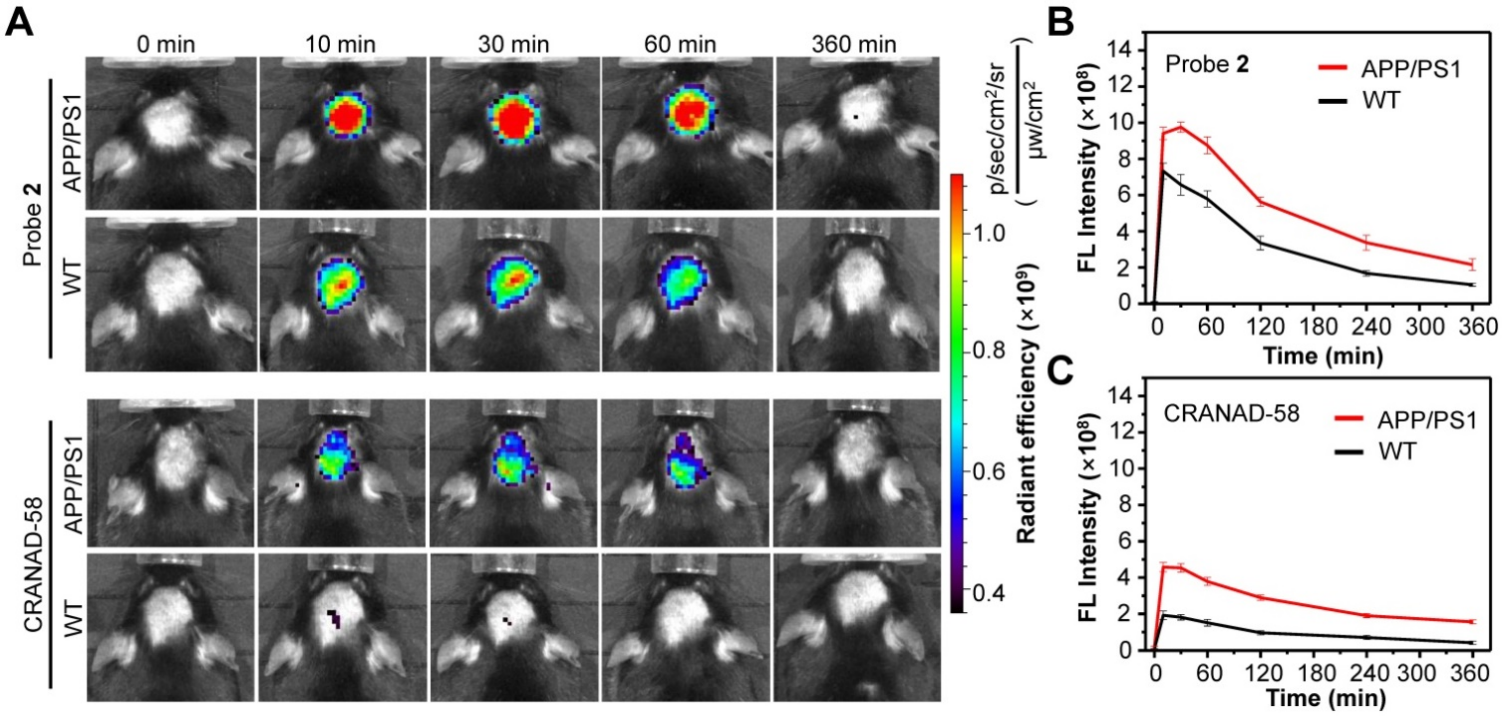
Longitudinal NIR FL images **(A)** and mean brain FL intensities of APP/PS1 transgenic mice and WT mice (10-month old) following i.v. injection of probe **2 (B)** and CRANAD-58 **(C)** (1.0 mg/kg) at 0, 10, 30, 60, and 360 min. Data are mean ± standard deviation (S.D.) (n = 3).

Having demonstrated the good capacity of probe **2** for the detection of exogenous Aβ species, we then applied probe **2** to detect endogenous Aβ species via NIR fluorescence imaging. First, the log*P* values of probe **2** was measured, which was found to be ~2.31, ensuring a proper lipophilicity to cross the BBB and enter brain (Table S1). Then, probe** 2** or CRANAD-58 (1.0 mg/kg) was intravenously (i.v.) injected into three 10-month-old male APP/PS1 double transgenic mice (APP/PS1) and three age-matched WT mice, and fluorescence images of mouse brain were longitudinally acquired. As shown in Figure 4A (More imaging details are shown in Figure S15 and Figure S16), bright fluorescence appeared in the brain of APP/PS1 mice at 10 min post injection of probe** 2** or CRANAD-58, and the fluorescence intensity maximized at 30 min, which then decreased thereafter. APP/PS1 mice showed significantly brighter brain fluorescence images than that of WT mice; the average fluorescence intensity was ~1.3-fold higher in the APP/PS1 mice brains than that of WT mice at 10 min post injection of probe** 2**, which increased to ~1.5-fold at 30 min and ~2.0-fold after 4-6 h (Figure 4B). It was also found that the brain fluorescence in probe** 2**-treated APP/PS1 mice was much brighter than that in mice following i.v. injection of a same dosage of CRANAD-58 (1.0 mg/kg), presumably owing to that probe **2** possesses longer fluorescence emission and higher *Φ*_f_ value in relative to CRANAD-58 (Table S1), which could enhance penetration depth and improve sensitivity for *in vivo* imaging. However, compared to probe **2**, CRANAD-58 also produced darker brain fluorescence in the WT mice; thereby, the brain fluorescence intensity in the APP/PS1 mice was also significantly higher than that in WT mice (Figure 4C), consistent to the results reported by Ran 18. These findings indicate that probe **2** could readily cross the BBB and produce strong NIR fluorescence to differentiate APP/PS1 AD mice from WT mice after i.v. injection into mice, which was similar to CRANAD-58.

**Figure 5:**
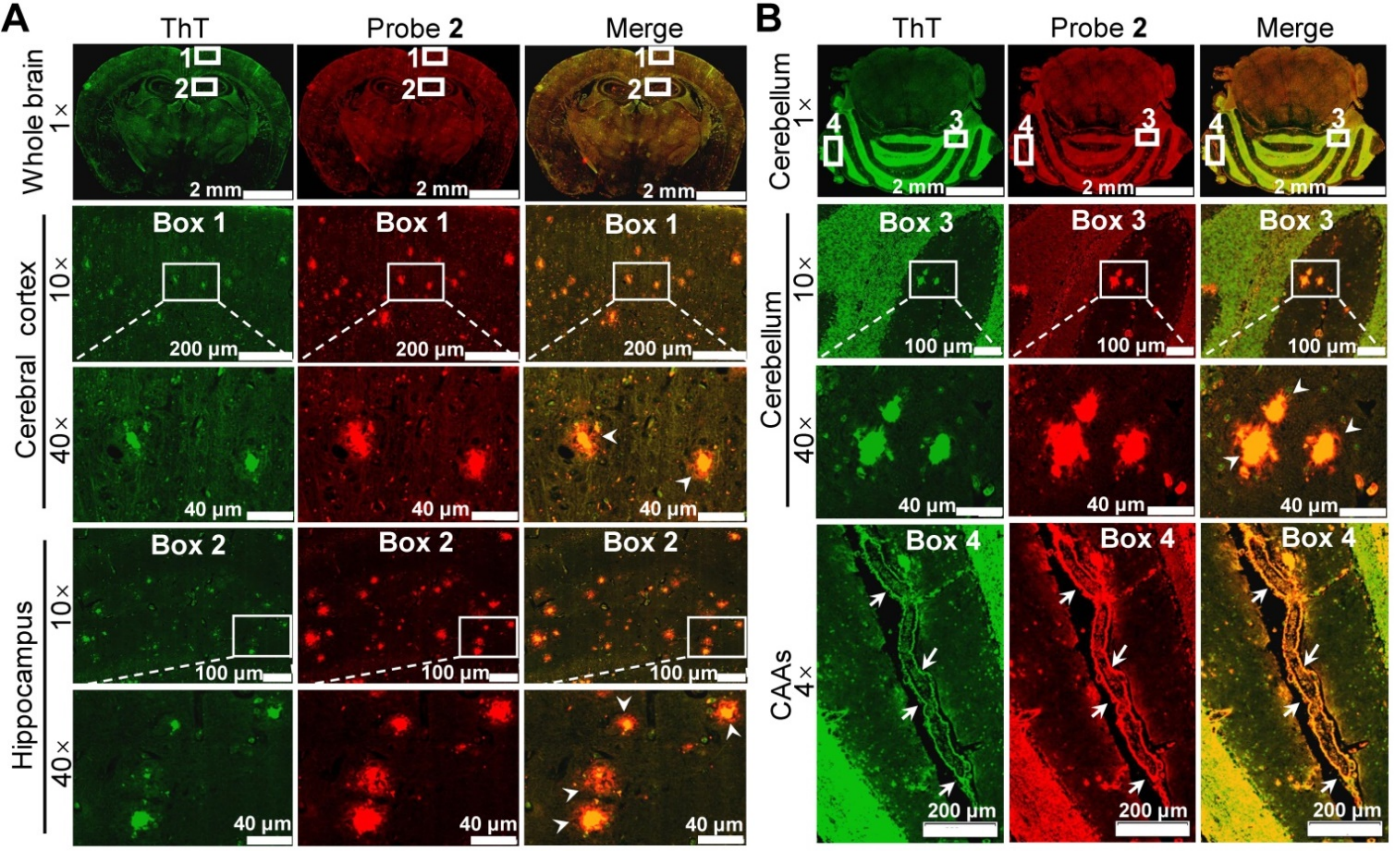
*Ex vivo* histological staining of the **(A)** cerebrum and **(B)** cerebellum tissues slices resected from an APP/PS1 mouse at 30 min after i.v. injection of probe **2** (1 mg/kg). The Aβ plaques in both brain tissue slices were further confirmed by staining with ThT (green). Boxes 1 and 2 in figure (A) indicated enlarged cerebral cortex and hippocampus, respectively; boxes 3 and 4 in figure (B) indicated enlarged cerebellar corpus and CAAs. The white arrowheads showed that the enhanced fluorescence of probe **2** appeared both in the core and the peripheral areas of the Aβ plaques in both cerebrum and cerebellum tissues. White arrows in enlarged box 4 showed the presence of CAAs in the cerebral vessels.

To validate whether the enhanced brain fluorescence in APP/PS1 mice was due to the binding of probe **2** to the upregulated Aβ species, the brains of 14-month-old APP/PS1 and WT mice were resected 30 min after probe **2** injection, and the brain tissue slices were then cut and co-stained with ThT. Fluorescence imaging of the brain tissue slices showed that bright fluorescent dots appeared both in hippocampus and cerebral cortex of the APP/PS1 mouse brain tissue slices, which colocalized well with the fluorescence of ThT (Figure 5A), whereas no similar fluorescent dots could be found in the WT group (Figure S17). Additionally, intensive fluorescent dots could also be observed in the cerebellum area from the APP/PS mouse (Figure 5B), not from the WT mouse (Figure S18). These results confirmed that probe **2** could selectively stain the endogenous Aβ plaques, thus lighting up the AD mouse brain. Note that the green fluorescence of ThT was largely located in the core of the plaques where the Aβ aggregates deposit, whereas the enhanced fluorescence of probe **2** appeared both in the core and the peripheral areas of the Aβ plaques where the Aβ oligomers are likely resided. These findings suggest that probe **2** could well label the endogenous Aβ aggregates and Aβ oligomers in the Aβ plaques, which was advantageous over ThT. Moreover, we found that probe **2** could also efficiently highlight the cerebral amyloid angiopathies (CAAs) (Enlarged box 4 in Figure 5B), which is characterized by depositing Aβ species at the exterior of brain arteries and is closely related with pathological dementia 38, 54. To further verified the staining of CAAs, another 10-month-old APP/PS1 mouse and age-matched WT mouse was i.v. injected with probe **2** respectively. The brain of each mouse was resected at 30 min post injection and then cut into brain slice, which was co-stained with ThT and AlexaFluor 488-labeled CD31 antibody respectively. As can be seen in Figure S19A, probe **2** could clearly light up the plaques distributed in the walls of brain vessels, while there were no fluorescent spots observed in WT mouse (Figure S19B). Taking together, the *ex vivo* histological imaging could well support the *in vivo* fluorescence imaging results, demonstrating that probe **2** was applicable for the noninvasive fluorescence imaging of Aβ species in APP/PS1 AD mice.

### Chemical modification of probe 2

Encouraged by the above results, further modification of probe **2** to obtain probes **3**-**9** was performed (Figure 1B), endeavoring to improve the photophysical and physiochemical properties for* in vivo* fluorescence imaging of Aβ species. As shown in Figure S20 and Table S1, the absorbance and NIR fluorescence emission wavelengths of probes** 3**-**9** in CH_2_Cl_2_ were close to that of probe **2**. Among them, probe **6** with the 3-methoxyl-4-*N*,*N′*-dimethylaminophenyl group showed a much longer fluorescence emission wavelength (λ_em_ = 706 nm) than that of probe** 2** (λ_em_ = 675 nm) in CH_2_Cl_2_. As with probe **2**, they all showed an obvious solvatochromic effect on fluorescence emission (Figure S21). The subsequent examination of the fluorescence response toward Aβ species showed that their weak NIR fluorescence in PBS buffer could be remarkably switched on after binding with Aβ monomers, Aβ oligomers and Aβ aggregates, respectively (Figure S22). Probe **4** with the 4-pyrrolidinylphenyl ring showed the highest fluorescence turn-on ratio toward Aβ monomers (~51-fold at 702 nm) and Aβ oligomers (~74-fold at 696 nm), and approximately ~20-fold enhanced fluorescence (708 nm) toward Aβ aggregates among all these compounds (Table 1).

We then examined their binding affinity toward Aβ monomers, Aβ oligomers and Aβ aggregates, respectively (Figure S23-29 and Table 1). The *K*_d_ values of them were mostly at the nM level, indicating that these probes hold good binding affinities toward Aβ species. It was notable that probe **6** with a 3-methoxy group as the *H*-bond acceptor exhibited higher binding affinities toward Aβ monomer (*K*_d_ = 3.01 ± 0.41 nM), Aβ oligomer (*K*_d_ = 25.62 ± 1.54 nM) and Aβ aggregates (*K*_d_ = 13.51 ± 0.73 nM) than those of probe **2**, presumably because that the introduction of an *H*-bond acceptor could facilitate to form hydrogen bond with the engaged Aβ proteins. Similar results were also observed for probe **9**, where the introduction of a hydrophilic hydroxyethyl group could not only help to lower the log*P* value to 2.14, but also facilitate to act as an *H*-bond acceptor to increase binding affinity against Aβ oligomers (*K*_d_ = 36.59 ± 2.69 nM) and Aβ aggregates (*K*_d_ = 14.57 ± 1.27 nM) as compared with probe **2**. Additionally, probe **7** containing the 6-*N,N′*-dimethyaminonaphthyl group showed longer fluorescence wavelengths after binding with each Aβ species in relation to that of probe **2** as a result of the elongated π conjugation system, however, the *K*_d_ values against the Aβ species were higher than that of probe **2** (Table 1). These results suggest that the introduction of a bulky donor group could probably attenuate the binding affinity toward Aβ species, which was also observed for probe** 8** containing the *N*-ethyl-3,3-dimethyl-indoline group.

### *In vivo* test of probes 4, 6 and 9

**Figure 6:**
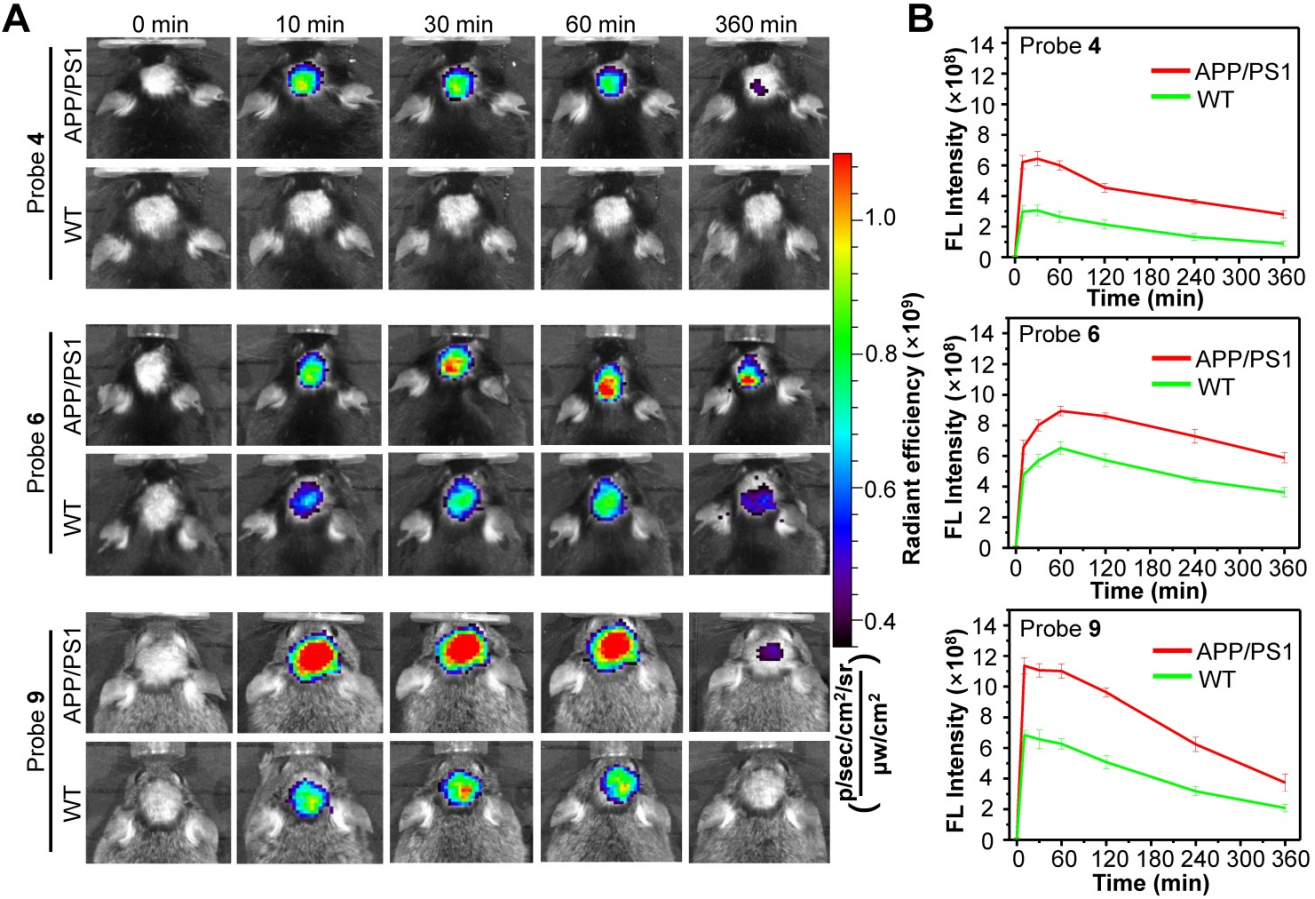
** (A)** Representative longitudinal FL images of APP/PS1 transgenic and WT mice (10-month old) following i.v. injection of probe **4**, **6** or **9** (1.0 mg/kg) at 0, 10, 30, 60, and 360 min. **(B)** Quantitative analysis of the brain FL intensities of the APP/PS1 and WT mice at indicated time. Data are mean ± S.D. (n = 3).

On the basis of the *in vitro* results, we next chosen probe **4** with optimum fluorescence enhancement, probe **6** with the highest binding affinity against each Aβ species, and probe **9** with the lowest log*P* value for *in vivo* studies, aiming to improve visualization of endogenous Aβ species in living AD mice. First, *in vitro* fluorescence staining of resected brain tissue slices from APP/PS1 AD and WT mice showed that these three probes could well highlight the Aβ plaques in the cerebral cortex and hippocampus of APP/PS1 mouse (Figure S30, S32 and S34), not in WT mouse (Figure S31, S33 and S35). Then, probes **4**, **6** and **9** were each i.v. injected into male APP/PS1 AD mice and age-matched WT mice, and the brain fluorescence images were acquired at 0, 10, 30, 60 and 360 min post injection. As with probe **2**, the APP/PS1 mice displayed much brighter brain fluorescence than that of WT mice at 10 min post injection of each probe (Figure 6, More imaging details are shown in Figure S36, S37 and S38), with average brain fluorescence intensity considerably higher than that from the age-matched WT mice, suggesting that these three probes could also cross the BBB and efficiently differentiate the APP/PS1 AD mice from WT mice (Figure 6). Among them, probe **4** displayed relatively weaker brain fluorescence both in APP/PS1 and WT mice. However, there was significant ~2.0-fold higher fluorescence intensity in the brain of APP/PS1 mice compared to that in the WT mice at 10 min, which increased to ~2.8-fold at 60 min and ~3.2-fold at 6 h. These differences in brain fluorescence between APP/PS1 and WT mice achieved by probe **4** were larger than that of probes **2**, **6** and **9**, presumably owing to that probe **4** hold much higher fluorescence turn-on ratios toward Aβ species compared to the other probes (Table 1). It was found that though probe **6** displayed the best binding affinities against each Aβ species, it took about 60 min to reach the maximum brain fluorescence both in APP/PS1 and WT mice, much longer than that of probe **2** and **4** (~30 min for them). In addition, probe **6** also showed a slower washout in the brain compared to the other probes, resulting in a narrower discrepancy between APP/PS1 and WT mice. In contrast, probe **9** hold the fastest kinetics (~10 min) to achieve the maximum brain fluorescence signal in the APP/PS1 mice, and produced higher brain fluorescence intensity compared to probe **2** or CRANAD-58. Moreover, a significantly ~1.7-fold higher brain fluorescence intensity in APP/PS1 mice relative to that in WT mice had occurred at 10 min, which was higher than that achieved by probe **2** (~1.3-fold at 10 min). Such increases in both maximum brain fluorescence intensity and discrepancy between APP/PS1 and WT mice at early time point (e.g., 10 min) suggest that probe **9** could be advantage over probe **2** for fluorescence imaging of Aβ species *in vivo*, presumably owing to the reduced log*P* value that potentially improves the physiochemical properties and enhances brain uptake. In addition, Figure S39 illustrated that probe **9** also produced a significantly ~1.5 fold higher signal intensity in APP/PS1 mice to that of WT mice to detect soluble Aβ species in 6-month-old APP/PS1 mice at 10 min. Importantly, probe **9** showed similar *in vitro* properties to that of probe **2** (Figure S12, S40, S41 and S42). The isothermal titration calorimetry experiment 55, 56 illustrated that per one probe** 9** bound with ~1.22 number Aβ monomers, ~0.0623 number Aβ oligomers and ~0.161 number Aβ aggregates respectively (Figure S43). Furthermore, we examined the blood half-life (*t*_1/2_) of probe **9** to be ~13.1 min (Figure S44). The biodistribution studies suggested that probe **9** possessed high brain uptake (Figure S45) and was eliminated mainly via hepatobiliary system (Figure S46), which was validated by the primary fecal metabolic pathway (Figure S47). As shown in Figure S48 and Figure S49, probe **9** also showed a good biocompatibility *in vivo*.

### Dynamic fluorescence imaging of Aβ plaques in mouse brain using probe 9

**Figure 7:**
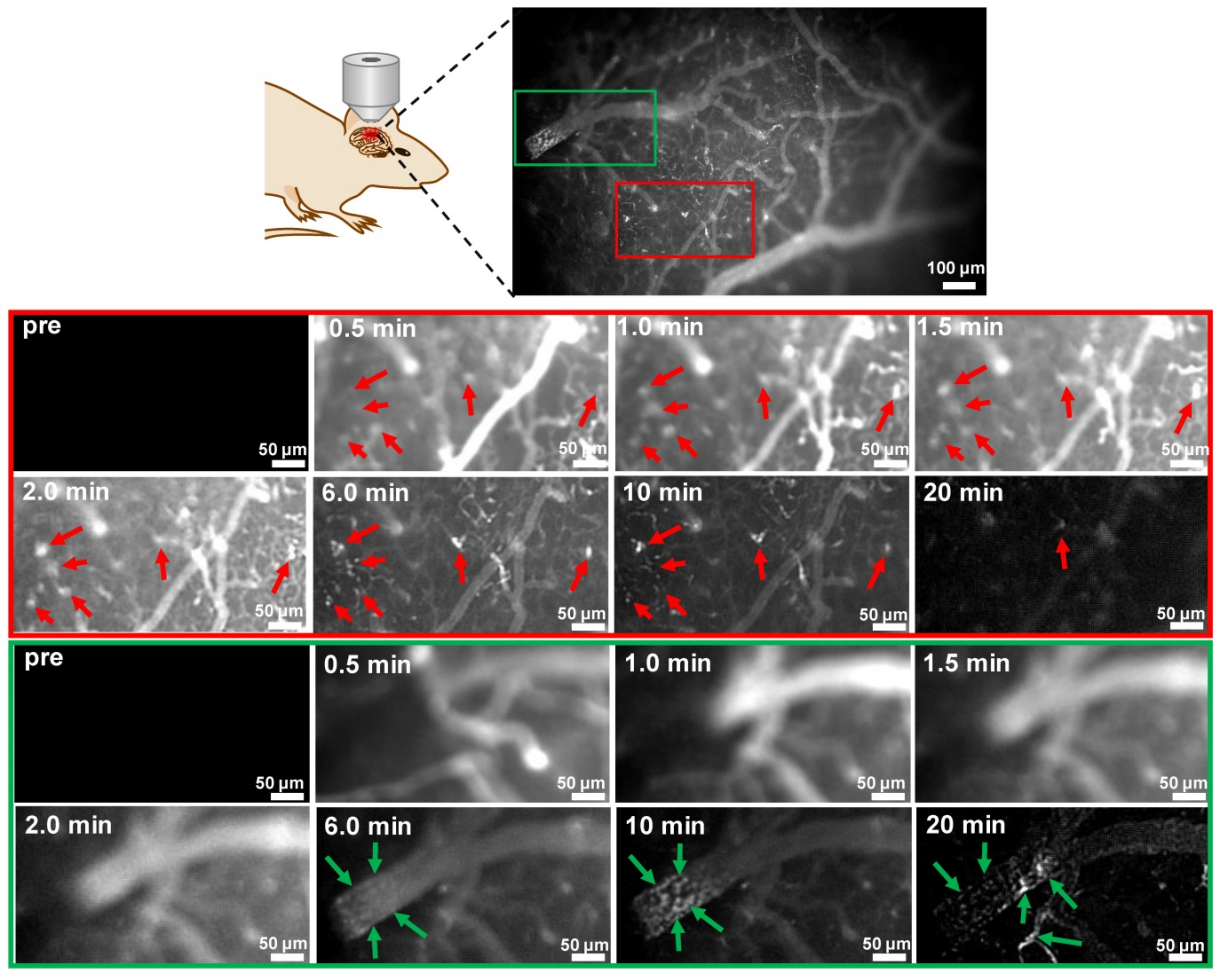
Dynamic FL imaging of Aβ plaques in the brain of a skull-thinning 14-month-old APP/PS1 mouse following i.v. injection of probe **9** (1.0 mg/kg). The fluorescence images were monitored every 5 s on an upright fluorescent microscope. Red and green rectangles indicated enlarged brain parenchyma area and cerebral vessel, respectively. Red arrows indicated the Aβ plaques in the brain parenchyma; green arrows indicated the CAAs in the cerebral vessel.

Taking the advantage of fast kinetics to produce bright brain fluorescence offered by probe **9** due to its improved physiological properties, dynamic visualization of Aβ plaques in the brains of mice was then conducted on an upright microscope. Probe **9** (1.0 mg/kg) was i.v. injected into a skull-thinning 14-month-old APP/PS1 mouse and an age-matched WT mouse respectively, and the brain fluorescence was sequentially acquired in the first 20 min. As shown in Figure 7 and the time-lapse movies (Movies S1 and 2), AD mouse brain showed bare autofluorescence in the NIR region before administration of probe **9**; however, after injection of probe **9**, bright NIR fluorescence could be immediately observed in the blood vessels and brain parenchyma of the APP/PS1 mouse, suggesting that probe** 9** could rapidly cross the BBB and enter the parenchyma. Within 30 s, the appearance of sporadic dot fluorescence indicated that the Aβ plaques in the brain parenchyma could be partially labeled. After that, the dot fluorescence became more apparent and the intensity reached the maximum at approximately 2 min, suggesting that the parenchymal Aβ plaques could be nearly completely stained in 2 min. Notably, the plaques' fluorescence could be sustained for over 10 min, while the vessels' fluorescence decayed from the circulation and clearance of probe **9**, which could produce distinct fluorescence to visualize the distribution of Aβ plaques in the brain parenchyma. In addition to parenchymal Aβ plaques, CAAs in the large vessels could also be labeled and clearly pinpointed by probe **9** after 10 min. After 20 min, the bright plaques' fluorescence in the parenchyma decreased significantly, presumably owing to the clearance of probe **9** from the brain parenchyma, while CAAs' fluorescence remained visible throughout the vessels. In contrast, neither parenchymal Aβ plaques' fluorescence nor CAAs' fluorescence appeared in the age-matched WT mouse during the time course of imaging due to the lack of Aβ plaques both in brain parenchyma and blood vessels (Figure S50), according to the results from both *in vivo* fluorescence imaging of mice (Figure 6) and *in vitro* fluorescence staining of resected brain tissue slices (Figure S34 and Figure S35). These dynamic epifluorescence imaging results intuitively suggested that probe **9** could rapidly cross the BBB and efficiently label both the parenchymal and angiopathic Aβ plaques in APP/PS1 AD mouse, not WT mouse, supporting the high potential of our designed probes for noninvasively detecting Aβ deposits *in vivo*.

### The attenuation effect of probe 9 on the aggregation of Aβ monomers

As mentioned above, probe **9** possessed good binding affinity to Aβ monomers (*K*_d_ = 11.16 ± 0.79 nM) and oligomers (*K*_d_ = 36.59 ± 2.69 nM), it is crucial to assay whether the binding could slow or even prevent the propagation of soluble Aβ species into Aβ aggregates. The western blot analysis (Figure S51) demonstrated that probe **9** was able to attenuate the aggregation of Aβ monomers in a concentration-dependent manner and probe **9** exhibited a significantly higher attenuation effect than CRANAD-58. There was no significant amount of high molecular weight species observed on the SDS-PAGE gel, presumably due to that Aβ42 could aggregate fast into insoluble species that are too large to enter the gel 18.

## Discussion

As a good NIR fluorescent probe for Aβ species, the following properties should be satisfied: (1) high specificity and affinity to Aβ species, (2) reasonable lipophilicity (log*P* between 1 and 3) to guarantee rapid Blood-Brain-Barrier (BBB) penetrability, (3) the “turn-on” fluorescence emission wavelength >650 nm to minimize background fluorescence from brain tissue, (5) high quantum yield, (6) low nonspecific binding, (7) reasonable stability, (8) straightforward synthesis, and most importantly, (9) upon binding to Aβ species, a significant change in fluorescent properties should be observed. To date, a variety of fluorescent probes able to target Aβ species have been reported, such as AOI-987 57, NIAD-4 35, CRANAD-2 17, BAP-1 36, THK-265 58, PAD-1 59, CQ 30, BD-Oligo 60, F-SLOH 61, QAD-1 33, QM-FN-SO_3_ 62, TM-1 31, DANIRs 22-27, 32, 37, CAQ 63, PTO-29 21, PTO-41 47, CRANAD-3 19, CRANAD-102 20 and CRANAD-58 18. Nevertheless, none of them meet all of these criteria due to some potential shortcomings, such as limited BBB permeability of charged molecules, shallow penetration depth or autofluorescence resulting from relative short fluorescence wavelength, slow binding kinetics and relative high *K*_d_ values. In addition, some of these reported probes are only responsive to insoluble Aβ aggregates and plaques on the late stage of AD (Table S2), which is not conducive to the early diagnosis and treatment of the neurodegenerative disease. Among these probes, CRANAD-58 was reported to have the capability to detect both soluble and insoluble Aβ species, which is vital to monitor the changes of Aβ species during the pathological course of the disease. Though CRANAD-58 has shown promise to noninvasively detect Aβ species in transgenic AD mice, the emission wavelength (~670 nm) is still too short for further applications. Therefore, new probes with longer emission wavelength and improved physiological properties are in highly demand.

In the research, through rationally hybridizing CRANAD-58 and NIR cyanine IR-780 fluorophore, we have developed nine D-A-D type of NIR fluorescent probes (**1**-**9**) for noninvasive imaging of both soluble and insoluble Aβ species in living APP/PS1 AD mice. Based on the modification step-by-step, probe **9** was demonstrated to be optimum probe due to its fast kinetically binding with Aβ species (< 120 s), high sensitivity and selectivity towards Aβ species. Owing to a more efficient delocalization of electrons from 1,3,3-trimethyl-indoline group, probe **9** showed longer emission wavelength (Aβ monomers: 690 nm v.s. 674 nm, oligomers: 688 nm v.s. 667 nm, aggregates: 697 nm v.s. 675 nm) upon interaction with Aβ species, compared with that of CRANAD-58. In addition, probe **9** showed significant fluorescence intensity enhancement upon binding to Aβ species acted as 1:1 molar ratio, which indicated a “turn-on” fluorescent response towards Aβ species. Moreover, probe **9** possesses desirable lipophilicity (log*P* = 2.14), which significantly enhanced the brain uptake. It should be noticed that probe **9** (~20.31) also have a higher quantum yield than CRANAD-58 (~9.58) in CH_2_Cl_2_, which is a typical solvent to mimic the hydrophobic microenvironment of the Aβ species. The western blot analysis indicated that probe **9** possessed significantly higher attenuation effect than CRANAD-58 in concentration-dependent manner, suggestive of its potential for the treatment of AD. Based on these findings, probe **9** hold the following outstanding features: (1) To be an excellent NIR fluorescence probe, probe **9** meets the properties required for monitoring soluble and insoluble Aβ species noninvasively *in vivo*, and thus possessed great potential to early definitive diagnosis of AD and monitoring anti-AD drugs' efficacy *in vivo*; (2) Probe **9** showed red-shifted emission wavelength than CRANAD-58, which was conducive to enhance penetration depth and improve sensitivity for *in vivo* imaging, especially for the noninvasive fluorescence imaging of brain, where the intact cranium existed; (3) Probe **9** also showed higher quantum yield than CRANAD-58, which indicated that a lower administration dose for probe **9** than that of CRANAD-58 was viable in the *in vivo* application and thus could reduce the neurotoxicity resulting from the relative high injection dose; (4) Probe **9** also showed significantly higher attenuation effect on the aggregation of Aβ monomers than CRANAD-58 in concentration-dependent manner, indicated that probe **9** might be used as imaging/therapeutic agent.

## Conclusion

In conclusion, we have developed nine D-A-D type of NIR fluorescent probes (**1**-**9**) for noninvasive imaging of both soluble and insoluble Aβ species in living APP/PS1 AD mice. We first designed probes **1** and **2** by rationally hybridizing CRANAD-58 and NIR cyanine IR-780 fluorophore, and demonstrated that probe **2** deemed to be optimum over probe **1** and CRANAD-58 because it showed a higher fluorescence quantum yield (*Φ*_f_ = ~26.3%) and longer fluorescence emission (λ_ex/em_ = 619/675 nm) than probe **1** (*Φ*_f_ = ~13.2%, λ_ex/em_ = 614/659 nm) or CRANAD-58 (*Φ*_f_ = ~9.6%, λ_ex/em_ = 581/653 nm) in CH_2_Cl_2_. Probe **2** also showed larger fluorescence turn-on ratios upon binding with one equivalent of Aβ monomers, oligomers and aggregates, respectively, with the fluorescence emissions all extending to over 690 nm, longer than that of CRANAD-58 (~670 nm). Titration and ^1^H-NMR spectroscopic studies demonstrated that probe** 2** could probably bind to the KLVFF fragment in the Aβ species, allowing fast binding kinetics, good binding affinity, high sensitivity and specificity to detect both soluble and insoluble Aβ species. After i.v. injection, it can rapidly penetrate the BBB and produce approximately 1.3-fold higher brain fluorescence in APP/PS1 AD mice than that in the age-matched WT mice at 10 min, which increased to ~1.5-fold at 30 min. The *ex vivo* fluorescence imaging of brain tissue slices resected from APP/PS1 mouse at 30 min post i.v. injection showed that probe **2** could label Aβ species deposited in hippocampus, cerebral cortex, cerebellum area and cerebral vessels. In order to augment the bathochromic effect and improve the *in vivo* imaging performance, further structural modification of probe **2** to afford probes **3**-**9** was then conduced. These seven probes displayed different fluorescence response and binding affinities toward Aβ monomers, oligomers and aggregates. Among them, probe **4** displayed larger fluorescence turn-on ratio than probe **2** upon incubation with Aβ species *in vitro*, which could produce significantly ~2.0-fold higher fluorescence intensity in the brain of APP/PS1 mice compared to that in the WT mice at 10 min. Probe** 6** with a 3-methoxy group as the* H*-bond acceptor exhibited higher binding affinities toward Aβ monomer (*K*_d_ = 3.01 ± 0.41 nM), Aβ oligomer (*K*_d_ = 25.62 ± 1.54 nM) and Aβ aggregates (*K*_d_ = 13.51 ± 0.73 nM) compared to other probes. However, *in vivo* imaging results showed a longer time (~60 min) to peak brain fluorescence and slower washout from brain. It was notable that probe **9** with a slightly reduced log*P* value hold the fastest kinetics (~10 min) to maximize brain fluorescence in the APP/PS1 mice, with intensity higher than that of probes **2**, **4**, **6** and CRANAD-58. Particularly, probe **9** hold great potential to discriminate 6-month-old (predominant soluble Aβ species) or 10-month-old (predominant insoluble Aβ species) APP/PS1 AD mice from age-matched WT mice, with signal intensity ratio of APP/PS1 AD mice to WT mice being ~1.5 and ~1.7 at 10 min respectively. Furthermore, dynamic fluorescence imaging of the skull-thinning APP/PS1 AD mouse on an upright fluorescent microscope demonstrated that probe **9** could immediately cross the BBB and selectively map the Aβ plaques in both brain parenchyma and cerebral angiopathic areas. Overall, our results demonstrated that our designed “off-on” NIR fluorescence probes could be efficient to detect both soluble and insoluble Aβ species *in vivo*, potential for applications in early diagnosis of AD and noninvasive evaluation of anti-AD drugs' efficacy *in vivo*.

## Supplementary Material

Supplementary methods, figures, tables, NMR and MS spectra.

Supplementary Movie 1 - APP-PS1 mouse.

Supplementary Movie 2 - WT mouse.

## Figures and Tables

**Table 1:** Response of probe **1**-**9** toward Aβ monomers, oligomers and aggregates in PBS buffer

Probes	Aβ monomers				Aβ oligomers				Aβ aggregates			
	*λ*_ex_ (nm)	*λ*_em_ (nm)	fold*^a^*	*K*_d_ (nM)	*λ*_ex_ (nm)	*λ*_em_ (nm)	fold*^a^*	*K*_d_ (nM)	*λ*_ex_ (nm)	*λ*_em_ (nm)	fold*^a^*	*K*_d_ (nM)
**1**	616	674	26	4.00 ± 0.34	615	672	29	35.66 ± 2.39	619	675	13	15.38 ± 1.07
**2**	620	693	34	8.64 ± 0.37	619	692	44	67.83 ± 4.70	620	700	15	28.02 ± 2.00
**3**	617	697	46	10.64 ± 1.03	618	695	53	36.16 ± 1.68	621	705	13	13.78 ± 1.06
**4**	623	702	51	31.66 ± 2.31	620	696	74	186.8 ± 22.69	623	708	20	39.93 ± 3.63
**5**	623	701	20	15.57 ± 1.74	619	694	30	72.57 ± 5.75	622	706	9	54.15 ± 4.15
**6**	593	707	15	3.01 ± 0.41	591	702	22	25.62 ± 1.54	605	705	9	13.51 ± 0.73
**7**	618	700	36	64.20 ± 5.38	615	692	43	166.8 ± 19.91	610	718	6	142.1 ± 8.29
**8**	616	692	42	10.14 ± 0.74	620	690	71	118.6 ± 9.71	622	700	23	76.34 ± 5.65
**9**	617	690	28	11.16 ± 0.79	618	688	35	36.59 ± 2.69	620	697	10	14.57 ± 1.27
CRANAD-58	580	674	27	4.84 ± 0.47	584	667	39	32.66 ± 1.99	588	675	12	13.49 ± 0.84

*^a^* Fluorescence activation ratio (fold) was measured by incubating 250 nM probe with 250 nM Aβ species.

## Abbreviations

AD: Alzheimer's disease
Aβ: amyloid-β
ThT: thioflavin T
MRI: magnetic resonance imaging
SPECT: single-photon emission-computed tomography
PET: positron emission computed tomography
NIR: near infrared
BODIPY: boron dipyrromethane
BBB: blood brain barrier
HOMO: highest occupied molecular orbital
LUMO: lowest unoccupied molecular orbital
APP/PS1: APPswe/PSEN1
WT: wild type
DMEM: dulbecco's modified eagle's medium
FBS: fetal bovine serum
MTT: methyl thiazolyl tetrazolium
HFIP: hexafluoroisopropanol
TEM: transmission electron microscopy
PTA: phosphotungstic acid
LOD: limit of detection
AChE: acetylcholinesterase
BuChE: Butyrylcholinesterase
GSH: glutathione
BSA: Bovine serum albumin
CAAs: cerebral amyloid angiopathies
