# Supplementary material for: Engineering of donor-acceptor-donor curcumin analogues as near-infrared fluorescent probes for in vivo imaging of amyloid-β species
Source: Theranostics. 2022 Apr 4;12(7):3178–95. doi: 10.7150/thno.68679 (PMC9065200; doi:10.7150/thno.68679)
Supplement: Supplementary file 1 — Supplementary methods, figures, tables, NMR and MS spectra. [file thnov12p3178s1.pdf]

Supporting Information

Engineering of donor-acceptor-donor curcumin analogues as near-infrared fluorescent probes for *in vivo* imaging of amyloid-β species

Daqing Fang[1,3#], Xidan Wen[2#], Yuqi Wang[2], Yidan Sun[2], Ruibing An[2], Yu Zhou[3], Deju Ye[2*], Hong Liu[1,3*]

1. Key Laboratory of Structure-Based Drug Design and Discovery, Ministry of Education, Shenyang Pharmaceutical University, Shenyang, 110016, China

2. State Key Laboratory of Analytical Chemistry for Life Sciences, Chemistry and Biomedicine Innovation Center (ChemBIC), School of Chemistry and Chemical Engineering, Nanjing University, Nanjing, 210023, China

3. State Key Laboratory of Drug Research, Shanghai Institute of Materia Medica, Chinese Academy of Sciences, Shanghai, 201203, China

# These authors have contributed equally to this work.

*Corresponding authors: Hong Liu, E-mail: hliu@simm.ac.cn; Deju Ye, E-mail: dejuye@nju.edu.cn.

Table of contents

1. Experimental Procedures S3

1.1 Instrument S3

1.2 Isothermal titration microcalorimetry (ITC) S3

1.3 Western Blotting S4

1.4 Measurement of blood circulation time S4

1.5 Biodistribution studies S5

1.6 Investigation of principal metabolic pathway S6

1.7 Synthesis and characterization S7

2. Supplementary tables S38

4. NMR and MS spectra S44

1. Experimental Procedures

1.1 Instrument

The ^{1}H and ^{13}C NMR spectra were acquired on a 400 MHz Bruker Avance III 400 spectrometer. High-performance liquid chromatography (HPLC) was carried out on Thermo Scientific Dionex Ultimate 3000 with CH_3CN/H_2O (1‰ CF_3COOH) as the eluents. Matrix-Assisted Laser Desorption/Ionization Time of Flight Mass Spectrometry (MALDI-TOF-MS) and high-resolution spectrometry (HRMS) analysis was conducted on AB SCIEX 4800 Plus MALDI TOF/TOFTM mass spectrometer and Bruker microTOF-Q111, respectively. The UV-Vis spectra were carried out on an Ocean Optics Maya 2000 Pro spectrometer. The fluorescence spectra were measured with a HORIBA Jobin Yvon Fluoromax-4 fluorometer. The calorimetric experiments were conducted on a MicroCal ITC200. TEM images were obtained on a JEM-1011 transmission electron microscope (JEOL, Ltd., Japan) with an accelerating voltage of 100 kV. MTT assay and the fluorescence intensity of probe **2** or probe **9** extracted from biological samples was performed on a microplate reader (Tcan). Fluorescent images of brain tissue slices were acquired on Olympus VS200 microscope. Confocal fluorescence images were obtained on Leica TCS SP8 confocal laser scanning microscope. In vivo fluorescence images were recorded with an IVIS Lumina XR III system, and fluorescence intensity was quantified by the region-of-interest measurement using Living image software (PerkinElmer).

1.2 Isothermal titration microcalorimetry (ITC)

The preparation of Aβ42 monomers was conducted according to previously reported approach (*Int J Biol Macromol*. 2021; 168: 611-619). The preparation of Aβ42 oligomers and aggregates was same as that in *in vitro* spectroscopic measurement.

ITC experiments were performed on MicroCal ITC200 calorimeter (GE). Probe **9** was placed in the sample cell (10 μM, containing 3% DMSO) and Aβ42 protein (Aβ42 monomers, oligomers and aggregates respectively) was loaded into syringe cell. All solution in its corresponding group were prepared in the same buffer and were degassed for 20 min before experiments. After equilibration at 25 ºC and an initial delay, 20 serial

injection (2 μL) were added into the sample cell from the syringe by using 1000 rpm stirring speed and 2 min intervals. In addition, the titrant was injected into the buffer solution in the sample cell to measure the heat of dilution. The value of the heat of dilution was subtracted from the titration data. Origin 7.0 software supplied with the instrument was used to analyze the ITC titration data.

1.3 Western Blotting

The protein was separated by SDS-PAGE (×1) and transferred to the nitrocellulose membrane by electrophoretic blotting (250 mA, 60 min). The membrane was sealed in 5% skimmed milk powder (dispersed in TBST buffer with 0.1% Tween 20) at room temperature for 1 h, incubated with 6E10 anti-Aβ primary antibody (1:2000 dilution, Biolegend) for 30 min at room temperature and stored at 4 ºC overnight. The next day, the membrane was incubated with the primary antibody at room temperature for 30 min. After washing with TBST 4 times, the membrane was incubated with horseradish peroxidase (HRP)-conjugated secondary antibody at room temperature for 1 h. Then, the membrane was washed with TBST 4 times. The secondary antibody on the membrane was visualized with ECL detection reagent using G:BOX chemiXR5. Image J was used to quantify the band intensity. RealBand 3-color Broad Range Protein Marker (Sangon Biotech)(4-250KDa) was used as a molecular weight marker.

The samples were prepared using the following procedure. A 10 μL HFIP (hexafluoroisopropanol) solution (25 μM) of native Aβ42 was added to a 1.5 mL eppendorf tube. After evaporating the organic solvent under vacuum, a 10 μL DMSO or DMSO stock solution of probe **9** (25, 125 and 250 μM) was added to the tube, followed by the addition of 30 μL of vitamin C solution in PBS (33.3 μM) and 10 μL of copper sulfate solution in PBS (pH 7.4) (12.5 μM). The resulting mixture was incubated at 37 °C for 4 h and then subjected to gel electrophoresis. For the CRANAD-58 group, the same operation was employed except that 10 μL DMSO solution of CRANAD-58 (250 μM) was added to the tube.

1.4 Measurement of blood circulation time

Probe **9** (1.0 mg/kg) was i.v. injected into the healthy BALB/c mice (5-6 weeks old). At 0, 15 min, 0.5, 1, 2, 4, 8, 12, and 24 h, bloods were collected from the venous sinus of mice (n = 3), and preserved into 1.5-mL EDTA coated Eppendorf tubes that are chilled on ice. 100 μL collected bloods at each time point were pipetted to Eppendorf tubes, and diluted with 400 μL PBS (pH = 7.4) and then homegenized vigorously (65 Hz, 60 s, adding 2 magnetic bead per tube). The homegenates (100 μL respectively) were successively denatured and extracted with equal volume methanol and acetonitrile respectively. The mixture was centrifuged at 14400 rpm for 5 min. The supernatant was transferred to an Eppendorf tube. The sediment was treated with equal volume PBS buffer (pH = 7.4), methanol and acetonitrile respectively as described above, and was repeated another time (totally 3 times). The supernatant in each time was merged and subjected to fluorescence assay on a microplate reader. Plot of the percentage of probe **9** remained in the blood versus injection time to determine the blood circulation half-time ($t_{1/2}$).

1.5 Biodistribution studies

Heathy female mice were i.v. injected with probe **9** (1.0 mg/kg). After 10, 60, and 240 min (n = 3), the mice were sacrificed, and major organs including heart, liver, spleen, lung, kidneys, intestines, stomach, brain and muscle were resected and then cut into small pieces and weighted. Each organ was homogenized with PBS beffer (pH = 7.4) (5.0 mL/g) vigorously (65 Hz, 240 s, adding 3-4 magnetic bead per tube). The homegenate (100 μL respectively) were successively denatured and extracted with equal volume methanol and acetonitrile respectively. The mixture was centrifuged at 14400 rpm for 5 min. The supernatant was transferred to an Eppendorf tube. The sediment was treated with equal volume PBS buffer (pH = 7.4), methanol and acetonitrile respectively as described above, and was repeated another time (totally 3 times). The supernatant in each time was merged and subjected to fluorescence assay on a microplate reader. The contents of probe **9** in each sample was deduced from the standard curve of probe **9**, divided by the injection dose and then normalized to the weight of each organ to obtain the value of %ID/g tissue.

1.6 Investigation of principal metabolic pathway

The healthy BALB/c mice were i.v. injected with probe **9** (1.0 mg/kg) and placed in metabolic cages for 72 h. Feces and urine were collected at the period of 0-4 h, 4-12 h, 12-24 h, 24-48 h and 48-72 h after injection. The urine was diluted with PBS buffer (pH = 7.4) 5 times for the following denature and extration. The feces was homegenized with PBS beffer (pH = 7.4) (5.0 mL/g) vigorously (65 Hz, 240 s, adding 3 magnetic bead per tube). The diluted urine and feces homegenate (100 μL respectively) were successively denatured and extracted with equal volume methanol and acetonitrile respectively. The mixture was centrifuged at 14400 rpm for 5 min. The supernatant was transferred to an Eppendorf tube. The sediment was treated with equal volume PBS buffer (pH = 7.4), methanol and acetonitrile respectively as described above, and was repeated another time (totally 3 times). The supernatant in each time was merged and subjected to fluorescence assay on a microplate reader. The contents of probe **9** in each sample was deduced from the standard curve of probe **9**, divided by the injection dose to obtain the value of %ID.

1.7 Synthesis and characterization

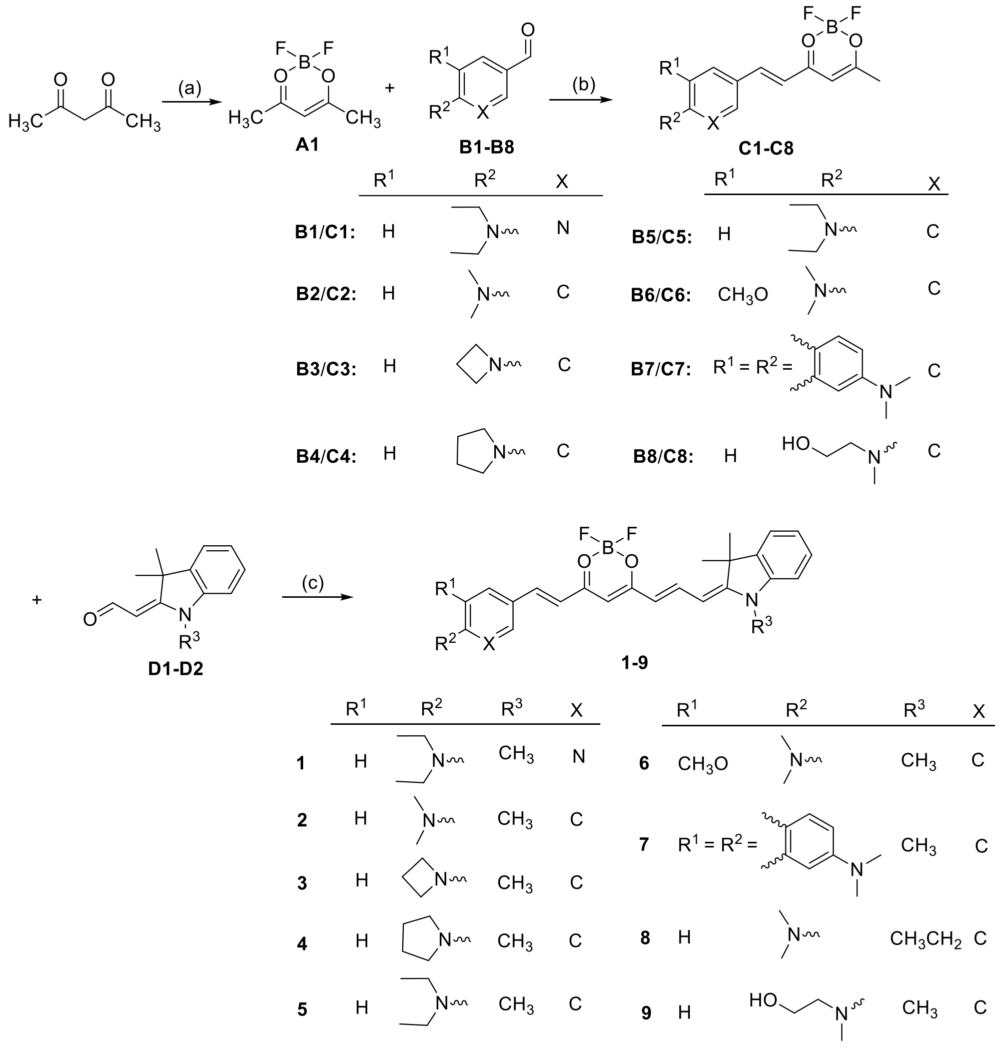

Scheme S1. Synthesis of curcumin derivatives. Reaction conditions: (a) $BF_3{\cdot}Et_2O$, Toluene, 60 °C, 2 h, 61%; (b) AcOH, tetrahydroisoquinoline, acetonitrile, 60 °C, overnight; (c) Ac_2O, 60 °C, overnight.

Synthesis of 2,2-Difluoro-1,3-dioxaboryl-pentadione (A1): $BF_3{\cdot}Et_2O$ (1.26 g, 9.99 mmol) was slowly added to a dry toluene solution (2.0 ml) containing acetylacetone (1g, 9.99 mmol), and the mixture was stirred at 60 °C for 2 h. Then, the mixture was evaporated and purified by flash chromatography on silica gel to give the desired product as a pale yellow solid. Yield: 899 mg (61%). ^{1}H NMR (400 MHz, $CDCl_3$) δ 5.98 (s, 1H), 2.29 (s, 6H). ^{13}C NMR (101 MHz, $CDCl_3$) δ 192.55, 102.04, 24.27. ^{19}F NMR (377 MHz, $CDCl_3$) δ -138.26, -138.33. HRMS (ESI): calcd. for $C_5H_8BF_2O_2$ $[M+H]^+$ 149.0580; found 149.0241.

*Synthesis of **C1***: **A1** (150 mg, 1.014 mmol) was dissolved in acetonitrile (2.0 mL), followed by addition of acetic acid (0.1 mL), tetrahydroisoquinoline (0.02 mL, 0.15 mmol) and **B1** (181 mg, 1.014 mmol). The resulting solution was stirred at 60 ℃ overnight. A black residue was obtained after removing the solvent, which was further purified with flash column chromatography to give a red powder. Yield: 109 mg (35%). ^{1}H NMR (400 MHz, $CDCl_3$) δ 8.35 (d, J = 2.4 Hz, 1H), 8.00 (d, J = 15.3 Hz, 1H), 7.67 (dd, J = 9.2, 2.4 Hz, 1H), 6.52 (d, J = 9.2 Hz, 1H), 6.35 (d, J = 15.3 Hz, 1H), 5.89 (s, 1H), 3.60 (q, J = 7.1 Hz, 4H), 2.27 (s, 3H), 1.23 (t, J = 7.1 Hz, 6H). ^{13}C NMR (101 MHz, $CDCl_3$) δ 188.19, 180.72, 159.10, 153.83, 147.35, 135.94, 117.93, 113.59, 106.37, 100.62, 43.32, 24.16, 13.05. MALDI-TOF-MS: calcd. for $C_{15}H_{20}BF_2N_2O_2$ $[M+H]^+$ 309.1580; found 309.1298.

*Synthesis of **C2***: **A1** (0.15g, 1.0 mmol) were dissolved in acetonitrile (2.0 mL), followed by the additions of acetic acid (0.1 mL), tetrahydroisoquinoline (0.02 mL, 0.15 mmol), and **B2** (0.15g, 1.0 mmol). The resulting solution was stirred at 60 °C overnight. A black residue was obtained after removing the solvent and further purified with flash column chromatography to give a red powder. Yield: 58.7 mg (21%). ^{1}H NMR (400 MHz, $CDCl_3$) δ 8.03 (d, J = 15.2 Hz, 1H), 7.50 (d, J = 8.9 Hz, 2H), 6.68 (d, J = 8.9 Hz, 2H), 6.38 (d, J = 15.2 Hz, 1H), 5.87 (s, 1H), 3.09 (s, 6H), 2.25 (s, 3H). ^{13}C NMR (101 MHz, $CDCl_3$) δ 187.18, 180.81, 153.36, 150.15, 132.22, 121.82, 113.30, 112.07, 100.54, 40.25, 29.83. HRMS(ESI): calcd. for $C_{14}H_{17}BF_2NO_2$ $[M+H]^+$ 280.1315; found 280.1308.

*Synthesis of **C3***: **A1** (150 mg, 1.014 mmol) were dissolved in acetonitrile (2.0 mL), followed by the additions of acetic acid (0.1 mL), tetrahydroisoquinoline (0.02 mL, 0.15 mmol), and **B3** (163 mg, 1.014 mmol). The resulting solution was stirred at 60 ℃ overnight. A black residue was obtained after removing the solvent and further purified with flash column chromatography to give a red powder. Yield: 120 mg (41%). ^{1}H NMR (400 MHz, $CDCl_3$) δ 8.02 (d, J = 15.2 Hz, 1H), 7.47 (d, J = 8.7 Hz, 2H), 6.41 – 6.32 (m, 3H), 5.86 (s, 1H), 4.04 (t, J = 7.4 Hz, 4H), 2.45 (quint, J = 7.4 Hz, 2H), 2.25 (s, 3H). ^{13}C NMR (101 MHz, $CDCl_3$) δ 187.23, 180.81, 153.99, 150.34, 132.12, 122.27,

113.25, 110.69, 100.54, 51.56, 24.05, 16.52. HRMS(ESI): calcd. for $C_{15}H_{17}BF_2NO_2$ $[M+H]^+$ 292.1315; found 292.1314.

*Synthesis of **C4***: **A1** (85 mg, 0.57 mmol) were dissolved in acetonitrile (2.0 mL), followed by the additions of acetic acid (0.1 mL), tetrahydroisoquinoline (0.02 mL, 0.15 mmol), and **B4** (100 mg, 0.57 mmol). The resulting solution was stirred at 60 ℃ overnight. A black residue was obtained after removing the solvent and further purified with flash column chromatography to give a red powder. Yield: 79.7 mg (45%). ^1H NMR (400 MHz, $CDCl_3$) δ 8.04 (d, J = 15.2 Hz, 1H), 7.50 (d, J = 8.8 Hz, 2H), 6.55 (d, J = 8.8 Hz, 2H), 6.35 (d, J = 15.2 Hz, 1H), 5.85 (s, 1H), 3.42-3.38 (m, 4H), 2.24 (s, 3H), 2.09 – 2.02 (m, 4H). ^{13}C NMR (101 MHz, $CDCl_3$) δ 186.55, 180.72, 151.12, 150.54, 132.51, 121.46, 112.54, 112.33, 100.42, 47.88, 29.84, 25.54, 23.99. HRMS(ESI): calcd. for $C_{16}H_{19}BF_2NO_2$ $[M+H]^+$ 306.1471; found 306.1486. HRMS(ESI): calcd. for $C_{16}H_{18}BF_2NNaO_2$ $[M+Na]^+$ 328.1291; found 328.1307.

*Synthesis of **C5***: **A1** (100 mg, 0.67 mmol) were dissolved in acetonitrile (2.0 mL), followed by the additions of acetic acid (0.1 mL), tetrahydroisoquinoline (0.02 mL, 0.15 mmol), and **B5** (119 mg, 0.67 mmol). The resulting solution was stirred at 60 ℃ overnight. A black residue was obtained after removing the solvent and further purified with flash column chromatography to give a red powder. Yield: 110 mg (49%). ^1H NMR (400 MHz, $CDCl_3$) δ 8.02 (d, J = 15.2 Hz, 1H), 7.48 (d, J = 9.0 Hz, 2H), 6.65 (d, J = 9.0 Hz, 2H), 6.35 (d, J = 15.2 Hz, 1H), 5.86 (s, 1H), 3.44 (q, J = 7.1 Hz, 4H), 2.24 (s, 3H), 1.22 (t, J = 7.1 Hz, 6H). ^{13}C NMR (101 MHz, $CDCl_3$) δ 186.55, 180.74, 151.40, 150.21, 132.66, 121.21, 112.58, 111.66, 100.43, 44.89, 23.97, 12.71. HRMS(ESI): calcd. for $C_{16}H_{21}BF_2NO_2$ $[M+H]^+$ 308.1628; found 308.1635. HRMS(ESI): calcd. for $C_{16}H_{20}BF_2NNaO_2$ $[M+Na]^+$ 330.1447; found 330.1455.

*Synthesis of **C6***: **A1** (87 mg, 0.558 mmol) were dissolved in acetonitrile (2.0 mL), followed by the additions of acetic acid (0.1 mL), tetrahydroisoquinoline (0.02 mL, 0.15 mmol), and **B6** (100 mg, 0.558 mmol). The resulting solution was stirred at 60 ℃ overnight. A black residue was obtained after removing the solvent and further purified with flash column chromatography to give a red powder. Yield: 65 mg (38%). ^1H NMR

(400 MHz, $CDCl_3$) δ 8.01 (d, J = 15.4 Hz, 1H), 7.19 (dd, J = 8.3, 1.9 Hz, 1H), 7.04 (d, J = 1.8 Hz, 1H), 6.83 (d, J = 8.3 Hz, 1H), 6.47 (d, J = 15.4 Hz, 1H), 5.95 (s, 1H), 3.91 (s, 3H), 2.94 (s, 6H), 2.29 (s, 3H). ^{13}C NMR (101 MHz, $CDCl_3$) δ 189.15, 180.79, 151.49, 149.39, 146.96, 126.53, 125.34, 117.28, 116.01, 111.11, 100.98, 55.71, 42.79, 24.24. HRMS (ESI): calcd. for $C_{15}H_{19}BF_2NO_3$ $[M+H]^+$ 310.1421; found 310.1415.

*Synthesis of **C7***: **A1** (50 mg, 0.335 mmol) were dissolved in acetonitrile (2.0 mL), followed by the additions of acetic acid (0.1 mL), tetrahydroisoquinoline (0.02 mL, 0.15 mmol), and **B7** (67 mg, 0.335 mmol). The resulting solution was stirred at 60 ℃ overnight. A black residue was obtained after removing the solvent and further purified with flash column chromatography to give a dark red powder. Yield: 28 mg (25%). ^1H NMR (400 MHz, $CDCl_3$) δ 8.18 (d, J = 15.4 Hz, 1H), 7.86 (s, 1H), 7.73 (d, J = 9.1 Hz, 1H), 7.60 (d, J = 8.7 Hz, 1H), 7.57 (d, J = 8.7 Hz, 1H), 7.14 (dd, J = 9.1, 2.3 Hz, 1H), 6.85 (d, J = 1.8 Hz, 1H), 6.61 (d, J = 15.4 Hz, 1H), 5.96 (s, 1H), 3.12 (s, 6H), 2.31 (s, 3H). ^{13}C NMR (101 MHz, $CDCl_3$) δ 189.42, 180.89, 150.47, 149.94, 137.51, 133.59, 130.69, 127.40, 127.25, 125.92, 124.23, 116.82, 116.39, 105.77, 101.15, 40.53, 29.84. HRMS(ESI): calcd. for $C_{18}H_{19}BF_2NO_2$ $[M+H]^+$ 330.1471; found 330.1468.

*Synthesis of **C8***: **A1** (30 mg, 0.203 mmol) were dissolved in acetonitrile (2.0 mL), followed by the additions of acetic acid (0.1 mL), tetrahydroisoquinoline (0.02 mL, 0.15 mmol), and **B8** (36 mg, 0.203 mmol). The resulting solution was stirred at 60 ℃ overnight. A black residue was obtained after removing the solvent and further purified with flash column chromatography to give a dark red powder. Yield: 14 mg (22%). ^1H NMR (400 MHz, $CDCl_3$) δ 8.00 (d, J = 15.3 Hz, 1H), 7.48 (d, J = 8.9 Hz, 2H), 6.74 (d, J = 8.9 Hz, 2H), 6.37 (d, J = 15.2 Hz, 1H), 5.87 (s, 1H), 3.86 (t, J = 5.7 Hz, 2H), 3.61 (t, J = 5.7 Hz, 2H), 3.12 (s, 3H), 2.25 (s, 3H), 1.36 – 1.13 (m, 1H). ^{13}C NMR (101 MHz, $CDCl_3$) δ 187.41, 180.81, 152.90, 149.94, 132.28, 122.15, 113.55, 112.26, 100.61, 60.29, 54.47, 39.29, 24.05. HRMS(ESI): calcd. for $C_{15}H_{19}BF_2NO_3$ $[M+H]^+$ 310.1421; found 310.1422. HRMS(ESI): calcd. for $C_{15}H_{18}BF_2NNaO_3$ $[M+Na]^+$ 332.1240; found 332.1242.

*Synthesis of probe **1***: **C1** (20 mg, 0.065 mmol) and Fischer′s aldehyde (**D1**) (13 mg, 0.065 mmol) were dissolved in acetic anhydride (2.0 mL). After stirred at room temperature for 10 min, the mixture was transferred to stirred at 60 ℃ overnight. The acetic anhydride was removed under vacuum and the residues was purified by flash chromatography to give dark powder. Yield: 11 mg (34%). ^{1}H NMR (400 MHz, $CDCl_3$) *δ* 8.33 (m, 2H), 7.74 (d, *J* = 15.4 Hz, 1H), 7.63 (d, *J* = 8.5 Hz, 1H), 7.36 (m, 2H), 7.08 (d, *J* = 6.9 Hz, 1H), 6.86 (d, *J* = 7.5 Hz, 1H), 6.49 (d, *J* = 8.7 Hz, 1H), 6.38 (d, *J* = 15.3 Hz, 1H), 5.81 (d, *J* = 13.4 Hz, 1H), 5.68 (s, 1H), 5.66 (d, *J* = 12.2 Hz, 1H), 3.56 (d, *J* = 6.5 Hz, 4H), 3.32 (s, 3H), 1.64 (s, 6H), 1.22 (d, *J* = 6.4 Hz, 6H). ^{13}C NMR (101 MHz, $CDCl_3$) *δ* 177.42, 173.67, 169.64, 158.08, 151.42, 145.52, 143.47, 140.40, 140.22, 135.53, 128.25, 123.08, 122.13, 118.88, 116.53, 113.59, 108.43, 106.16, 100.64, 97.65, 48.05, 43.13, 30.00, 28.77, 28.49. HRMS(ESI): calcd. for $C_{28}H_{33}BF_2N_3O_2$ $[M+H]^+$ 492.2628; found 492.2629.

*Synthesis of probe **2***: **C2** (50 mg, 0.179 mmol) and Fischer′s aldehyde (**D1**) (39.7 mg, 0.197 mmol) were dissolved in acetic anhydride (2.0 mL). After stirred at room temperature for 10 min, the mixture was transferred to stirred at 60 ℃ overnight. The acetic anhydride was removed under vacuum and the residues was purified by flash chromatography to give dark powder. Yield: 46 mg (55%). ^{1}H NMR (400 MHz, $CDCl_3$) *δ* 8.32 (t, *J* = 13.4 Hz, 1H), 7.81 (d, *J* = 15.4 Hz, 1H), 7.46 (d, *J* = 8.8 Hz, 2H), 7.28 (d, *J* = 6.3 Hz, 1H), 7.25 (s, 1H), 7.06 (t, *J* = 7.4 Hz, 1H), 6.84 (d, *J* = 8.0 Hz, 1H), 6.68 (d, *J* = 8.6 Hz, 2H), 6.41 (d, *J* = 15.4 Hz, 1H), 5.81 (d, *J* = 13.6 Hz, 1H), 5.68 (s, 1H), 5.65 (d, *J* = 13.2 Hz, 1H), 3.31 (s, 3H), 3.04 (s, 6H), 1.65 (s, 6H). ^{13}C NMR (101 MHz, $CDCl_3$) *δ* 177.22, 174.36, 169.17, 152.07, 145.01, 143.66, 143.58, 140.19, 130.70, 128.22, 122.90, 122.12, 116.23, 113.85, 112.14, 108.29, 100.56, 97.48, 53.57, 47.94, 40.33, 29.94, 28.79. HRMS(ESI): calcd. for $C_{27}H_{30}BF_2N_2O_2$ $[M+H]^+$ 463.2363; found 463.2361.

*Synthesis of probe **3***: **C3** (32 mg, 0.110 mmol) and Fischer′s aldehyde (**D1**) (22 mg, 0.110 mmol) were dissolved in acetic anhydride (2.0 mL). After stirred at room temperature for 10 min, the mixture was transferred to stirred at 60 ℃ overnight. The

acetic anhydride was removed under vacuum and the residues was purified by flash chromatography to give dark powder. Yield: 28 mg (54%). ^{1}H NMR (400 MHz, $CDCl_3$) δ 8.32 (t, J = 13.4 Hz, 1H), 7.80 (d, J = 15.4 Hz, 1H), 7.42 (d, J = 8.6 Hz, 2H), 7.30 – 7.27 (m, 1H), 7.25 (s, 1H), 7.06 (t, J = 7.7 Hz, 1H), 6.85 (d, J = 7.8 Hz, 1H), 6.40 (d, J = 15.84 Hz, 1H), 6.37 (d, J = 8.9 Hz, 2H), 5.81 (d, J = 13.5 Hz, 1H), 5.68 (s, 1H), 5.65 (d, J = 13.2 Hz, 1H), 3.98 (t, J = 7.3 Hz, 4H), 3.32 (s, 3H), 2.48-2.37 (m, 2H), 1.65 (s, 6H). ^{13}C NMR (101 MHz, $CDCl_3$) δ 177.30, 174.35, 169.22, 153.15, 145.09, 143.83, 143.58, 140.21, 130.55, 128.22, 123.83, 122.92, 122.14, 116.31, 113.83, 110.97, 108.28, 100.58, 97.47, 51.90, 47.96, 29.95, 29.84, 28.80. HRMS(ESI): calcd. for $C_{28}H_{30}BF_2N_2O_2$ $[M+H]^+$ 475.2363; found 475.2367.

Synthesis of probe ***4***: **C4** (50 mg, 0.163 mmol) and Fischer′s aldehyde (**D1**) (32.9 mg, 0.163 mmol) were dissolved in acetic anhydride (2.0 mL). After stirred at room temperature for 10 min, the mixture was transferred to stirred at 60 ℃ overnight. The acetic anhydride was removed under vacuum and the residues was purified by flash chromatography to give dark powder. Yield: 21 mg (35%). ^{1}H NMR (400 MHz, $CDCl_3$) δ 8.28 (t, J = 13.4 Hz, 1H), 7.80 (d, J = 15.3 Hz, 1H), 7.42 (d, J = 8.7 Hz, 2H), 7.26 (s, 1H), 7.23 (s, 1H), 7.03 (t, J = 7.4 Hz, 1H), 6.81 (d, J = 7.9 Hz, 1H), 6.51 (d, J = 8.7 Hz, 2H), 6.36 (d, J = 15.3 Hz, 1H), 5.79 (d, J = 13.6 Hz, 1H), 5.65 (s, 1H), 5.62 (d, J = 13.2 Hz, 1H), 3.33 (t, J = 6.5 Hz, 4H), 3.28 (s, 3H), 2.09 – 1.96 (m, 4H), 1.62 (s, 6H). ^{13}C NMR (101 MHz, $CDCl_3$) δ 176.98, 174.58, 168.85, 149.78, 144.63, 144.19, 143.63, 140.17, 130.99, 128.19, 122.77, 122.57, 122.10, 115.41, 114.01, 112.10, 108.20, 100.44, 97.39, 47.87, 47.80, 29.90, 28.79, 25.57. HRMS(ESI): calcd. for $C_{29}H_{32}BF_2N_2O_2$ $[M+H]^+$ 489.2519; found 489.2519.

*Synthesis of probe **5***: **C5** (50 mg, 0.162 mmol) and Fischer′s aldehyde (**D1**) (32.7 mg, 0.162 mmol) were dissolved in acetic anhydride (2.0 mL). After stirred at room temperature for 10 min, the mixture was transferred to stirred at 60 ℃ overnight. The acetic anhydride was removed under vacuum and the residues was purified by flash chromatography to give dark powder. Yield: 30 mg (38%). ^{1}H NMR (400 MHz, $CDCl_3$) δ 8.31 (t, J = 13.4 Hz, 1H), 7.80 (d, J = 15.3 Hz, 1H), 7.43 (d, J = 8.6 Hz, 2H), 7.28 (d,

J = 2.9 Hz, 1H), 7.25 (s, 1H), 7.05 (t, *J* = 7.4 Hz, 1H), 6.84 (d, *J* = 8.1 Hz, 1H), 6.63 (d, *J* = 8.0 Hz, 2H), 6.38 (d, *J* = 15.3 Hz, 1H), 5.81 (d, *J* = 13.6 Hz, 1H), 5.68 (s, 1H), 5.64 (d, *J* = 13.1 Hz, 1H), 3.41 (q, *J* = 6.9 Hz, 4H), 3.31 (s, 3H), 1.64 (s, 6H), 1.41 (d, *J* = 13.6 Hz, 1H), 1.20 (t, *J* = 7.0 Hz, 6H). ^{13}C NMR (101 MHz, $CDCl_3$) δ 177.03, 174.63, 168.89, 149.95, 144.67, 143.96, 143.62, 140.17, 131.12, 128.34, 128.20, 122.80, 122.28, 122.11, 115.42, 113.97, 111.52, 108.22, 100.45, 97.40, 47.88, 44.71, 29.91, 28.79. HRMS(ESI): calcd. for $C_{29}H_{34}BF_2N_2O_2$ $[M+H]^+$ 491.2676; found 491.2677.

*Synthesis of probe **6***: **C6** (30 mg, 0.097 mmol) and Fischer′s aldehyde (**D1**) (20 mg, 0.097 mmol) were dissolved in acetic anhydride (2.0 mL). After stirred at room temperature for 10 min, the mixture was transferred to stirred at 60 ℃ overnight. The acetic anhydride was removed under vacuum and the residues was purified by flash chromatography to give dark powder. Yield: 17 mg (36%). ^{1}H NMR (400 MHz, $CDCl_3$) δ 8.37 (t, *J* = 13.4 Hz, 1H), 7.78 (d, *J* = 15.5 Hz, 1H), 7.29 (m, 2H), 7.10 (m, 2H), 7.02 (s, 1H), 6.88 (d, *J* = 8.0 Hz, 2H), 6.49 (d, *J* = 15.5 Hz, 1H), 5.82 (d, *J* = 13.4 Hz, 1H), 5.73 (s, 1H), 5.70 (d, *J* = 13.4 Hz, 1H), 3.91 (s, 3H), 3.34 (s, 3H), 2.88 (s, 6H), 1.65 (s, 6H). ^{13}C NMR (101 MHz, $CDCl_3$) δ 177.74, 173.22, 170.19, 151.93, 146.15, 143.38, 142.44, 140.28, 128.30, 123.30, 123.08, 122.16, 119.08, 117.90, 113.36, 110.44, 108.58, 101.12, 97.85, 55.61, 48.18, 43.05, 30.07, 29.82, 28.77. HRMS(ESI): calcd. for $C_{28}H_{32}BF_2N_2O_3$ $[M+H]^+$ 493.2469; found 493.2470.

*Synthesis of probe **7***: **C7** (44 mg, 0.134 mmol) and Fischer′s aldehyde (**D1**) (27 mg, 0.134 mmol) were dissolved in acetic anhydride (2.0 mL). After stirred at room temperature for 10 min, the mixture was transferred to stirred at 60 ℃ overnight. The acetic anhydride was removed under vacuum and the residues was purified by flash chromatography to give dark powder. Yield: 18 mg (26%). ^{1}H NMR (400 MHz, $CDCl_3$) δ 8.40 (t, *J* = 13.4 Hz, 1H), 7.95 (d, *J* = 15.5 Hz, 1H), 7.84 (s, 1H), 7.79 (d, *J* = 8.7 Hz, 1H), 7.67 (d, *J* = 8.1 Hz, 1H), 7.63 (d, *J* = 8.2 Hz, 1H), 7.34 – 7.27 (m, 2H), 7.10 (t, *J* = 7.4 Hz, 1H), 6.89 (d, *J* = 8.0 Hz, 1H), 6.67 (d, *J* = 15.5 Hz, 1H), 5.85 (d, *J* = 13.4 Hz, 1H), 5.76 (s, 1H), 5.71 (d, *J* = 13.3 Hz, 1H), 3.36 (s, 2H), 3.14 (s, 6H), 1.67 (s, 6H). ^{13}C NMR (101 MHz, $CDCl_3$) δ 177.24, 174.52, 168.38, 152.11, 144.97, 143.82, 143.07,

140.19, 130.74, 128.27, 122.93, 122.22, 116.17, 113.97, 112.11, 108.40, 100.55, 97.38, 82.86, 70.21, 48.03, 41.77, 40.31, 29.83, 28.90, 25.52, 16.24. HRMS(ESI): calcd. for $C_{31}H_{32}BF_2N_2O_2$ $[M+H]^+$ 513.2519; found 513.2517.

*Synthesis of probe **8***: **C1** (40 mg, 0.186 mmol) and *N*-ethyl-3,3-dimethyl-indoline (**D2**) (37 mg, 0.186 mmol) were dissolved in acetic anhydride (2.0 mL). After stirred at room temperature for 10 min, the mixture was transferred to stirred at 60 ℃ overnight. The acetic anhydride was removed under vacuum and the residues was purified by flash chromatography to give dark powder. Yield: 55 mg (62%).^{1}H NMR (400 MHz, $CDCl_3$) δ 8.34 (t, J = 13.4 Hz, 1H), 7.81 (d, J = 15.4 Hz, 1H), 7.46 (d, J = 8.6 Hz, 2H), 7.29 (d, J = 2.3 Hz, 1H), 7.27 (d, J = 2.3 Hz, 1H), 7.07 (t, J = 7.4 Hz, 1H), 6.85 (d, J = 8.0 Hz, 1H), 6.70 (d, J = 7.3 Hz, 2H), 6.42 (d, J = 15.4 Hz, 1H), 5.80 (d, J = 13.5 Hz, 1H), 5.68 (s, 1H), 5.678 (d, J = 13.2 Hz, 1H), 3.82 (q, J = 7.1 Hz, 2H), 3.05 (s, 6H), 1.64 (s, 6H), 1.32 (t, J = 7.2 Hz, 3H). ^{13}C NMR (101 MHz, $CDCl_3$) δ 177.19, 174.09, 168.31, 151.94, 145.34, 143.41, 142.61, 140.47, 130.65, 128.25, 122.96, 122.25, 116.65, 116.42, 113.45, 112.30, 108.31, 100.55, 97.07, 48.13, 40.43, 37.92, 28.77, 28.59. HRMS(ESI): calcd. for $C_{28}H_{32}BF_2N_2O_2$ $[M+H]^+$ 477.2519; found 477.2517.

*Synthesis of probe **9***: **C8** (35 mg, 0.113 mmol) and Fischer′s aldehyde (**D1**) (23 mg, 0.113 mmol) were dissolved in acetic anhydride (2.0 mL). After stirred at room temperature for 10 min, the mixture was transferred to stirred at 60 ℃ overnight. The acetic anhydride was removed under vacuum and the residues was purified by flash chromatography to give dark powder. Yield: 13 mg (23%). ^{1}H NMR (400 MHz, $CDCl_3$) δ 8.33 (t, J = 13.4 Hz, 1H), 7.79 (d, J = 15.4 Hz, 1H), 7.45 (d, J = 8.8 Hz, 2H), 7.28 (m, 1H), 7.25 (m, 1H), 7.07 (t, J = 7.4 Hz, 1H), 6.85 (d, J = 8.0 Hz, 1H), 6.71 (d, J = 8.8 Hz, 2H), 6.41 (d, J = 15.4 Hz, 1H), 5.81 (d, J = 13.5 Hz, 1H), 5.69 (s, 1H), 5.66 (d, J = 13.3 Hz, 1H), 4.26 (t, J = 5.9 Hz, 2H), 3.66 (t, J = 6.0 Hz, 2H), 3.32 (s, 3H), 3.05 (s, 3H), 1.64 (s, 6H). ^{13}C NMR (101 MHz, $CDCl_3$) δ 177.32, 174.13, 171.05, 169.37, 150.84, 145.24, 143.53, 143.24, 140.20, 130.72, 128.23, 123.67, 122.97, 122.12, 116.64, 113.73, 112.15, 108.35, 100.65, 97.55, 61.35, 50.89, 47.98, 38.90, 29.97, 28.78, 20.97. HRMS(ESI): calcd. for $C_{28}H_{31}BF_2N_2NaO_3$ $[M+Na]^+$ 515.2288; found 515.2288.

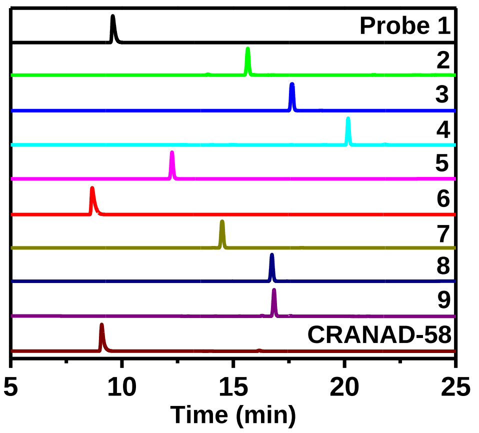

Figure S1. The normalized HPLC profiles of probe **1-9** and CRANAD-58. The spectra were recorded using a gradient of 50%-95% acetonitrile-water eluent. Both acetonitrile and water contained 1‰ TFA.

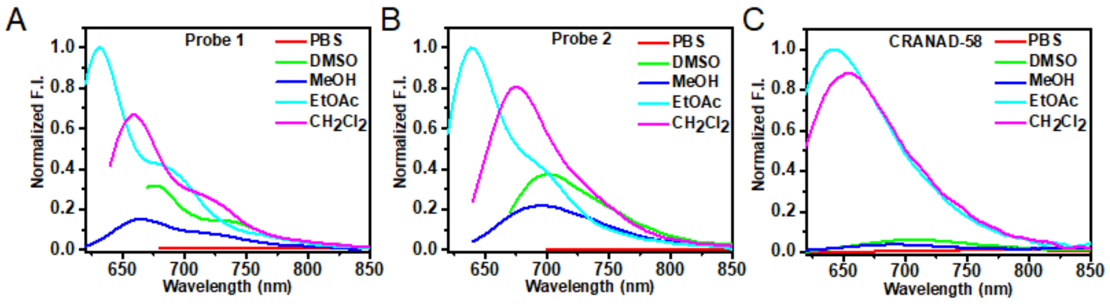

Figure S2. Fluorescence spectrum of 2.5 μM probe **1** (A), probe **2** (B) and CRANAD-58 (C) in different solutions. (Note: PBS buffer contained 10% DMSO).

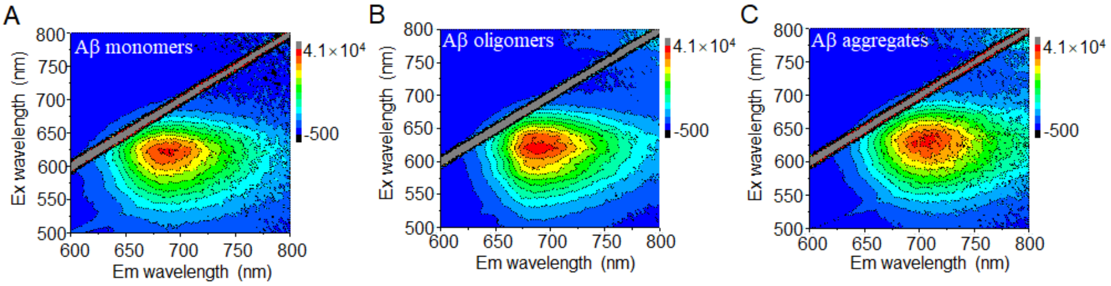

Figure S3. Contour map of probe **2** (250 nM) after interaction with 250 nM Aβ_{42} monomers (A), Aβ oligomers (B) and Aβ aggregates (C) in PBS buffer (pH = 7.4).

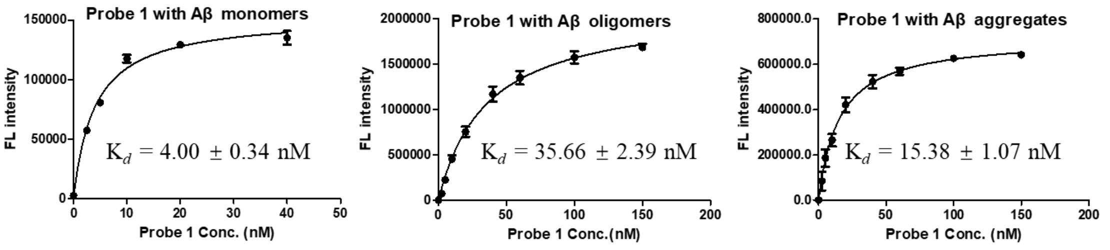

Figure S4. Binding constants (K_d) of probe **1** against 2.5 μM Aβ_{42} monomers, oligomers and aggregates, respectively.

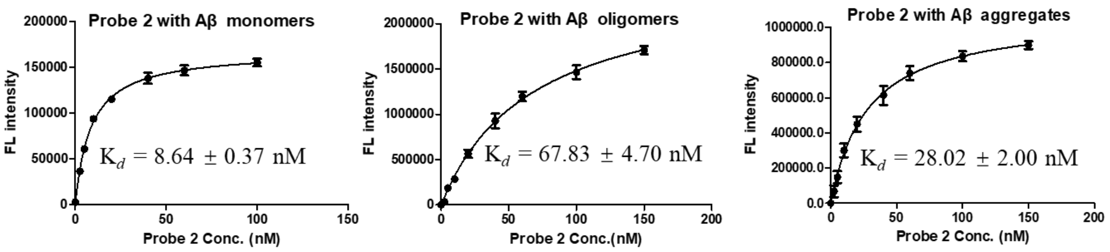

Figure S5. Binding constants (K_d) of probe **2** against 2.5 μM $A\beta_{42}$ monomers, oligomers and aggregates, respectively.

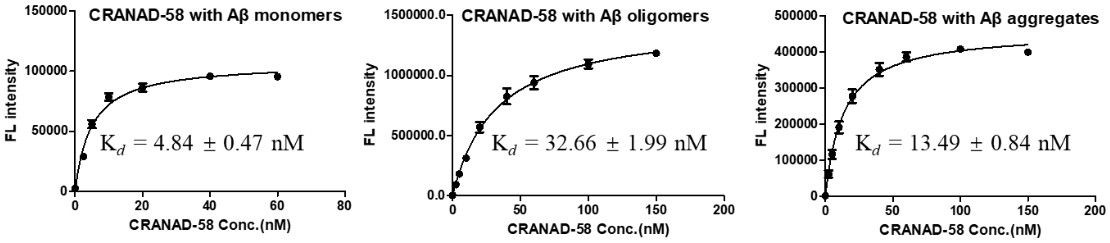

Figure S6. Binding constants (K_d) of CRANAD-58 against 2.5 μM $A\beta_{42}$ monomers, oligomers and aggregates, respectively.

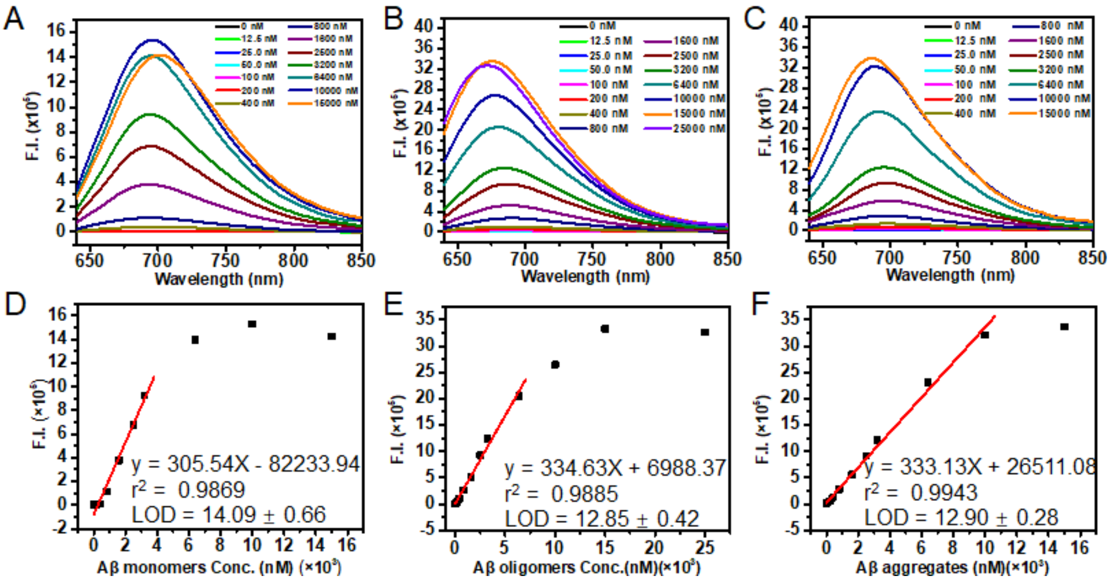

Figure S7. Determination of the sensitivity of probe **2** against $A\beta_{42}$ species. (A, B, C) Fluorescence spectrum of probe **2** (250 nM) incubating with different concentrations of (A) $A\beta_{42}$ monomers, (B) $A\beta_{42}$ oligomers and (C) $A\beta_{42}$ aggregates, respectively. (D, E, F) Linear fitting curves of mean fluorescence intensity versus the concentration of (D) $A\beta_{42}$ monomers, (E) $A\beta_{42}$ oligomers and (F) $A\beta_{42}$ aggregates. The LOD of probe **2** toward each $A\beta_{42}$ species could be calculated using 3σ/k. Values are mean ± SD, (n = 3).

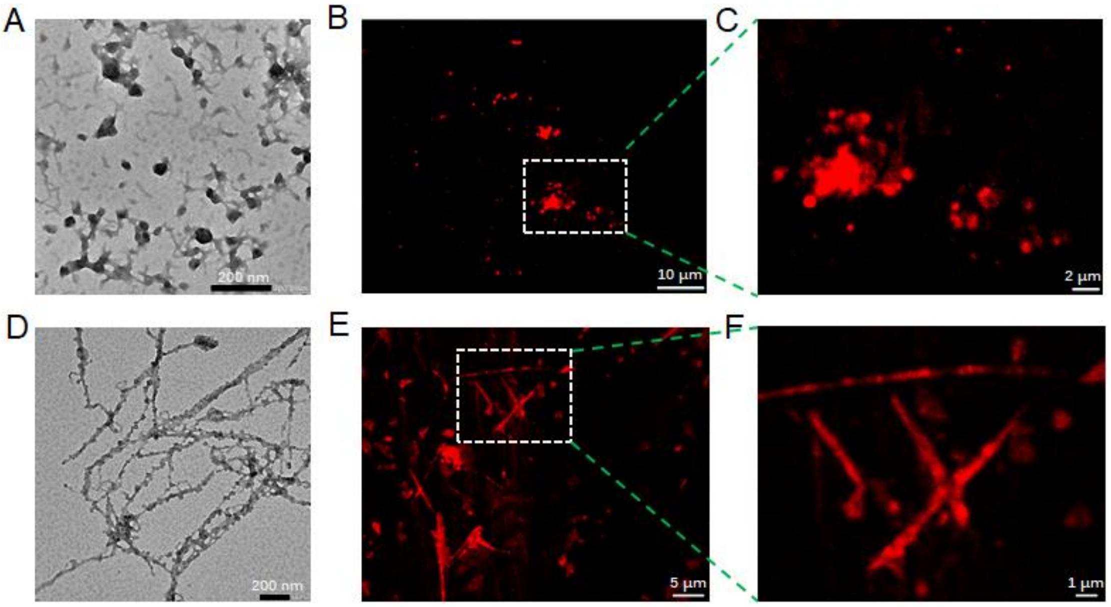

Figure S8. (A) TEM images of Aβ42 oligomers and (B, C) confocal fluorescence imaging of Aβ42 oligomers (25.0 μM) upon staining with probe **2** (2.5 μM). (D) TEM images of Aβ42 aggregates and (E, F) confocal fluorescence imaging of Aβ42 aggregates (25.0 μM) upon staining with probe **2** (2.5 μM). TEM images were acquired with negative staining with phosphotungstic acid (PTA).

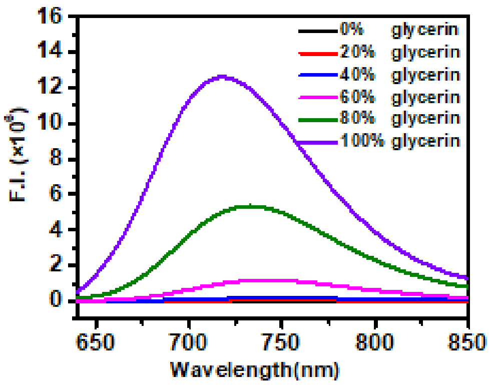

Figure S9. Fluorescence spectra of probe **2** (1.0 μM) in PBS buffer (pH = 7.4) (contained 2.0% DMSO) that contained varying amount of glycerin to increase the viscosity.

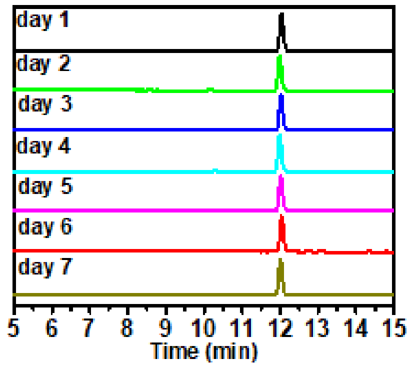

Figure S10. The HPLC traces of probe **2** (250 nM) after incubation in PBS buffer (pH = 7.4) at room temperature for 1-7 days. The HPLC analysis was run using a gradient of 65%-95% acetonitrile-water eluent. Both acetonitrile and water contained 1‰ TFA.

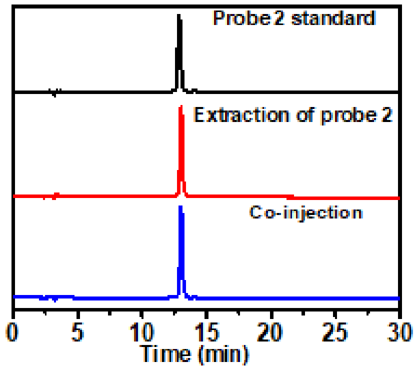

Figure S11. The HPLC profiles of probe **2** upon incubation with mouse serum at room temperature for 2 h. The spectra were recorded using a gradient of 60%-95% acetonitrile-water eluent. Both acetonitrile and water contained 1‰ TFA.

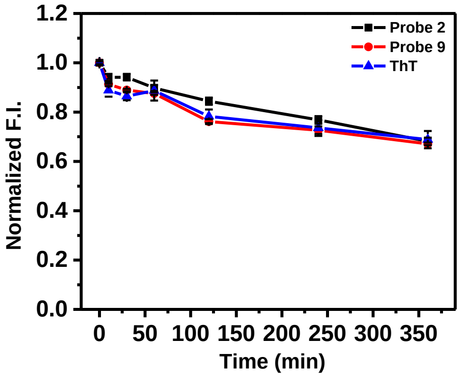

Figure S12. Photostability analysis of probe **2** (black), probe **9** (red) and ThT (blue) (all at 10 mM in DMSO) under lamplight ($\lambda > 400$ nm, 30 mW/cm^2) for different times. λ_{ex} = 620 nm, λ_{em} = 701 nm for probe **2**; λ_{ex} = 620 nm, λ_{em} = 693 nm for probe **9**; λ_{ex} = 416 nm, λ_{em} = 497 nm for ThT.

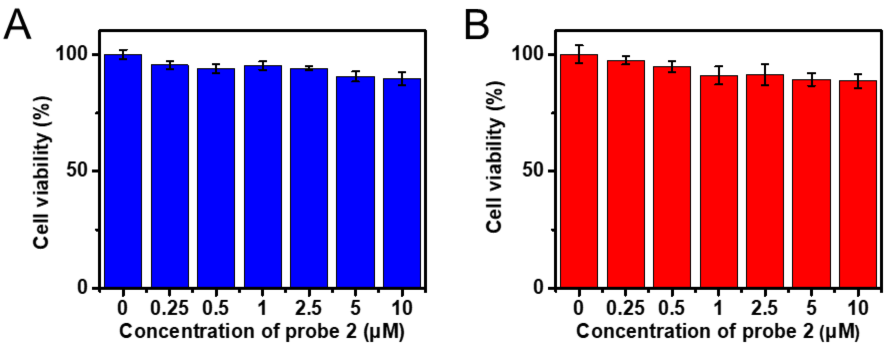

Figure S13. Cell viability of probe **2** towards U87MG cells (A) and PC-12 cells (B) at 0, 0.25, 0.5, 1.0, 2.5, 5.0 and 10.0 μM for 24 h respectively. Data were present as mean ± S.D. (n = 3).

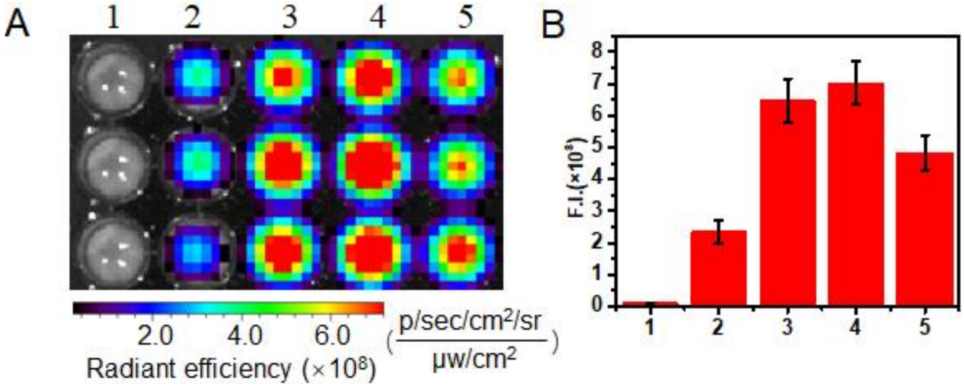

Figure S14. (A) Fluorescence images and (B) mean fluorescence intensity (F.I.) of brain homogenates (1), brain homogenates added with probe **2** (2), and brain homogenates added with probe **2** plus Aβ monomers (3), Aβ oligomers (4) or Aβ aggregates (5). The concentrations of probe **2** and each Aβ species were at 5 μM. Data were mean ± S.D. (n = 3).

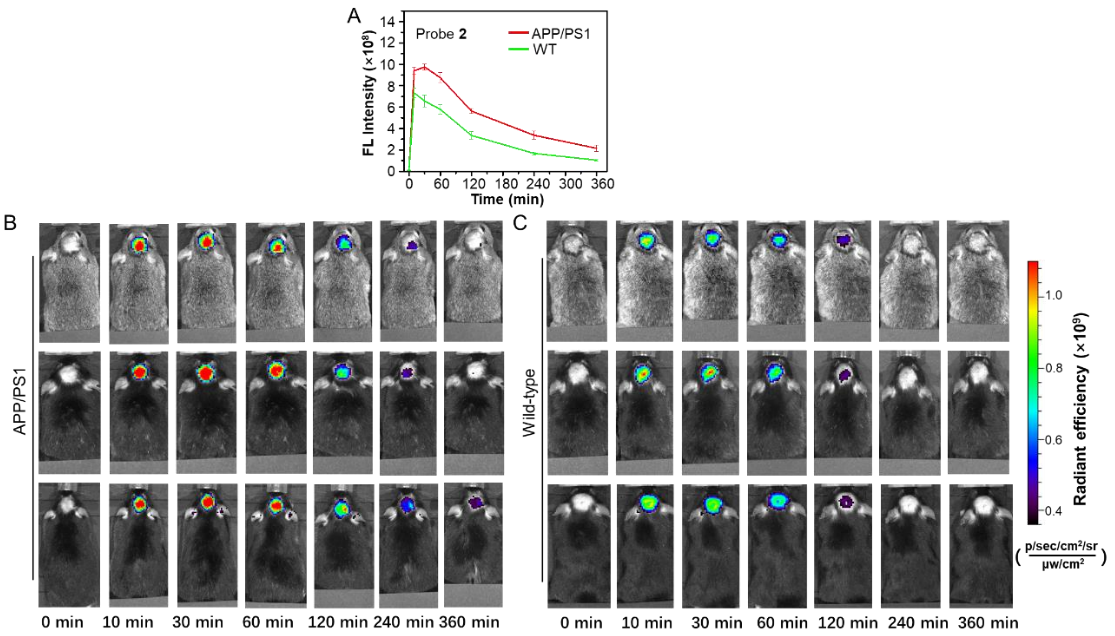

Figure S15. Quantitative analysis of the brain FL intensities of the APP/PS1 and WT mice at indicated time (A). Longitudinal FL images of APP/PS1 transgenic mice (B) and WT mice (C) (10-month old) following i.v. injection of probe **2** (1.0 mg/kg) at 0, 10, 30, 60, 120, 240 and 360 min.

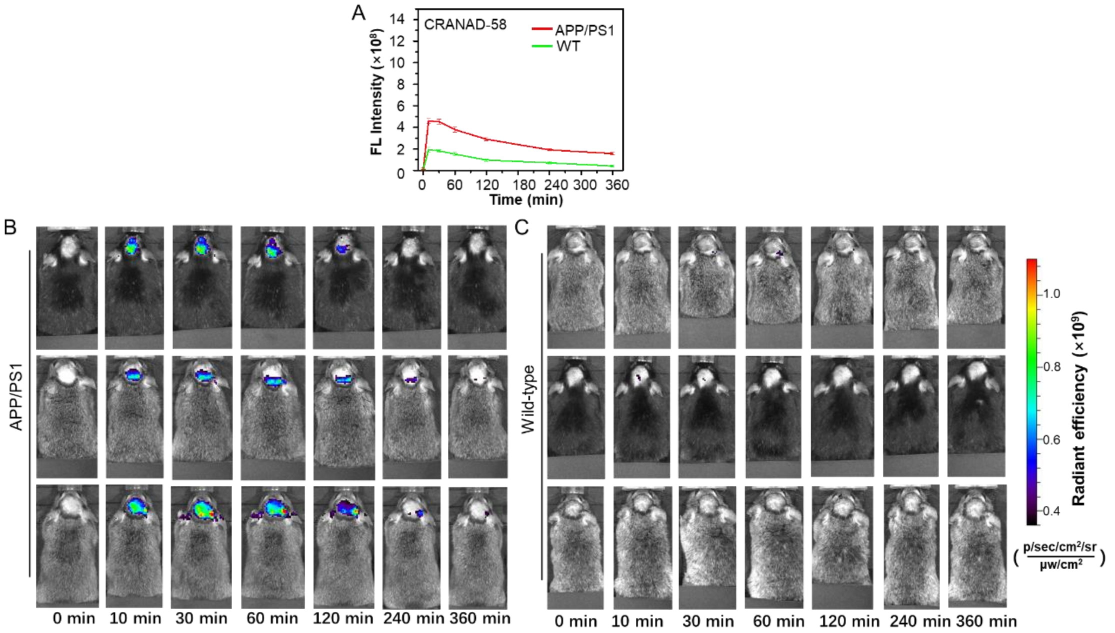

Figure S16. Quantitative analysis of the brain FL intensities of the APP/PS1 and WT mice at indicated time (A). Longitudinal FL images of APP/PS1 transgenic mice (B) and WT mice (C) (10-month old) following i.v. injection of CRANAD-58 (1.0 mg/kg) at 0, 10, 30, 60, 120, 240 and 360 min.

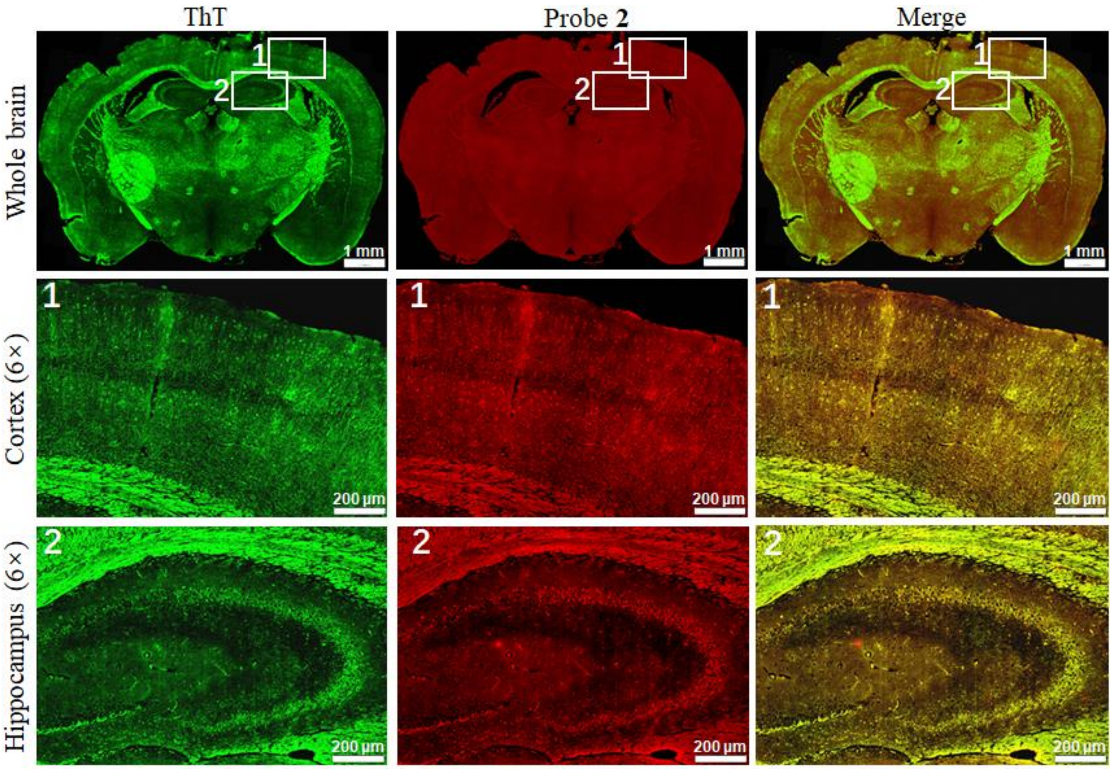

Figure S17. Ex vivo fluorescence imaging of cerebrum tissue slice resected from a 14-month-old WT mouse at 30 min after i.v. injection of probe **2** (1 mg/kg). Boxes 1 and 2 indicated enlarged cerebral cortex and hippocampus, respectively. The resected cerebrum tissue slice was co-stained with ThT.

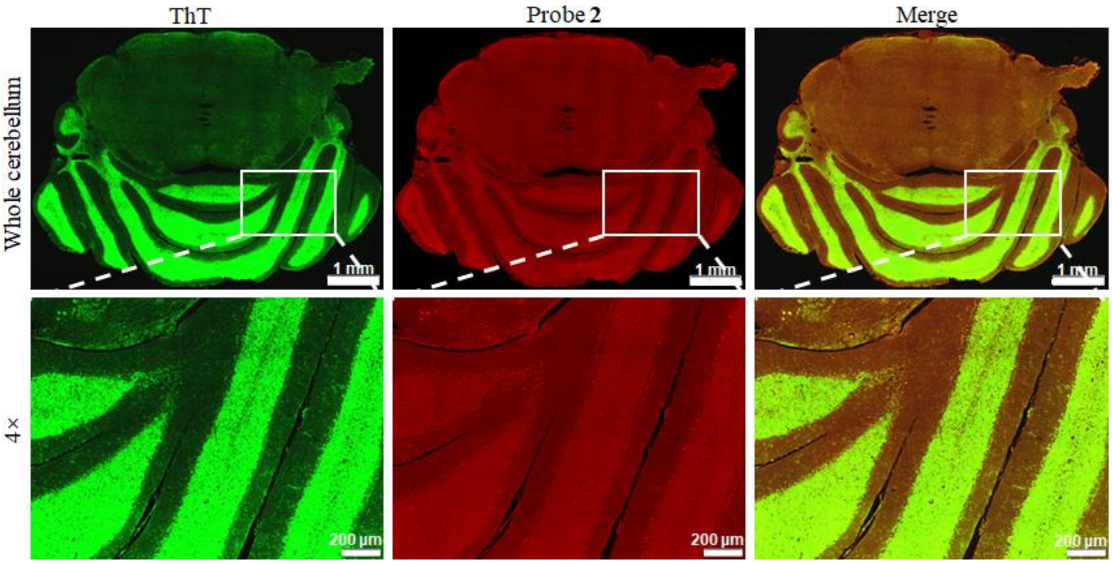

Figure S18. Ex vivo fluorescence imaging of cerebellum tissue slice resected from the same 14-month-old WT mouse in Figure S17 at 30 min after i.v. injection of probe **2** (1mg/kg). The resected cerebellum tissue slice was co-stained with ThT.

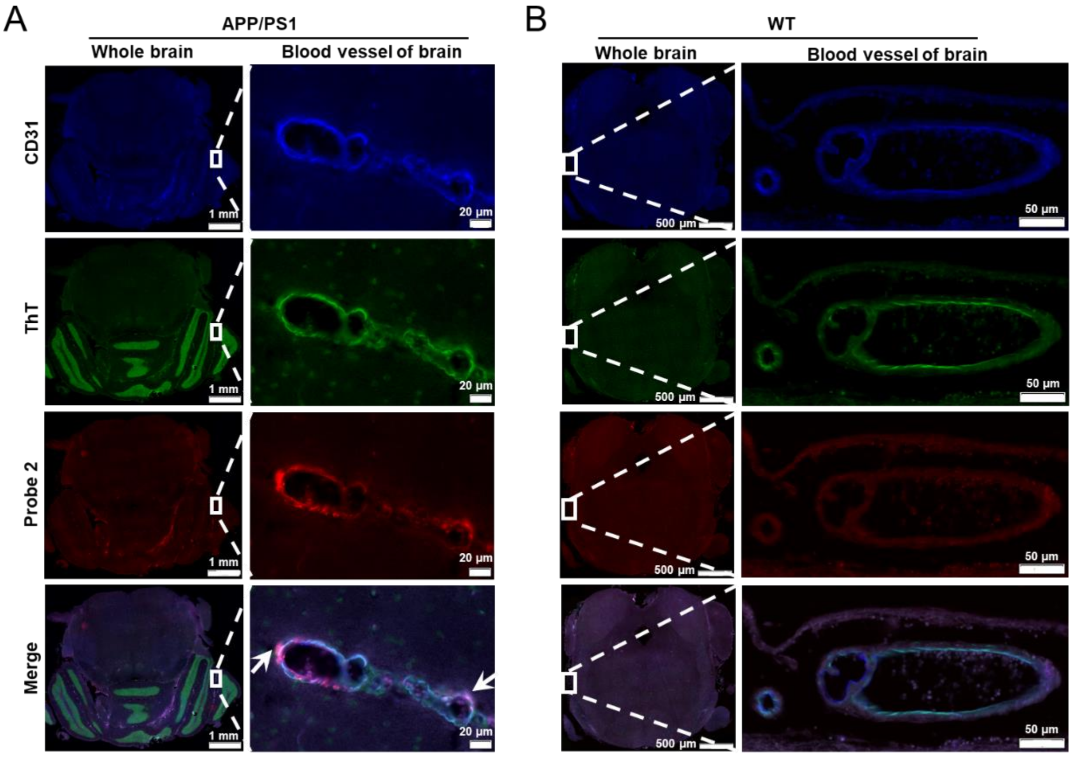

Figure S19. Ex vivo histological staining of CAAs in cerebellum tissue slice from 10-month-old APP/PS1 mouse (A) and WT mouse (B) at 30 min after i.v. injection of probe **2** (1 mg/kg), respectively. The resected cerebrum tissue slice was co-stained with CD31 and ThT respectively. White arrows showed the presence of CAAs in the cerebral vessels.

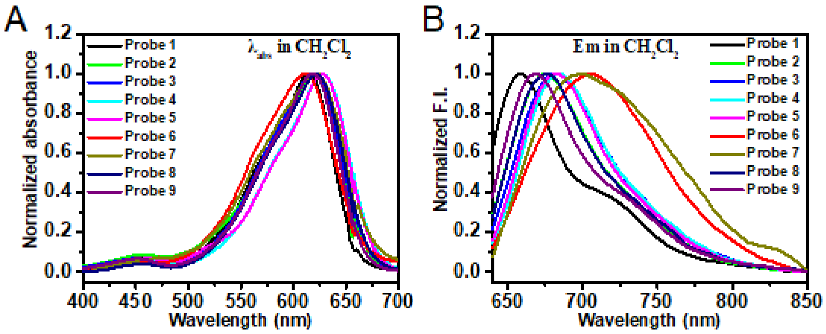

Figure S20. (A) Normalized UV-vis absorption and (B) fluorescence emission spectra of probes **1-9** (2.5 μM) in CH_2Cl_2.

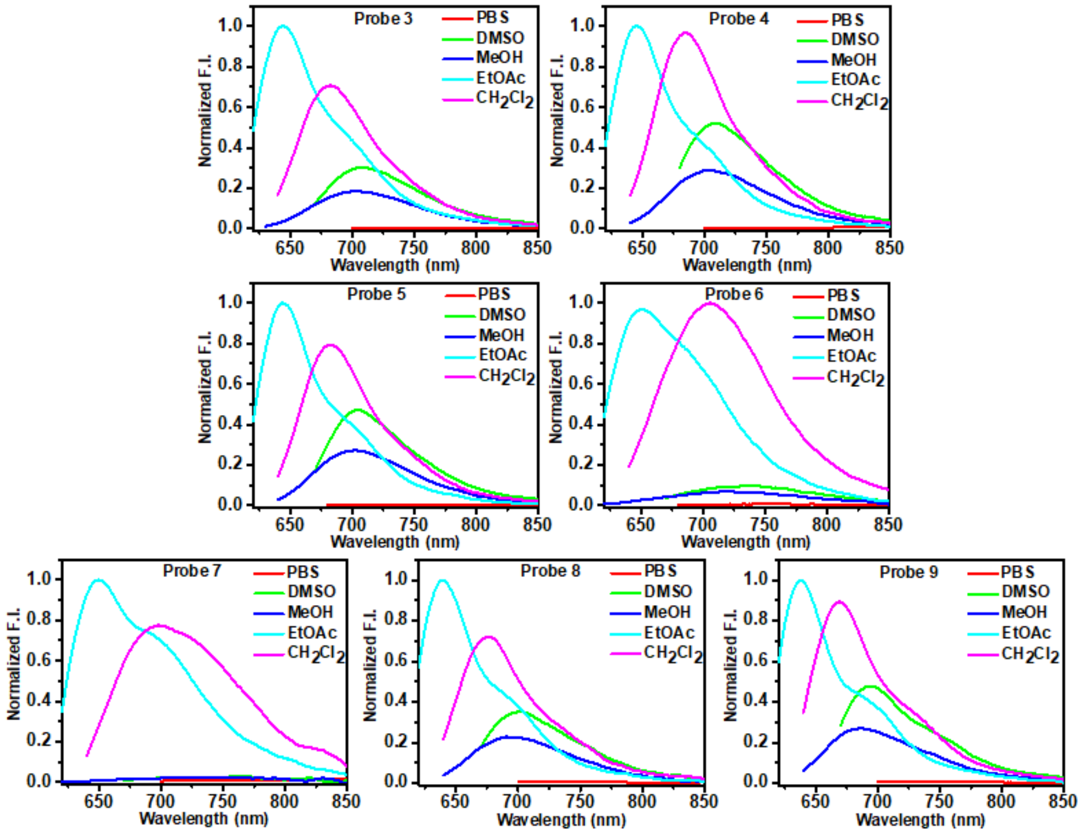

Figure S21. Fluorescence spectra of 2.5 μM probes **3-9** in different solutions. (Note: PBS contained 10% DMSO).

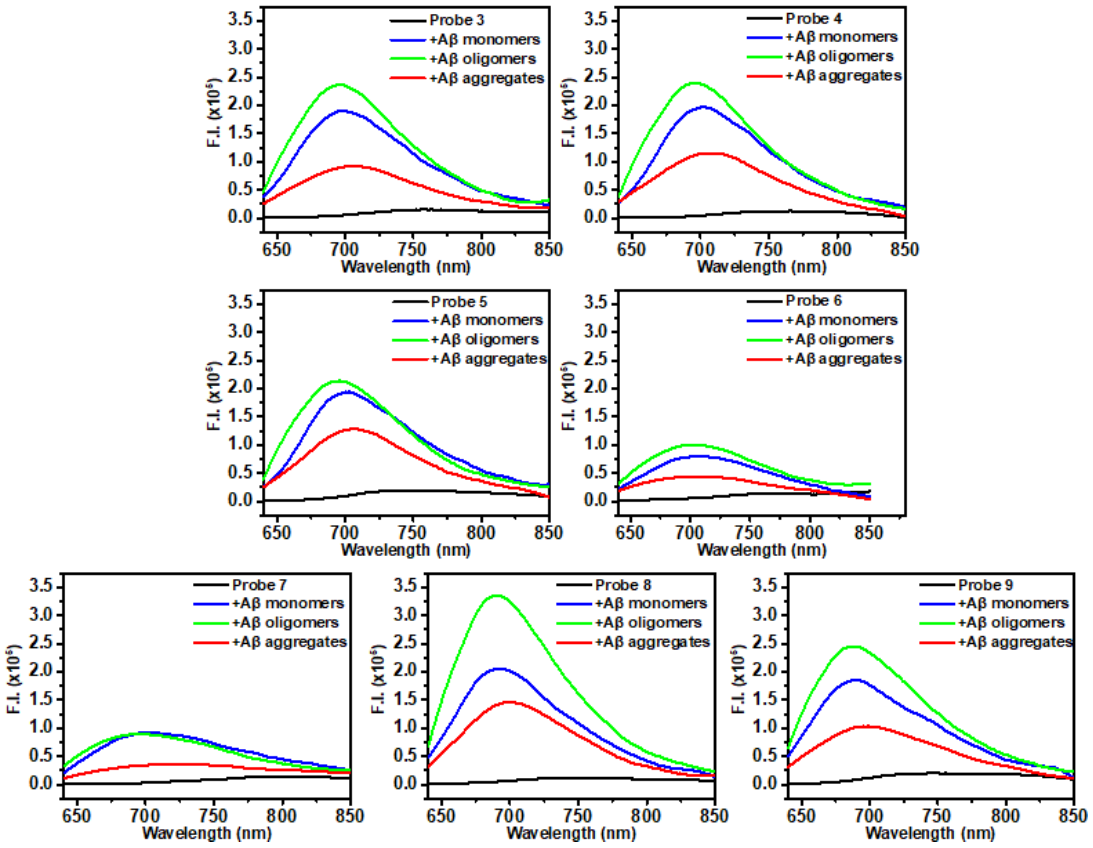

Figure S22. Fluorescence spectra of probes **3**-**9** (250 nM) upon incubation with 250 nM $A\beta_{42}$ monomers, $A\beta_{42}$ oligomers and $A\beta_{42}$ aggregates in PBS buffer (pH = 7.4).

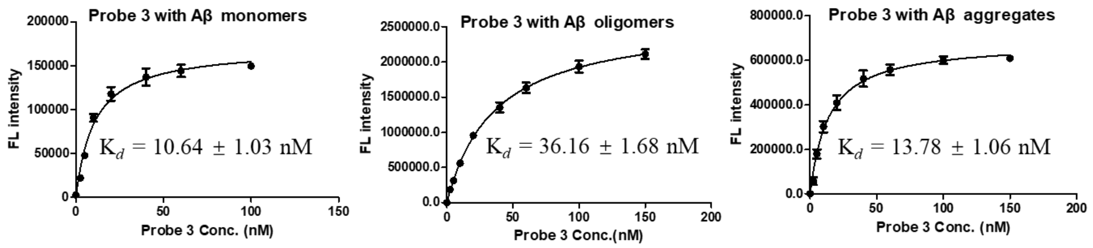

Figure S23. Binding constants (K_d) of probe **3** against 2.5 μM $A\beta_{42}$ monomers, oligomers and aggregates, respectively.

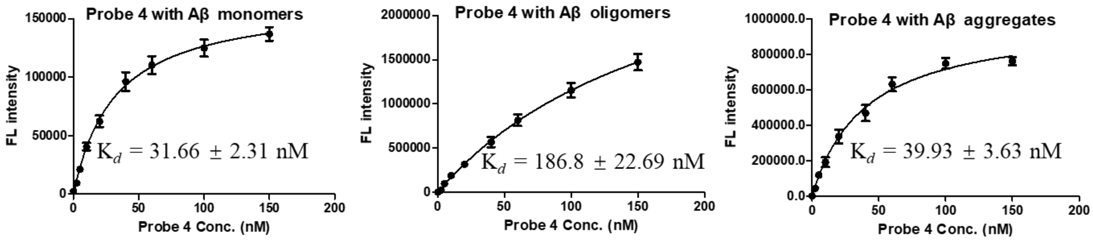

Figure S24. Binding constants (K_d) of probe **4** against 2.5 μM $A\beta_{42}$ monomers, oligomers and aggregates, respectively.

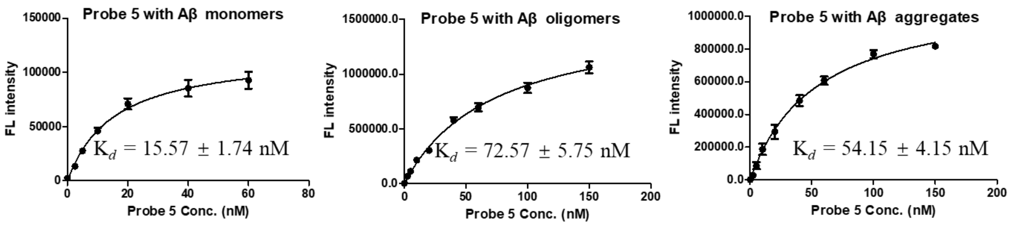

Figure S25. Binding constants (K_d) of probe **5** against 2.5 μM $A\beta_{42}$ monomers, oligomers and aggregates, respectively.

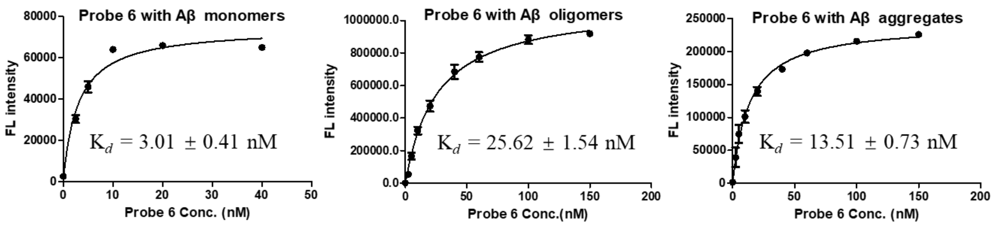

Figure S26. Binding constants (K_d) of probe **6** against 2.5 μM $A\beta_{42}$ monomers, oligomers and aggregates, respectively.

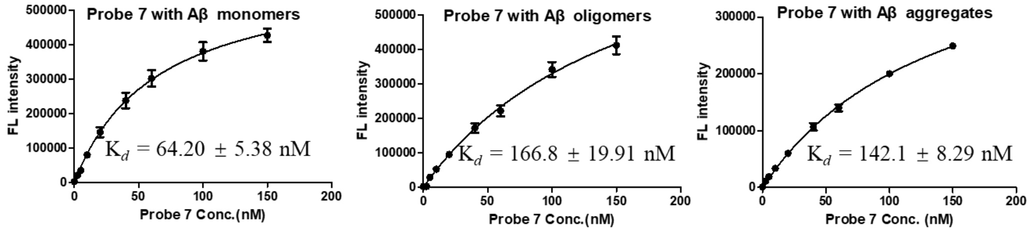

Figure S27. Binding constants (K_d) of probe **7** against 2.5 μM $A\beta_{42}$ monomers, oligomers and aggregates, respectively.

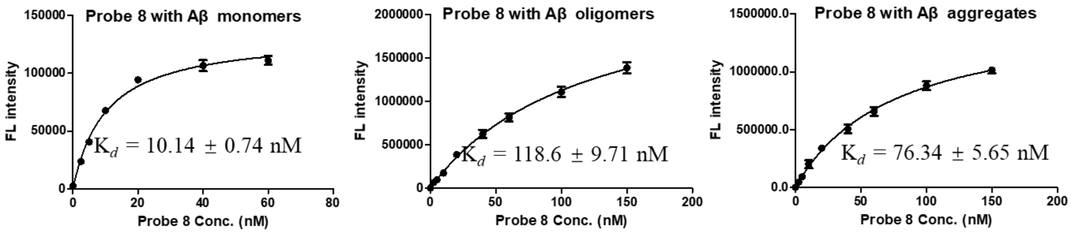

Figure S28. Binding constants (K_d) of probe **8** against 2.5 μM $A\beta_{42}$ monomers, oligomers and aggregates, respectively.

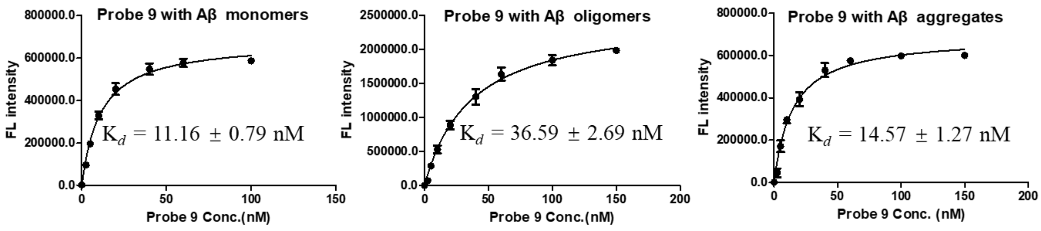

Figure S29. Binding constants (K_d) of probe **9** against 2.5 μM $A\beta_{42}$ monomers, oligomers and aggregates, respectively.

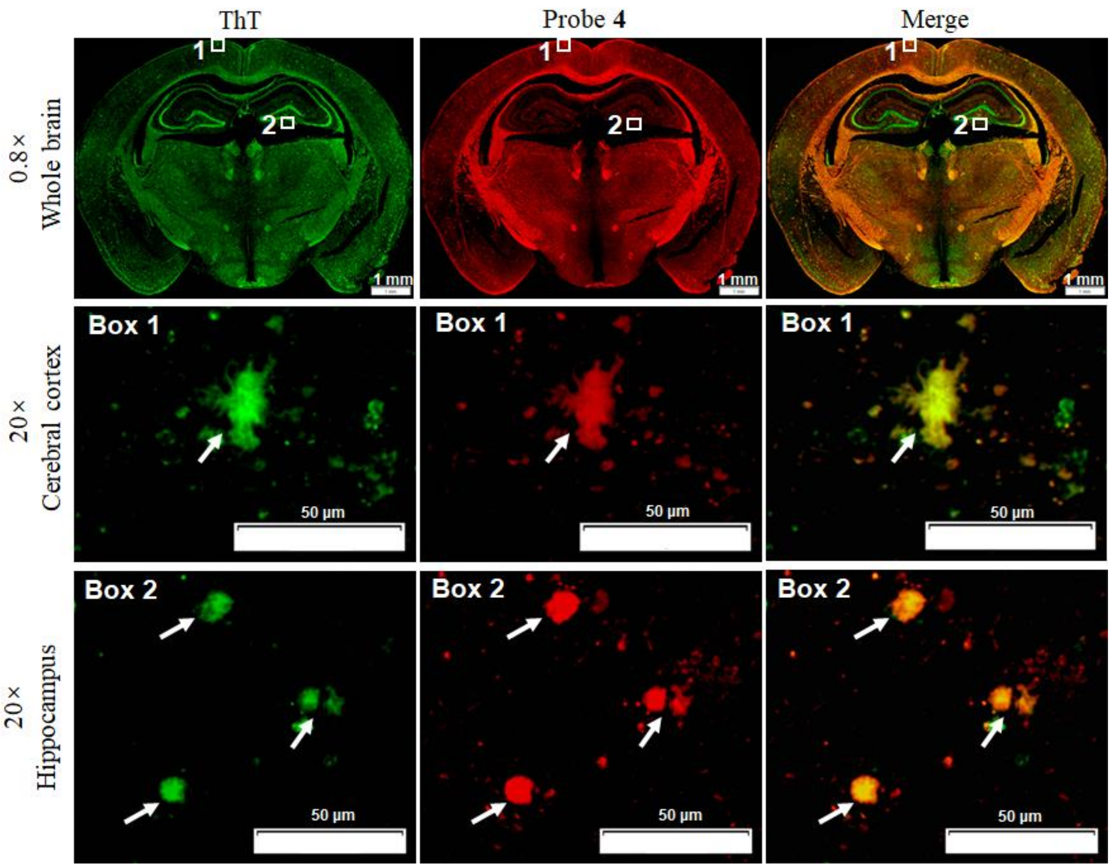

Figure S30. In vitro fluorescence staining of cerebrum tissue slice resected from an APP/PS1 AD mouse with probe **4**. The Aβ plaques in the slice were further confirmed by staining with ThT (green). Boxes 1 and 2 indicated enlarged cerebral cortex and hippocampus, respectively. The white arrows showed that probe **4** could well highlight the Aβ plaques in the cerebral cortex and hippocampus of APP/PS1 mouse brain.

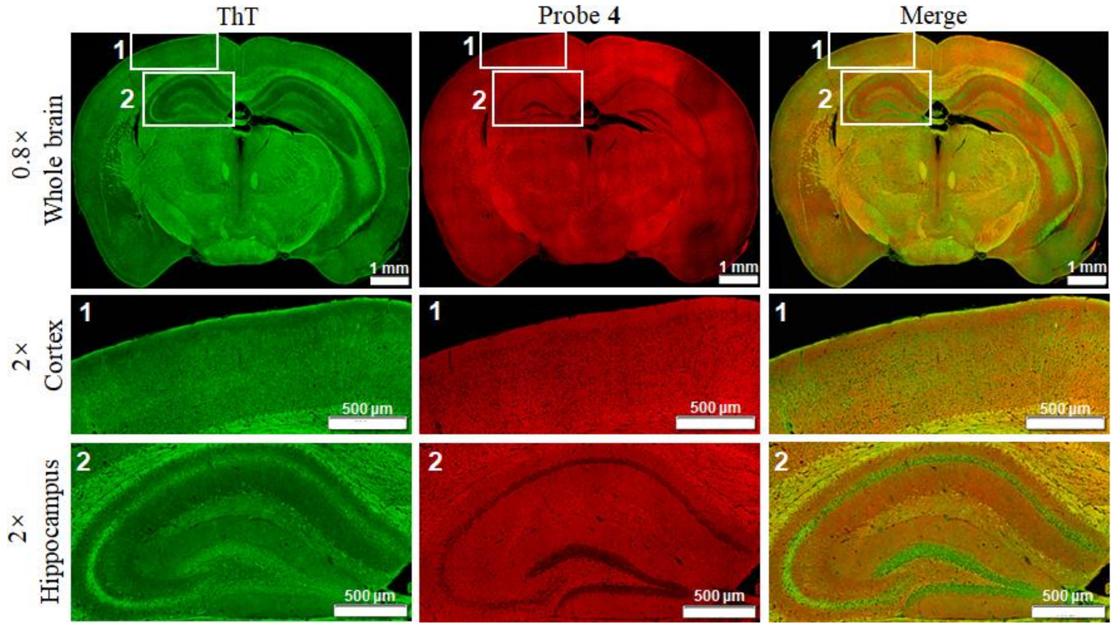

Figure S31. In vitro fluorescence staining of cerebrum tissue slice resected from a WT mouse with probe **4**. Boxes 1 and 2 indicated enlarged cerebral cortex and hippocampus, respectively.

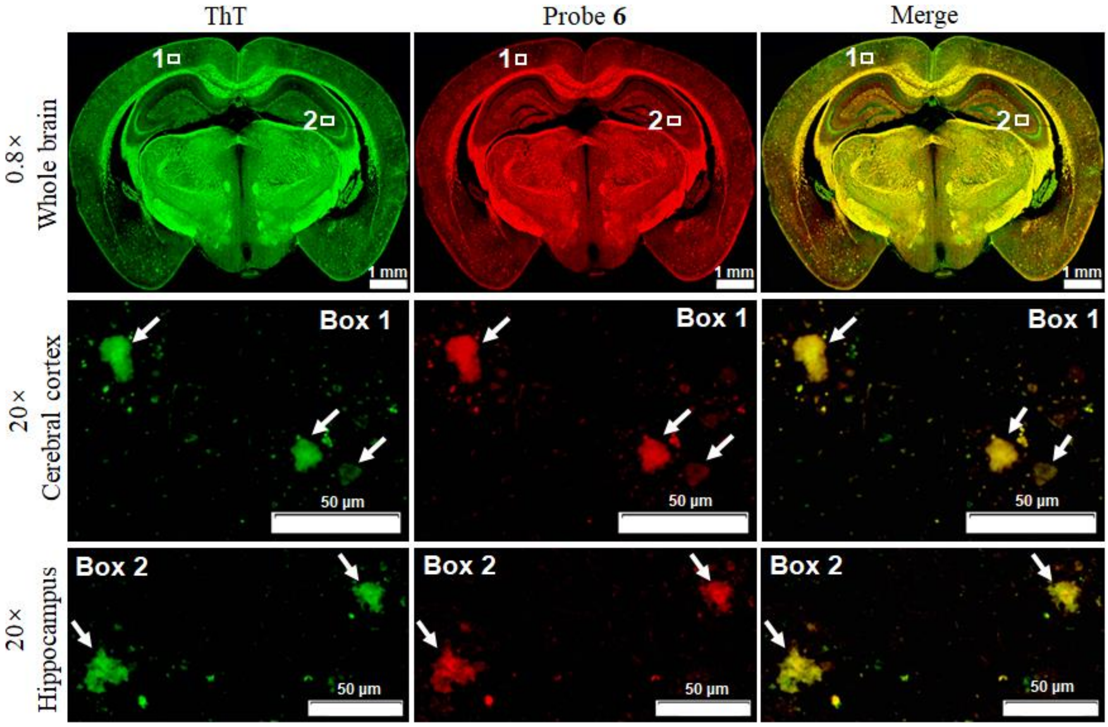

Figure S32. In vitro fluorescence staining of cerebrum tissue slice resected from an APP/PS1 AD mouse with probe **6**. The Aβ plaques in the slice were further confirmed by staining with ThT (green). Boxes 1 and 2 indicated enlarged cerebral cortex and hippocampus, respectively. The white arrows showed that probe **6** could well highlight the Aβ plaques in the cerebral cortex and hippocampus of APP/PS1 mouse brain.

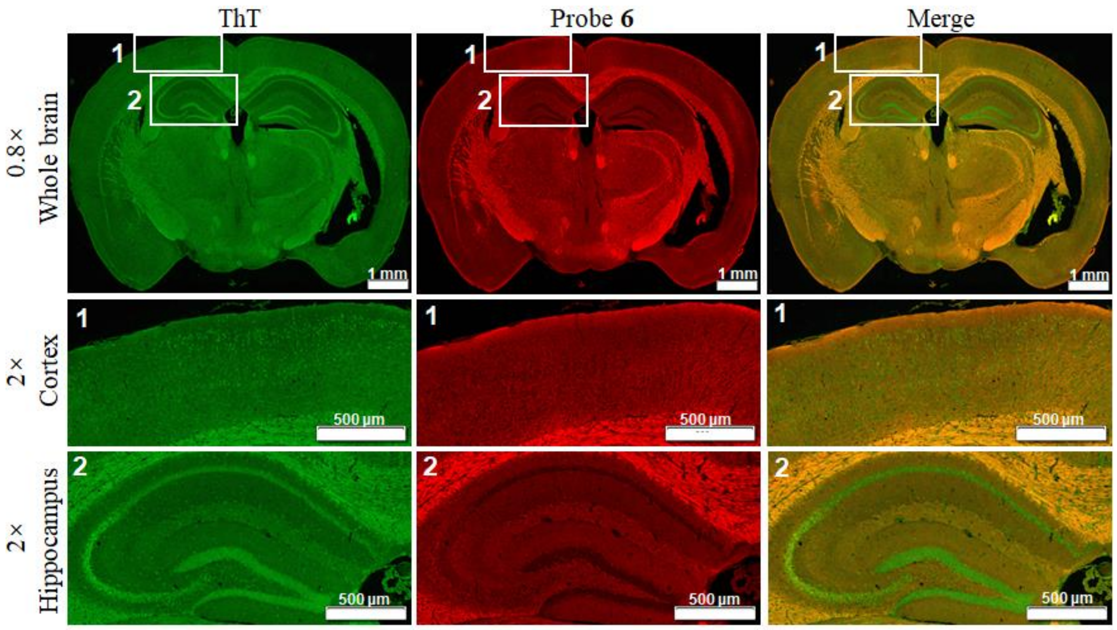

Figure S33. In vitro fluorescence staining of cerebrum tissue slice resected from a WT mouse with probe **6**. Boxes 1 and 2 indicated enlarged cerebral cortex and hippocampus, respectively.

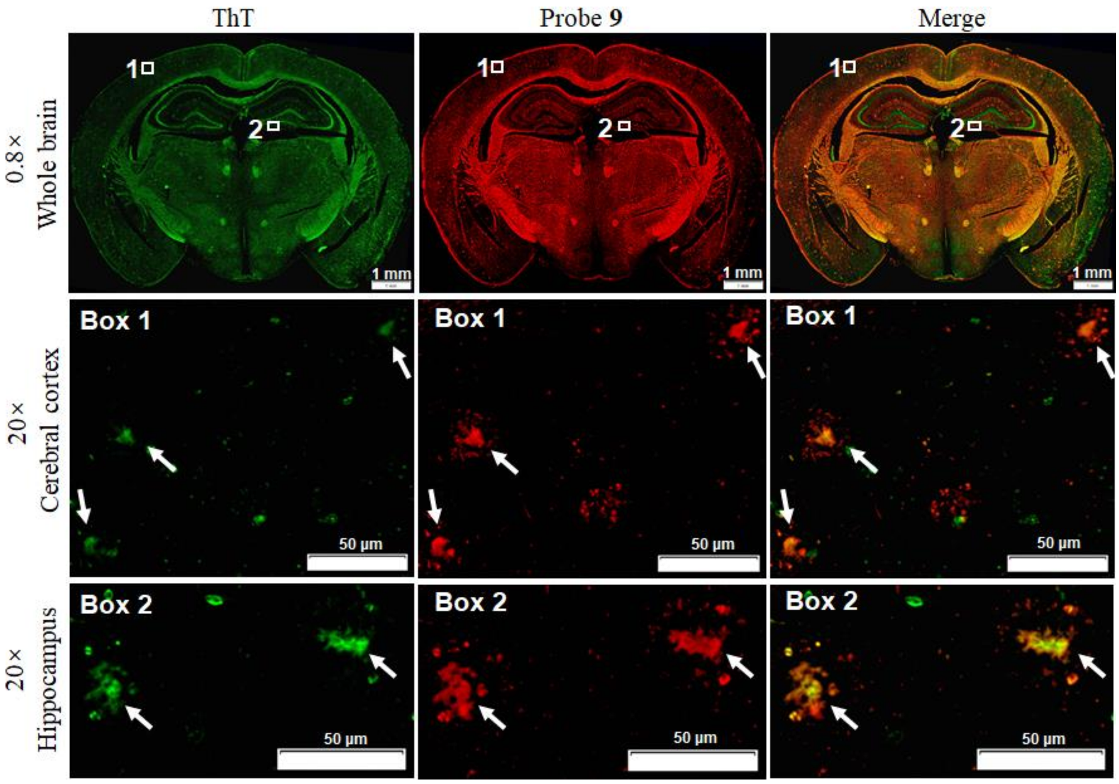

Figure S34. In vitro fluorescence staining of cerebrum tissue slice resected from an APP/PS1 AD mouse with probe **9**. The Aβ plaques in the slice were further confirmed by staining with ThT (green). Boxes 1 and 2 indicated enlarged cerebral cortex and hippocampus, respectively. The white arrows showed that probe **9** could well highlight the Aβ plaques in the cerebral cortex and hippocampus of APP/PS1 mouse brain.

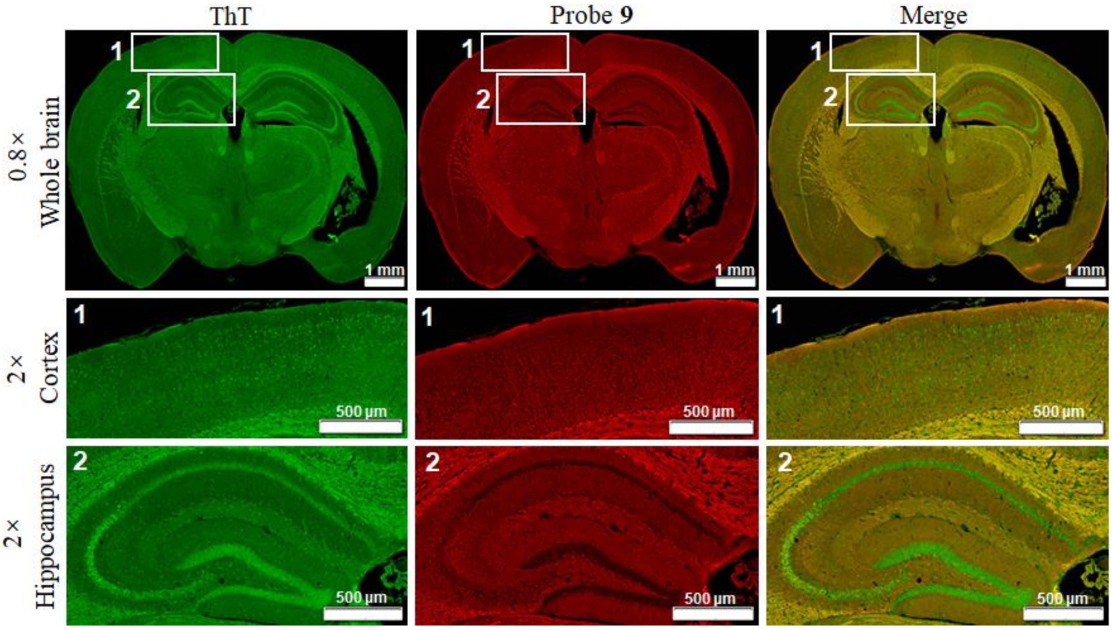

Figure S35. In vitro fluorescence staining of cerebrum tissue slice resected from a WT mouse with probe **9**. Boxes 1 and 2 indicated enlarged cerebral cortex and hippocampus respectively.

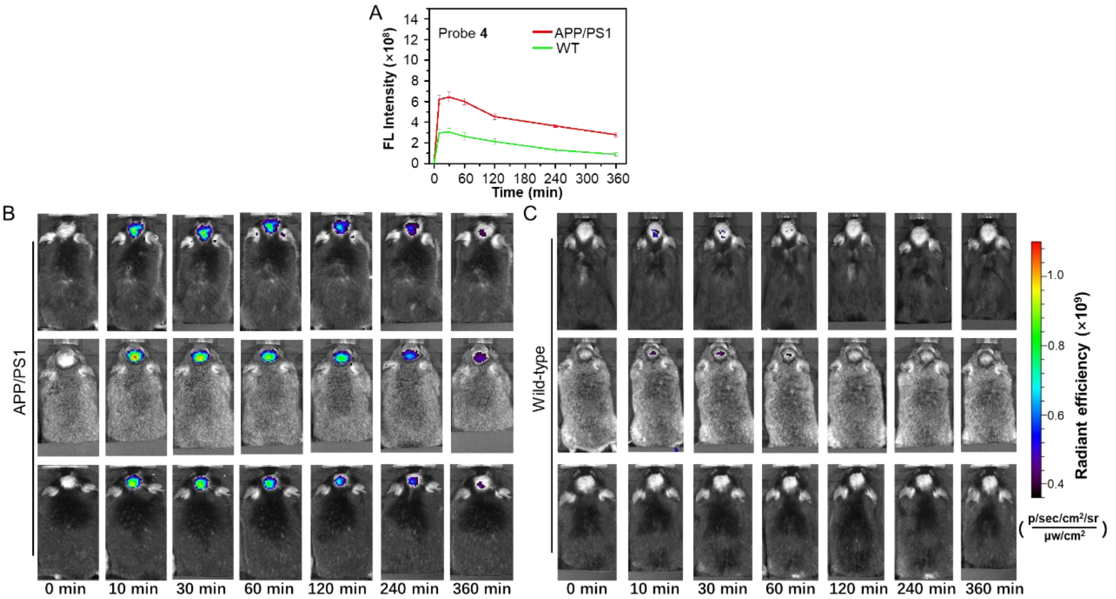

Figure S36. Quantitative analysis of the brain FL intensities of the APP/PS1 and WT mice at indicated time (A). Longitudinal FL images of APP/PS1 transgenic mice (B) and WT mice (C) (10-month old) following i.v. injection of probe **4** (1.0 mg/kg) at 0, 10, 30, 60, 120, 240 and 360 min.

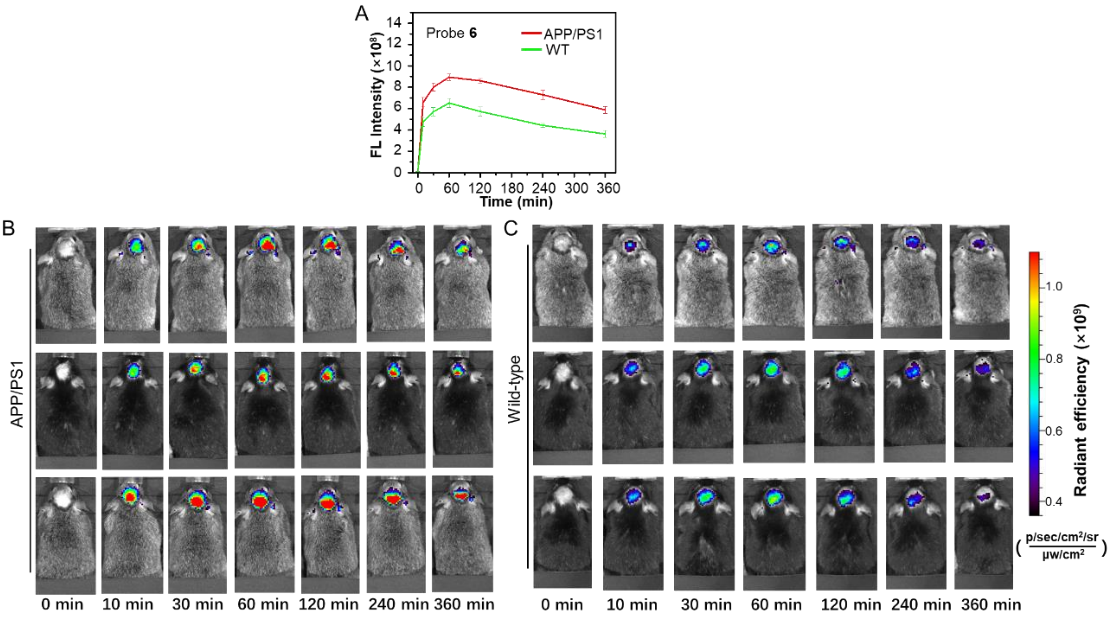

Figure S37. Quantitative analysis of the brain FL intensities of the APP/PS1 and WT mice at indicated time (A). Longitudinal FL images of APP/PS1 transgenic mice (B) and WT mice (C) (10-month old) following i.v. injection of probe **6** (1.0 mg/kg) at 0, 10, 30, 60, 120, 240 and 360 min.

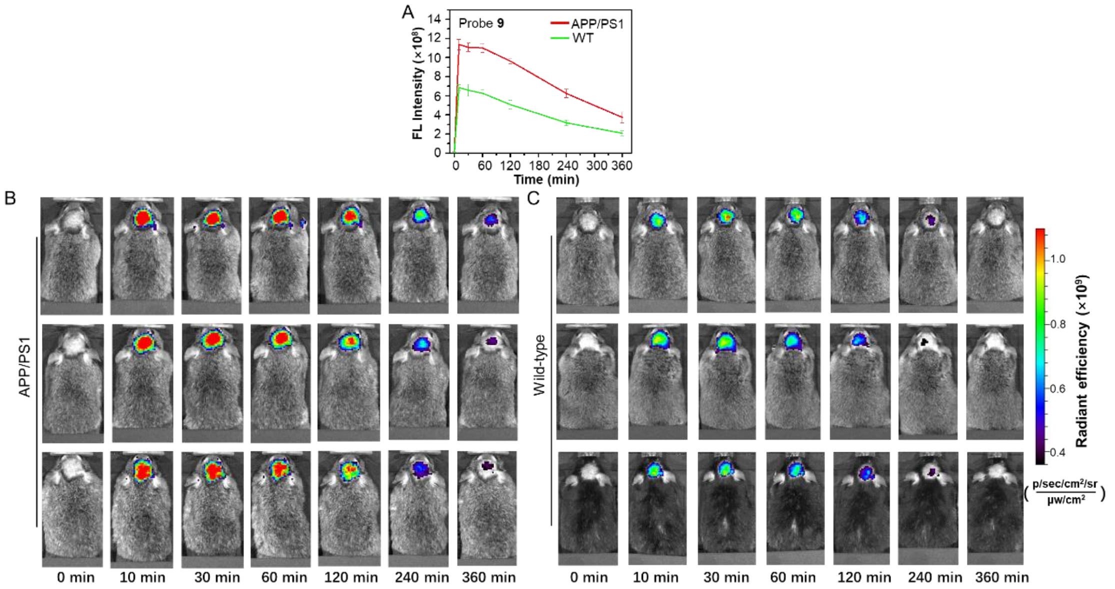

Figure S38. Quantitative analysis of the brain FL intensities of the APP/PS1 and WT mice at indicated time (A). Longitudinal FL images of APP/PS1 transgenic mice (B) and WT mice (C) (10-month old) following i.v. injection of probe **9** (1.0 mg/kg) at 0, 10, 30, 60, 120, 240 and 360 min.

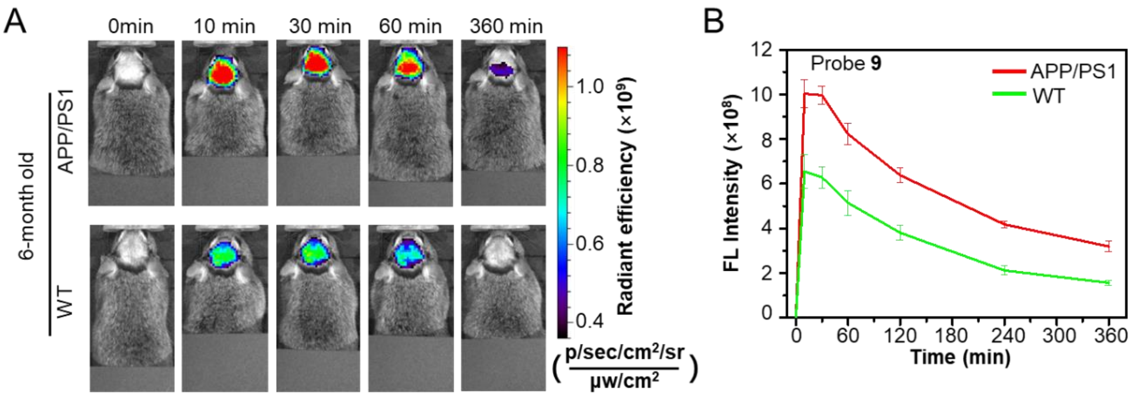

Figure S39. (A) Representative longitudinal FL images of APP/PS1 transgenic and WT mice (6-month old) following i.v. injection of probe **9** (1.0 mg/kg) at 0, 10, 30, 60, and 360 min. (B) Quantitative analysis of the brain FL intensities of the APP/PS1 and WT mice at indicated time. Data are mean ± S.D. (n = 3).

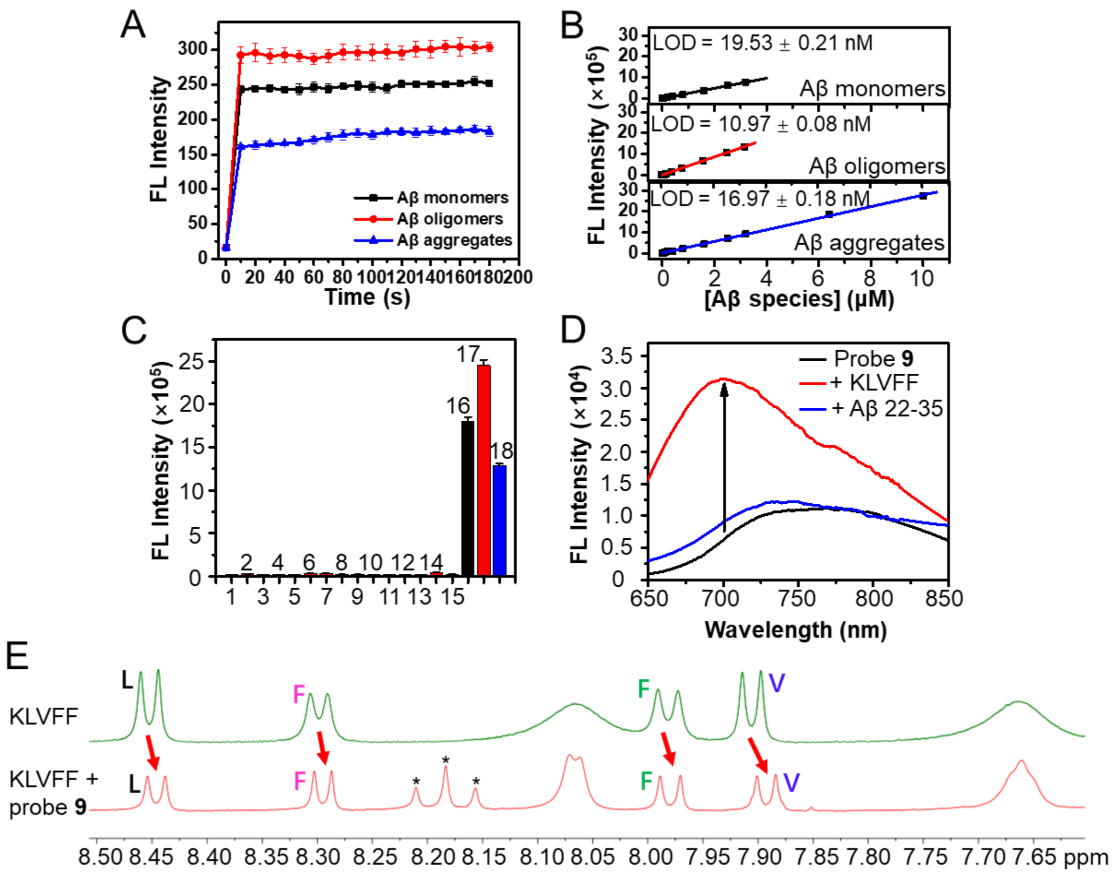

Figure S40. (A) FL intensity ($\lambda_{ex/em}$=620/700 nm) of probe **9** (250 nM) following incubation with 250 nM Aβ_{42} monomers (black), oligomers (red) and aggregates (blue) for 0-180 s. (B) Plots of the mean FL intensity of probe **9** (250 nM) versus varying concentration of Aβ_{42} monomers (black), oligomers (red) and aggregates (blue). (C) Comparison of the FL intensity of 250 nM probe **9** upon incubation with Aβ42 species (10 μg/ml) and other representative endogenous biological species (10 μg/ml) in PBS buffer (1: PBS, 2: OH· (200 μM Fe^{2+} + 1 mM H_2O_2), 3: $^1O_2$ (1 mM H_2O_2 + 1 mM ClO^-), 4: $O_2^{\cdot -}$ (100 μM xanthine + 22 mU xanthine oxidase), 5: H_2O_2 (1 mM H_2O_2), 6: hMAO-A, 7: β-Galactosidase, 8: AChE, 9: BuChE, 10: L-Cysteine, 11: GSH, 12: Cytochrome C, 13: Vitamin C, 14: Amylin, 15: BSA, 16: Aβ_{42} monomers, 17: Aβ_{42} oligomers, 18: Aβ_{42} aggregates). (D) FL spectra of probe **9** (black) and probe **9** (250 nM) incubating with KLVFF (250 nM) or Aβ22-35 (250 nM) peptides. (E) Comparison of the ^{1}H-NMR spectra (DMSO-d_6, 500 MHz) of KLVFF (2.0 mM) in the presence (red) or absence (green) of probe **9** (2.0 mM). Red arrows indicated the change of chemical shifts of the amide protons of L, V and F residues. * indicating the ^{1}H-NMR peaks from probe **9**.

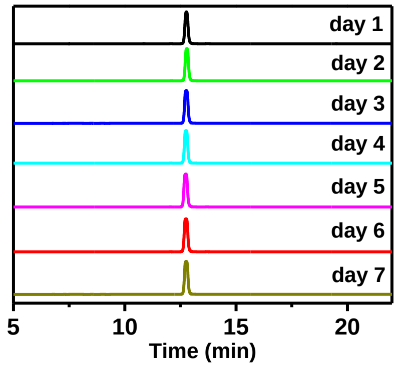

Figure S41. The HPLC traces of probe **9** (250 nM) after incubation in PBS buffer (pH = 7.4) at room temperature for 1-7 days. The HPLC analysis was run using a gradient of 65%-95% acetonitrile-water eluent. Both acetonitrile and water contained 1‰ TFA.

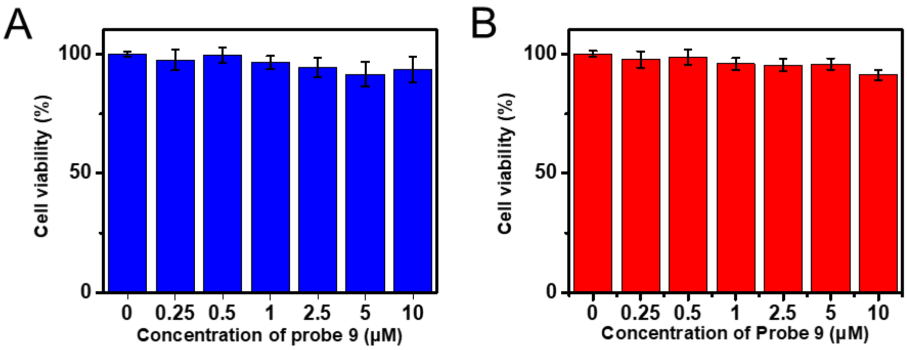

Figure S42. Cell viability of probe **9** towards U87MG cells (A) and PC-12 cells (B) at 0, 0.25, 0.5, 1.0, 2.5, 5.0 and 10.0 μM for 24 h respectively. Data were present as mean ± S.D. (n = 3).

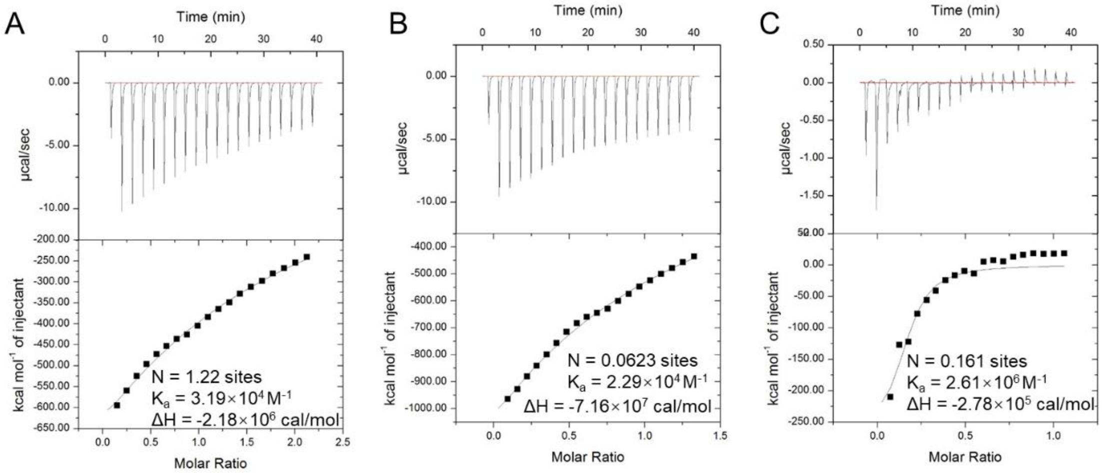

Figure S43. Titration calorimetry curve of probe **9** with Aβ monomers (A), oligomers (B) and aggregates (C) respectively.

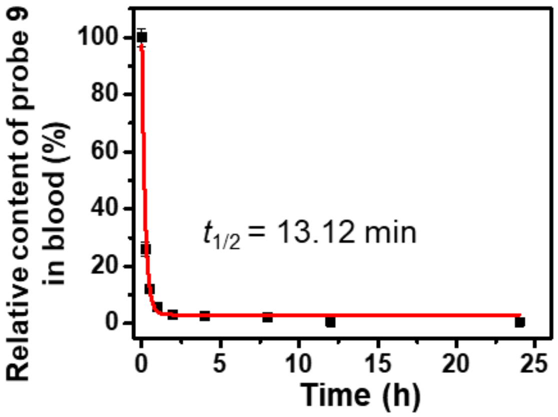

Figure S44. Blood circulation curve of probe **9** (1.0 mg/kg) after i.v. injection into healthy BALB/c mice. Data are mean ± S.D. (n = 3).

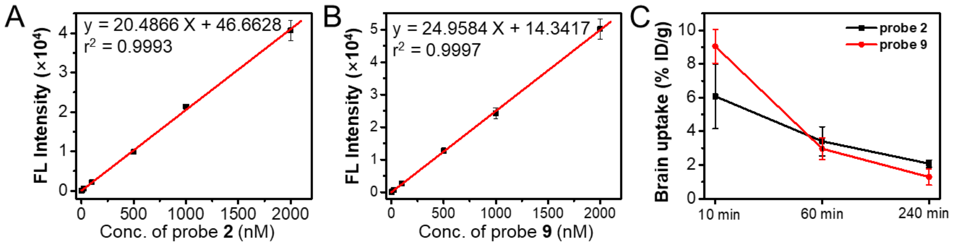

Figure S45. (A) Standard curve of probe **2** in biological simulated solution. (B) Standard curve of probe **9** in biological simulated solution. (C) Comparison of brain uptake of probe **2** (1.0 mg/kg) and probe **9** (1.0 mg/kg) after i.v. injection at 10, 60 and 240 min.

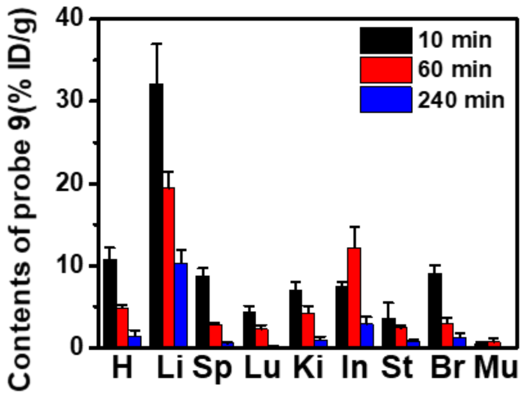

Figure S46. Biodistribution evaluation of probe **9** in BALB/c mice. The mice were i.v. injected with probe **9** (1.0 mg/kg). After 10, 60 and 240 min respectively, the mice were sacrificed, and the main organs (e.g., H: heart, Li: liver including gallbladder, Sp: spleen, Lu: lung, Ki: kidneys, In: intestines, St: stomach, Br: Brain and Mu: muscle) were resected and weighted. The amount of probe **9** in the main organs were determined by standard curve of probe **9** in Figure S45B. Data are mean ± S.D. (n = 3).

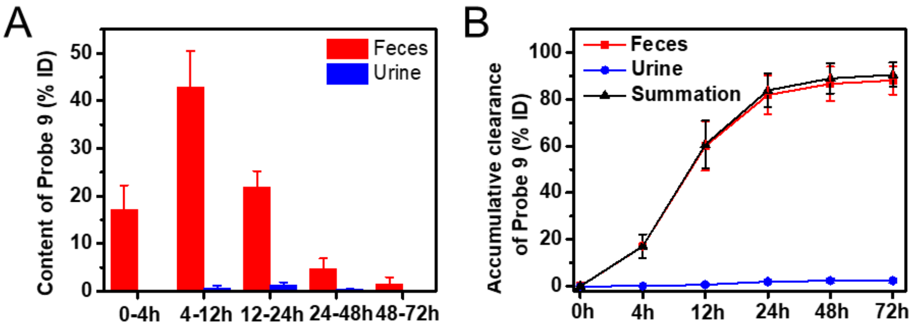

Figure S47. The excretion of probe **9** in healthy BALB/c mice. (A) Contents of probe **9** (% ID) in feces and urine of mice in 0-4, 4-12, 12-24, 24-48 and 48-72 h following i.v. injection of probe **9** (1.0 mg/kg). (B) Plots of the accumulative excretion of probe **9** (% ID) in feces (red), urine (blue), and feces together with urine (summation, black) following i.v. injection of probe **9**. Data are mean ± S.D. (n = 3).

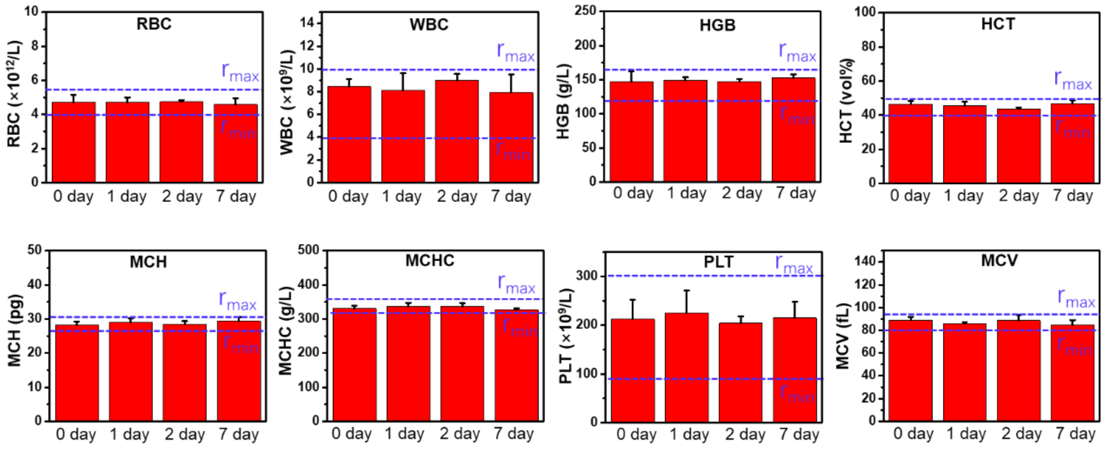

Figure S48. Investigation of the biocompatibility of probe **9** in BALB/c mice. Blood count analysis of BALB/c mice pre (0 d) or after i.v. injection of probe **9** (1.0 mg/kg) at 1, 2 and 7 day. Abbreviations: RBC, red blood cells; WBC, white blood cells; HGB, hemoglobin; HCT, hematocrit; MCH, mean corpuscular hemoglobin; MCHC, mean corpuscular hemoglobin concentration; PLT, platelet count; MCV, mean corpuscular volume. Dotted lines indicate the range of normal reference values (r_{min}→r_{max}) for mice.

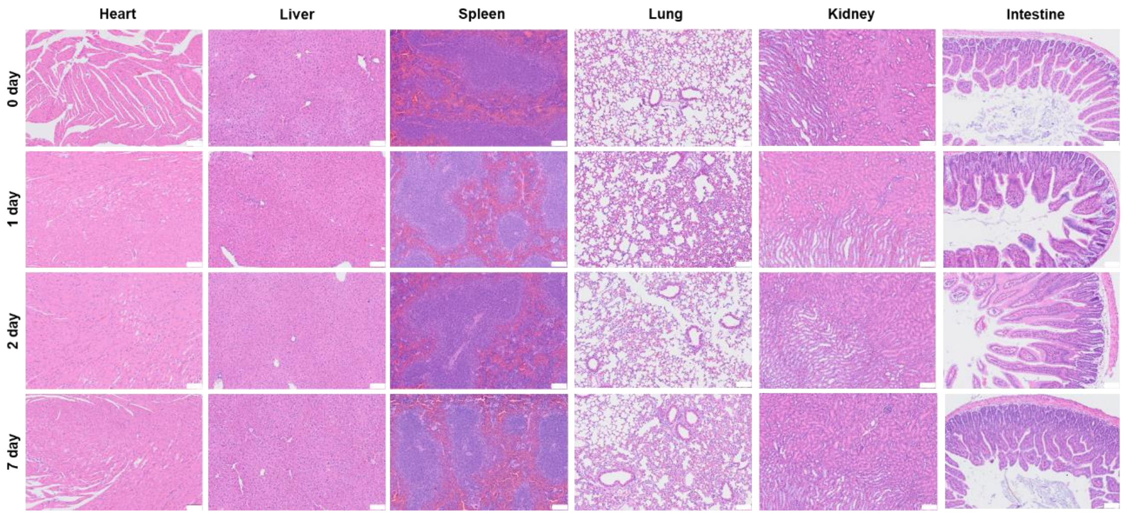

Figure S49. Representative H&E staining of major organs including heart, liver, spleen, lung, kidneys and intestine resected from BALB/c mice pre (0 d) or after i.v. injection of probe **9** (1.0 mg/kg) at 1, 2 and 7 day. Scale bar: 100 μm.

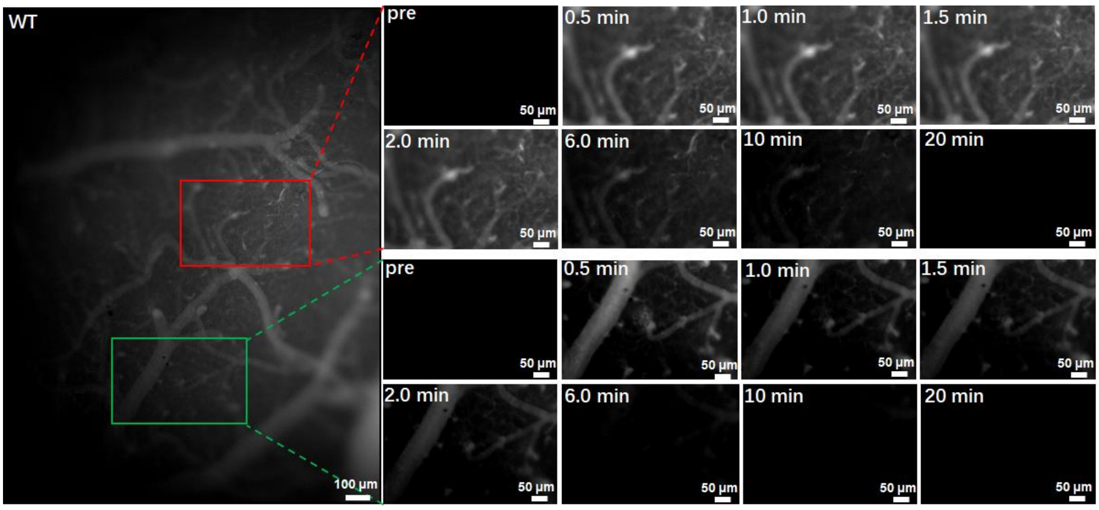

Figure S50. Dynamic fluorescence imaging of the brain of a living skull-thinning 14-month-old WT mouse following i.v. injection of probe **9** (1.0 mg/kg). The fluorescence images were monitored every 5 s on an upright fluorescent microscope, and last for 20 min. Red and green rectangles indicated enlarged brain parenchyma area and cerebral vessel, respectively. The results showed that neither parenchymal Aβ plaques' fluorescence nor CAAs' fluorescence appeared in the age-matched WT mouse during the time course of imaging.

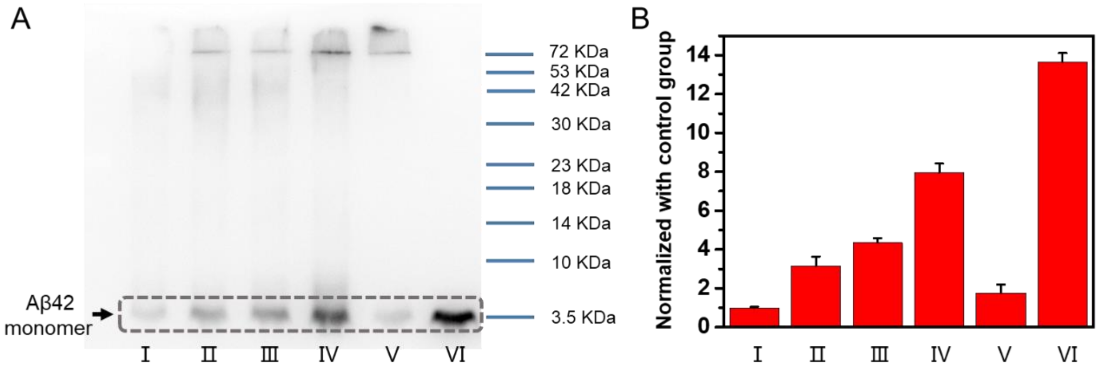

Figure S51. Western blotting of Aβ42 species. (A) Western blotting of $CuSO_4$ + Aβ42 (I) (Control group), $CuSO_4$ + (Aβ42 : probe **9** =1:1) (II), $CuSO_4$ + (Aβ42 : probe **9** =1:5) (III), $CuSO_4$ + (Aβ42 : probe **9** =1:10) (IV), $CuSO_4$ + (Aβ42 : CRANAD-58 =1:10) (V) and native Aβ42 (VI). (B) Quantitative analysis of the monomeric bands in (A). The result indicated that the binding of probe **9** to soluble Aβ monomers could help to slow the process of soluble Aβ species into Aβ aggregates.

2. Supplementary tables

Table S1. Log$P$, HOMO-LUMO energy gap and the spectroscopic properties.

Probes	Log$P$ [a]	HOMO-LUMO gap (eV) [b]	CH_2Cl_2			Φ (%) [c]	
			λ_{abs} (nm)	λ_{em} (nm)	Stokes' shift	CH_2Cl_2	PBS
**1**	2.27	2.79	614	659	45	13.22	0.019
**2**	2.31	2.77	619	675	56	26.34	0.071
**3**	2.39	2.78	618	681	63	18.46	0.014
**4**	2.60	2.76	627	683	58	15.70	0.018
**5**	2.65	2.76	627	682	57	17.28	0.011
**6**	2.42	2.80	611	706	95	8.52	0.006
**7**	2.72	2.75	623	696	73	7.55	0.040
**8**	2.54	2.77	621	675	54	21.02	0.021
**9**	2.14	2.77	618	668	50	20.31	0.020
CRANAD-58	1.86	2.84	581	653	72	9.58	0.016

[a] The Log$P$ values were measured in an octanol/water system.

[b] The HOMO-LUMO gap was estimated based on DFT calculation.

[c] The absolute fluorescence quantum yield was determined using a calibrated integrating sphere.

Table S2. Comparison of the reported NIR fluorescence probes for detection of Aβ species (monomers, oligomers and aggregates)

Probes	QY (Φ)%	$\lambda_{ex}/\lambda_{em}$ (nm)	Aβ monomers[a]		Aβ oligomers[a]		Aβ aggregates[a]		Incubation (*in vitro*)	Fold[b] (*in vivo*)	Tg mouse type	Mouse age (months)	Selectivity	R[c]
			λ_{em} (nm)	K_d (nM)	λ_{em} (nm)	K_d (nM)	λ_{em} (nm)	K_d (nM)						
**AOI-987**	61[d]	644/670[d]	*NR*	*NR*	*NR*	*NR*	705	200	without	*e*	APP23	10	Aggregates	[1]
**NIAD-4**	15[d]	~475/612[d]	*NR*	*NR*	*NR*	*NR*	~600	10	without	*NR*	*NR*	*NR*	Aggregates	[2]
**THK-265**	38.5[d]	627/644[d]	*NR*	*NR*	*NR*	*NR*	650	97 ± 5.0	without	1.7[f]	Tg2576	27	Aggregates	[3]
**PAD-1**	0.5[a]	464/617[a]	*NR*	*NR*	*NR*	*NR*	570	58.9	60 min	*g*	APP/PS1	13	Aggregates	[4]
**QM-FN-SO$_3$**	*NR*	~500/720[h]	*NR*	*NR*	*NR*	*NR*	660	170	40 min	*i*	APP/PS1	22	Aggregates	[5]
**DANIR 2c**	4.09[j]	597/665[a]	*NR*	*NR*	*NR*	*NR*	~620	26.9 ± 3.0	30 min	*k*	APPswe/ PSEN1	22	Aggregates	[6]
**DANIR 3b**	0.3[a]	557/682[a]	*NR*	*NR*	*NR*	*NR*	615	8.8 ± 1.5	1.5 h	1.1	APPswe/ PSEN1	14	Aggregates	[7]
**PHC-4**	0.2[a]	614/798[a]	*NR*	*NR*	*NR*	*NR*	741	14.1 ± 6.4	30 min	*l*	APPswe/ PSEN1	14	Aggregates	[8]
**DANIR-18**	1.9[a]	572/762[a]	*NR*	*NR*	*NR*	*NR*	650	43.1 ± 4.9	60 min	*m*	APPswe/ PSEN1	12	Aggregates	[9]
**MC-1**	0.42[a]	630/695[a]	*NR*	*NR*	*NR*	*NR*	685	59.09 ± 5.6	60 min	*n*	APP/PS1	14	Aggregates	[10]
**CQ**	0.08[a]	516/664[a]	*NR*	*NR*	*NR*	*NR*	654	86 ± 6.3	without	*NR*	*NR*	*NR*	Aggregates	[11]
**TM-1**	*NR*	500/680[a]	*NR*	*NR*	*NR*	*NR*	670	35	without	*NR*	*NR*	*NR*	Aggregates	[12]
**BAP-1**	46.8[o]	604/648[o]	*NR*	*NR*	*NR*	*NR*	~640	44.1	30 min	*p*	Tg2576	25	Aggregates	[13]
**BAP-2**	11.4[o]	650/708[o]	*NR*	*NR*	*NR*	*NR*	708	54.6 ± 7.0	30 min	*q*	Tg2576	25	Aggregates	[14]
**BAP-3**	4.5[o]	663/705[o]	*NR*	*NR*	*NR*	*NR*	705	149 ± 15	30 min	*NR*	*NR*	*NR*	Aggregates	[14]
**BAP-4**	9.3[o]	636/704[o]	*NR*	*NR*	*NR*	*NR*	704	26.8 ± 2.8	30 min	*NR*	*NR*	*NR*	Aggregates	[14]
**BAP-5**	4.3[o]	649/723[o]	*NR*	*NR*	*NR*	*NR*	723	18.1 ± 1.3	30 min	*NR*	*NR*	*NR*	Aggregates	[14]
**F-SLOH**	1.1[r]	483/636[r]	~600	3220	~610	660	~620	1900	3 min	*s*	APP/PS1	7	Oligomers	[15]

**BD-Oligo**	0.087[r]	580/604[r]	~580	*NR*	~580	480	~580	*NR*	*NR*	*NR*	*NR*	*NR*	Oligomers	[16]
**QAD-1**	*NR*	645/755[r]	680	18	700	6	700	27	30 min	~1.5	APPSWE/<br>PSEN<br>1dE9	6	Monomers/<br>Oligomers/<br>Aggregates	[17]
**CRANAD-2**	0.6[a]	640/805[a]	*NR*	*NR*	*NR*	*NR*	715	38.69 ± 2.77	without	0.55[t]	Tg2576	19	Aggregates	[18]
**CRANAD-58**	*NR*	~630/750[a]	672	45.8	~680	*NR*	~700	*NR*	without	1.62	APP/PS1	4	Monomers/<br>Oligomers/<br>Aggregates	[19]
**CRANAD-3**	*NR*	600/730[a]	650	23 ± 1.6	650	27 ± 15.8	~675	21 ± 2.3	without	2.04	APP/PS1	4	Monomers/<br>Oligomers/<br>Aggregates	[20]
**CRANAD-28**	0.32[a]	498/578[a]	~573	159.7 ± 97.3	~575	162.9 ± 34.8	~575	52.4 ± 12.3	without	*NR*	*NR*	*NR*	*u*	[21]
**CRANAD-102**	0.018[a]	560/820[a]	~680	722.8 ± 392.8	700	7.5 ± 10.0	~750	505.9 ± 275.9	15 min	1.22[v]<br>1.89[v]	APP/PS1<br>APP/PS1	4<br>12	Oligomers	[22]
**PTO-29**	0.15[a]	570/680[a]	~660	*NR*	656	248 ± 48	~680	2703 ± 639	15 min	1.34[w]	APP/PS1	4	Oligomers	[23]
**PTO-41**	26[j]	~538/690[a]	~670	*NR*	680	349 ± 41	~670	2651 ± 773	without	1.3[v]	APP/PS1	4	Oligomers	[24]
**CAQ**	1.1[a]	603/726[r]	*NR*	*NR*	*NR*	*NR*	635	78.89	30 min	1.57[v, x]	5× FAD	10	Aggregates	[25]
**Probe 2 (This work)**	26.34[j]	619/675[j]	693	8.64 ± 0.37	692	67.83 ± 4.70	700	28.02 ± 2.00	< 150 s	~1.3	APP/PS1	10	Monomers/<br>Oligomers/<br>Aggregates	
**Probe 9 (This work)**	20.31[j]	618/668[j]	690	11.16 ± 0.79	688	36.59 ±2.69	697	14.57 ±1.27	< 120 s	~1.5<br>~1.7	APP/PS1<br>APP/PS1	6<br>10	Monomers/<br>Oligomers/<br>Aggregates	

NR: Not Reported. [a] determined in PBS buffer. [b] The enhancement ratio of signal from transgenic AD mice and age-matched control mice at 10min. [c] Reference. [d] determined in MeOH. [e] Statistically significant differences between transgenic and wild-type mice were obtained at the 120-min. [f]

at 30 min. [g] The fluorescence signals in the brain regions of the transgenic group were higher than those of the control group at 60 min. [h] determined in 95% EtOH/H_2O. [i] The fluorescence signals in the brain regions of the APP/PS1 mice was much higher than that in the control of wild-type mice at 20 min. [j] determined in CH_2Cl_2. [k] Fluorescence intensities in the brain regions of the Tg mice were higher than in the control mice 30 min. [l] The washout rate in Tg mice was significantly slower than in wild-type (WT) mice. [m] The relative fluorescent signal [F(t)-F(pre)]/F(pre)] of the Tg-Aβ mice was higher than that of the control mice. [n] The fluorescence signals ([F(t)/F(pre)]) displayed an obvious difference between the Tg and WT groups at 30 and 60 min. [o] determined in $CHCl_3$. [p] The fluorescence in whole brains removed at 1 h postinjection of BAP-1 was much higher in the Tg2576 mouse than wild-type mouse. [q] The fluorescence in whole brains removed at 2 hours postinjection of BAP-2 was much higher in the Tg2576 mouse than in the wild-type mouse. [r] determined in DMSO. [s] Higher fluorescence signals were consistently displayed in the Tg mouse compared to that of the WT mouse. [t] The differences of normalized signal between Tg2576 and age-matched control mice. [u] Decreased fluorescence intensity was observed after mixing CRANAD-28 with all Aβ species. [v] The time point was at 30 min. [w] The time point was at 60 min. [x] The was *ex vivo* brain not *in vivo* brain.

Reference:

[1] Hintersteiner M, Enz A, Frey P, Jaton AL, Kinzy W, Kneuer R, *et al*. In vivo detection of amyloid-β deposits by near-infrared imaging using an oxazine-derivative probe. Nat Biotechnol. 2005; 23: S577-83.
[2] Nesterov EE, Skoch J, Hyman BT, Klunk WE, Bacskai BJ, Swager TM. In vivo optical imaging of amyloid aggregates in brain: design of fluorescent markers. Angew Chem Int Ed. 2005; 44: S5452-6.
[3] Okamura N, Mori M, Furumoto S, Yoshikawa T, Harada R, Ito S, *et al*. In vivo detection of amyloid plaques in the mouse brain using the near-infrared fluorescence probe THK-265. J Alzheimer's Dis. 2011; 23: S37-48.
[4] Cheng Y, Zhu B, Deng Y, Zhang Z. In vivo detection of cerebral amyloid fibrils with smart dicynomethylene-4h-pyran-based fluorescence probe. Anal Chem. 2015; 87: S4781-7.

[5] Fu W, Yan CX, Guo ZQ, Zhang JJ, Zhang HY, Tian H, *et al*. Rational design of near-infrared aggregation-induced-emission active probes: in situ mapping of amyloid-β plaques with ultrasensitivity and high-fidelity. J Am Chem Soc. 2019; 141: S3171−7.
[6] Cui MC, Ono M, Watanabe H, Kimura H, Liu BL, Saji H. Smart near-infrared fluorescence probes with donor−acceptor structure for in vivo detection of β-amyloid deposits. J Am Chem Soc. 2014; 136: S3388−94.
[7] Fu HL, Cui MC, Zhao L, Tu PY, Zhou KX, Dai JP, *et al*. Highly sensitive near-infrared fluorophores for in vivo detection of amyloid-β plaques in Alzheimer's disease. J Med Chem. 2015; 58: S6972−83.
[8] Zhou KX, Bai HC, Feng L, Dai JP, Cui MC. Smart D-π-A type near-infrared Aβ probes: effects of a marked π bridge on optical and biological properties. Anal Chem. 2017; 89: S9432−7.
[9] Zhou KX, Yuan C, Dai B, Wang K, Chen YM, Ma DL, *et al*. Environment-sensitive near-infrared probe for fluorescent discrimination of Aβ and Tau fibrils in AD brain. J Med Chem. 2019; 62: S6694−704.
[10] Yan JW, Zhu JY, Zhou KX, Wang JS, Tan HY, Xu ZY. *et al*. Neutral merocyanine dyes: for in vivo NIR fluorescence imaging of amyloid-β plaques. Chem Commun. 2017; 53: S9910−3.
[11] Rajasekhar K, Narayanaswamy N, Murugan NA, Viccaro K, Lee HG, Shah K, *et al*. Aβ plaque-selective NIR fluorescence probe to differentiate Alzheimer's disease from tauopathies. Biosens Bioelectron. 2017; 98: S54−61.
[12] Xu MY, Li RH, Li X, Lv GL, Li SP, Sun A, *et al*. NIR fluorescent probes with good water-solubility for detection of amyloid beta aggregates in Alzheimer's disease. J Mater Chem. B, 2019; 7: S5535−40.
[13] Ono M, Watanabe H, Kimura H, Saji H. BODIPY-based molecular probe for imaging of cerebral β-amyloid plaques. ACS Chem Neurosci. 2012; 3: S319-24.
[14] Watanabe H, Ono M, Matsumura K, Yoshimura M, Kimura H, Saji H. Molecular imaging of β-amyloid plaques with near-infrared boron Dipyrromethane (BODIPY)-based fluorescent probes. Mol Imaging, 2013; 12: S338−47.
[15] Li YH, Xu D, Sun AY, Ho SL, Poon CY, Chan HN, *et al*. Fluoro-substituted cyanine for reliable in vivo labelling of amyloid-β oligomers and neuroprotection against amyloid-β induced toxicity. Chem Sci. 2017; 8: S8279–84.
[16] Teoh CL, Su D, Sahu S, Yun SW, Drummond E, Prelli F, *et al*. Chemical fluorescent probe for detection of Aβ oligomers. J Am Chem Soc. 2015; 137: S13503−9.
[17] Ren WM, Zhang JJ, Peng C, Xiang HJ, Chen JJ, Peng CY, *et al*. Fluorescent imaging of β-amyloid using BODIPY based near-infrared off–on fluorescent probe. Bioconjugate Chem. 2018; 29: S3459−66.

[18]Ran C, Xu X, Raymond SB, Ferrara BJ, Neal K, Bacskai BJ, *et al*. Design, synthesis, and testing of difluoroboron-derivatized curcumins as near-infrared probes for in vivo detection of amyloid-β deposits. J Am Chem Soc. 2009; 131: S15257-61.
[19] Zhang X, Tian Y, Li Z, Tian X, Sun H, Liu H, *et al*. Design and synthesis of curcumin analogues for in vivo fluorescence imaging and inhibiting copper-induced cross-linking of amyloid beta species in Alzheimer's disease. J Am Chem Soc. 2013; 135: S16397-409.
[20] Zhang X, Tian Y, Zhang C, Tian X, Ross AW, Moir RD, *et al*. Near-infrared fluorescence molecular imaging of amyloid beta species and monitoring therapy in animal models of Alzheimer's disease. Proc Natl Acad Sci U. S. A. 2015; 112: S9734-9.
[21] Zhang X, Tian Y, Yuan P, Li Y, Yaseen MA, Grutzendler J, *et al*. A bifunctional curcumin analogue for two-photon imaging and inhibiting crosslinking of amyloid beta in Alzheimer's disease. Chem Commun. 2014; 50: S11550-3.
[22]Li Y, Yang J, Liu H, Yang J, Du L, Feng H, et al. Tuning the stereo-hindrance of a curcumin scaffold for the selective imaging of the soluble forms of amyloid beta species. Chem Sci. 2017; 8: S7710-7.
[23]Yang J, Zeng F, Li X, Ran C, Xu Y, Li Y. Highly specific detection of Aβ oligomers in early Alzheimer's disease by a near-infrared fluorescent probe with a "V-shaped" spatial conformation. Chem Commun. 2020; 56: S583-6.
[24] Zeng F, Yang J, Li X, Peng K, Ran C, Xu Y, *et al*. Versatile near-infrared fluorescent probe for in vivo detection of Aβ oligomers. Bioorg Med Chem. 2020; 28: S115559.
[25] Wu J, Shao CW, Ye XL, Di XJ, Li DD, Zhao H, *et al*. In vivo brain imaging of amyloid-β aggregates in Alzheimer's disease with a near-infrared fluorescent probe. ACS Sens. 2021; 6: S863−70.

4. NMR and MS spectra

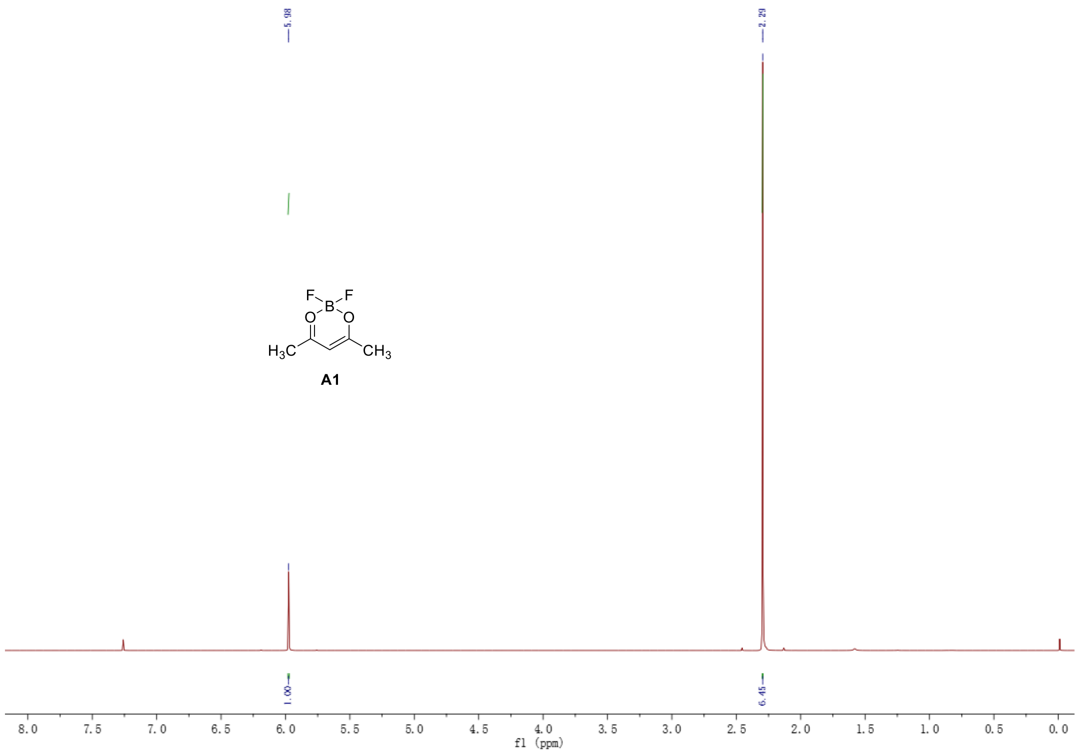

^{1}H-NMR spectra of **A1** (400 MHz, $CDCl_3$)

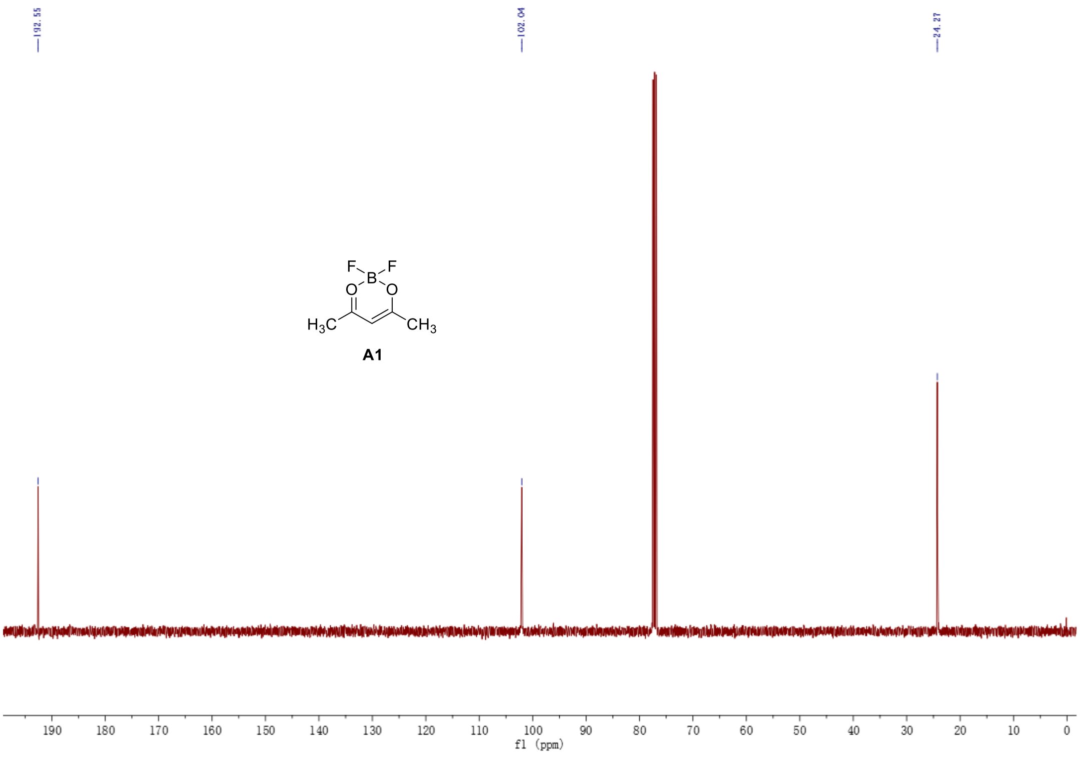

^{13}C-NMR spectra of **A1** (101 MHz, $CDCl_3$)

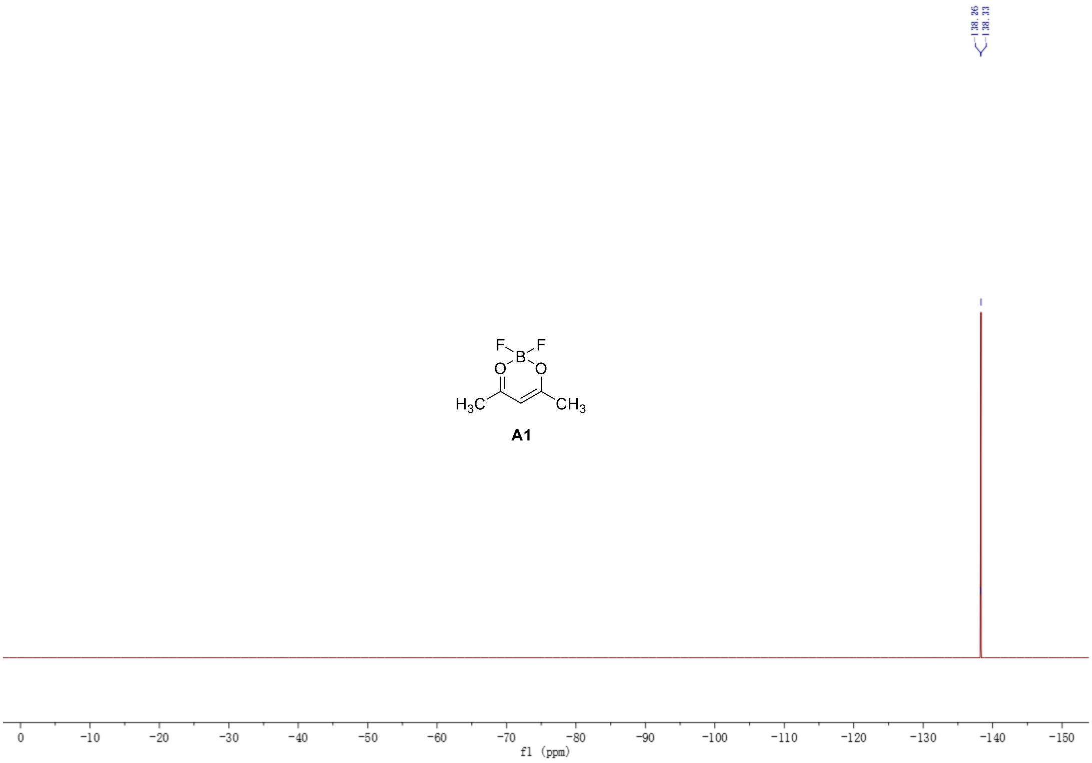

^{19}F-NMR spectrum of **A1** ($CDCl_3$)

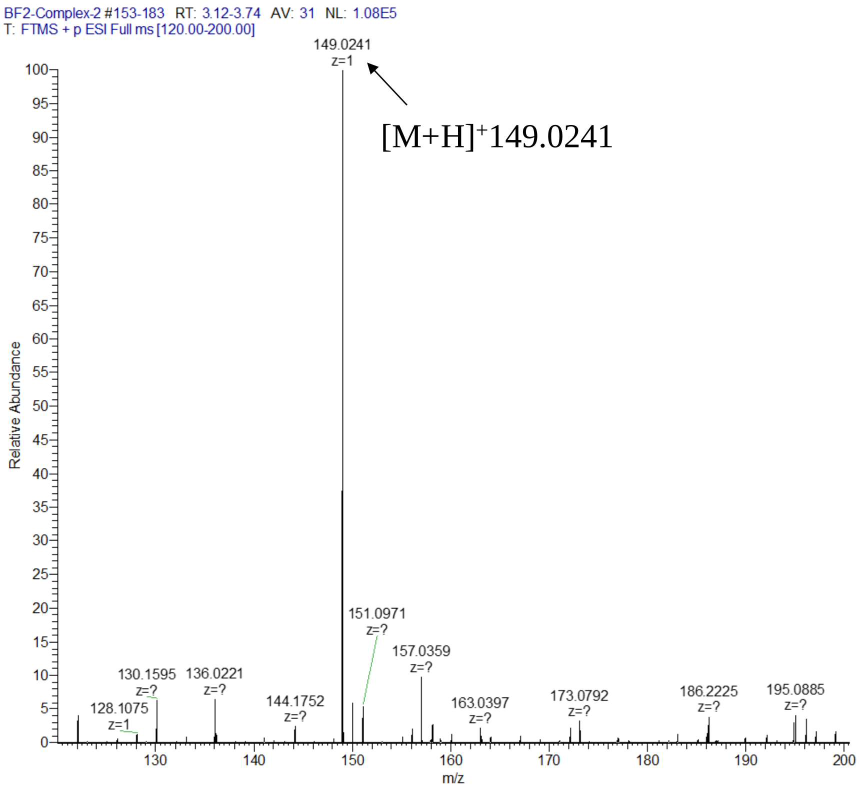

HRMS spectrum of **A1**

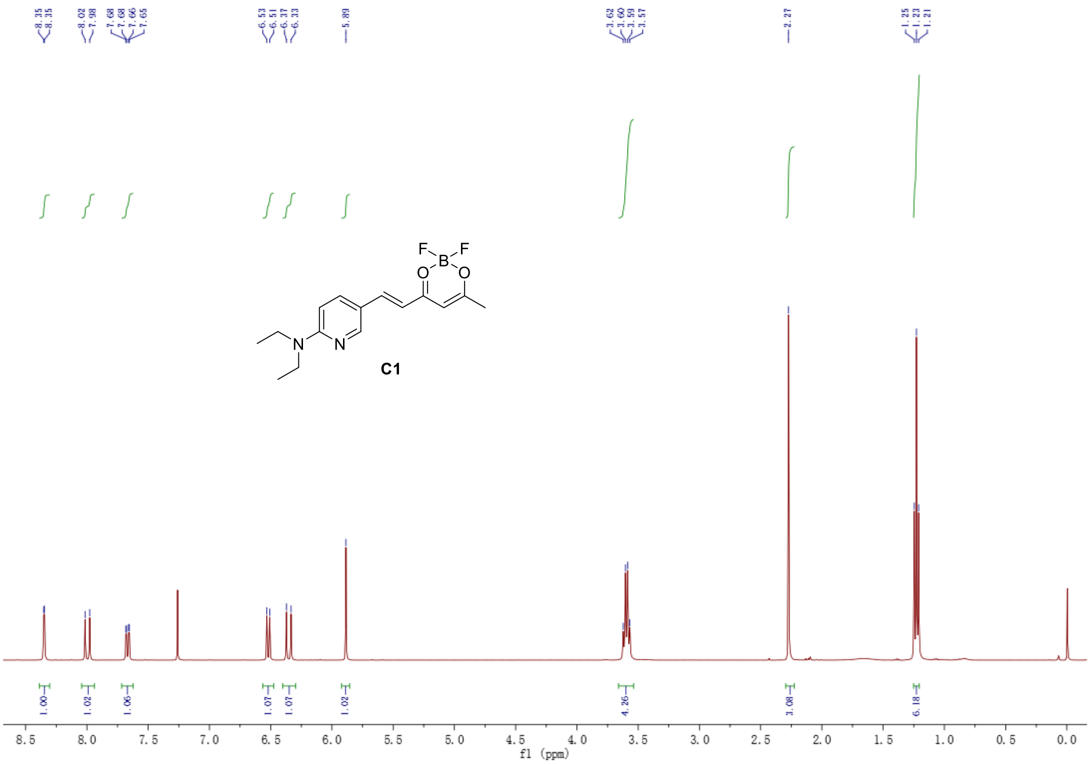

^{1}H-NMR spectra of **C1** (400 MHz, $CDCl_3$)

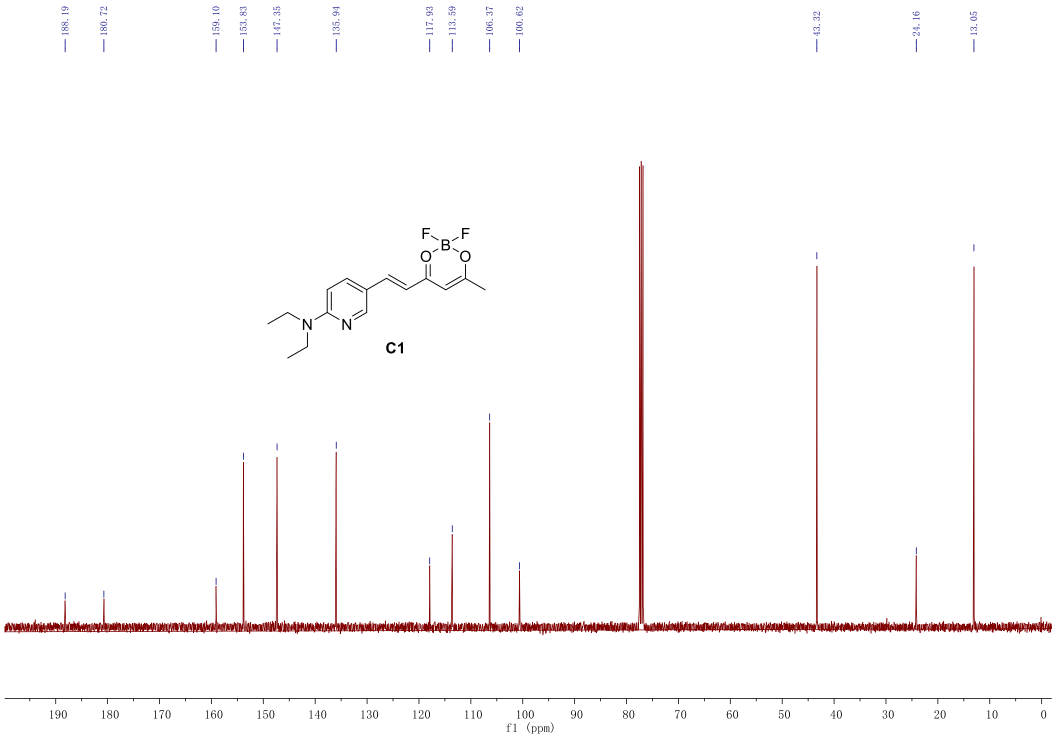

^{13}C-NMR spectra of **C1** (101 MHz, $CDCl_3$)

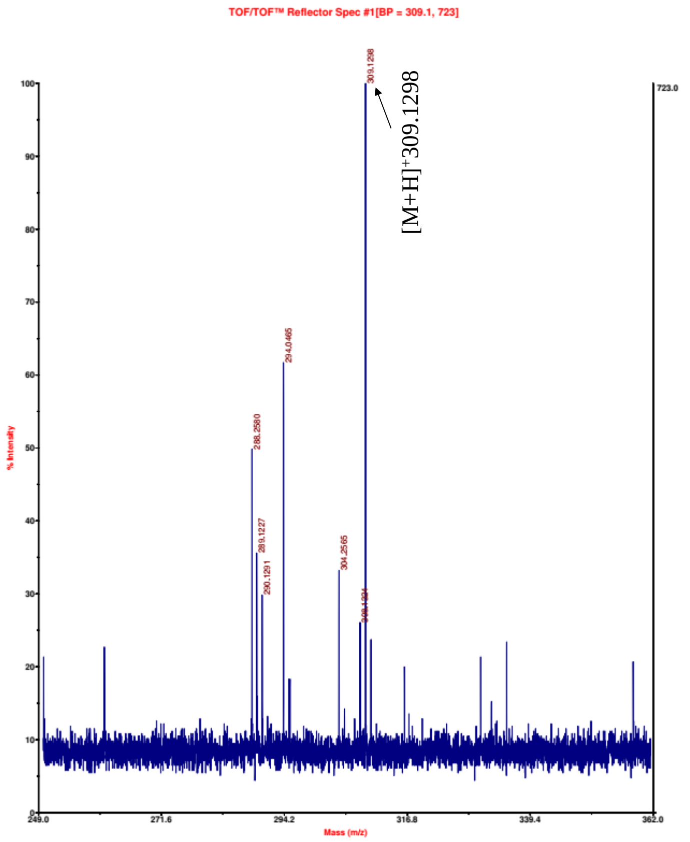

MALDI-TOF-MS spectrum of **C1**

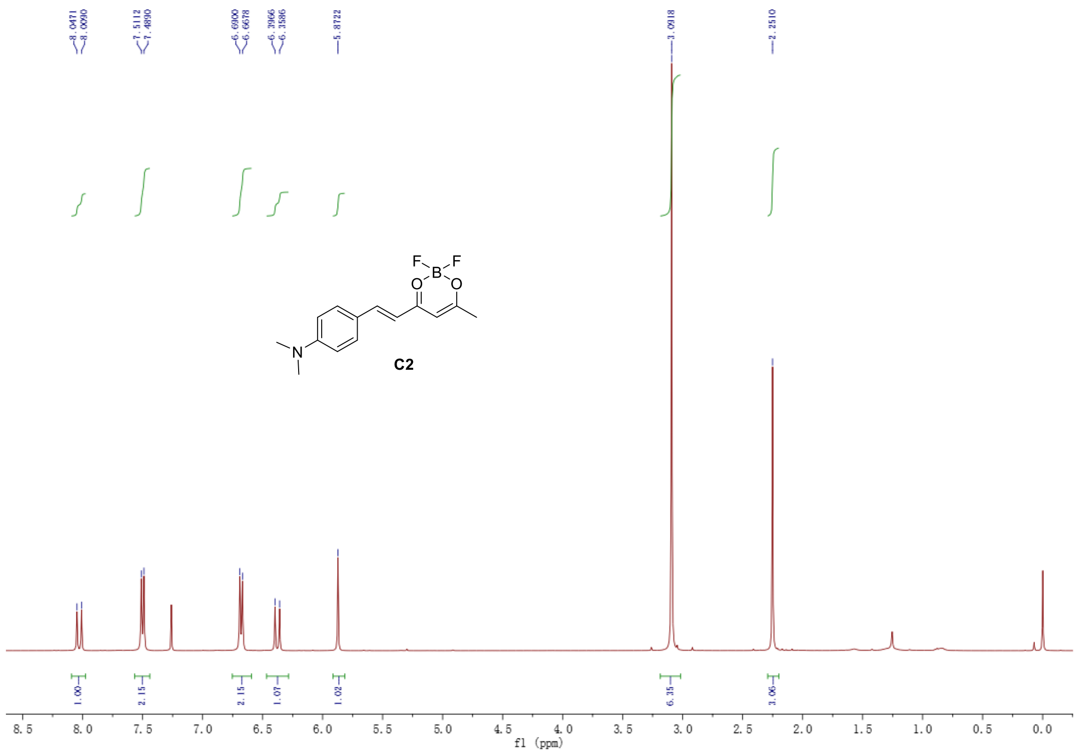

^{1}H-NMR spectra of **C2** (400 MHz, $CDCl_3$)

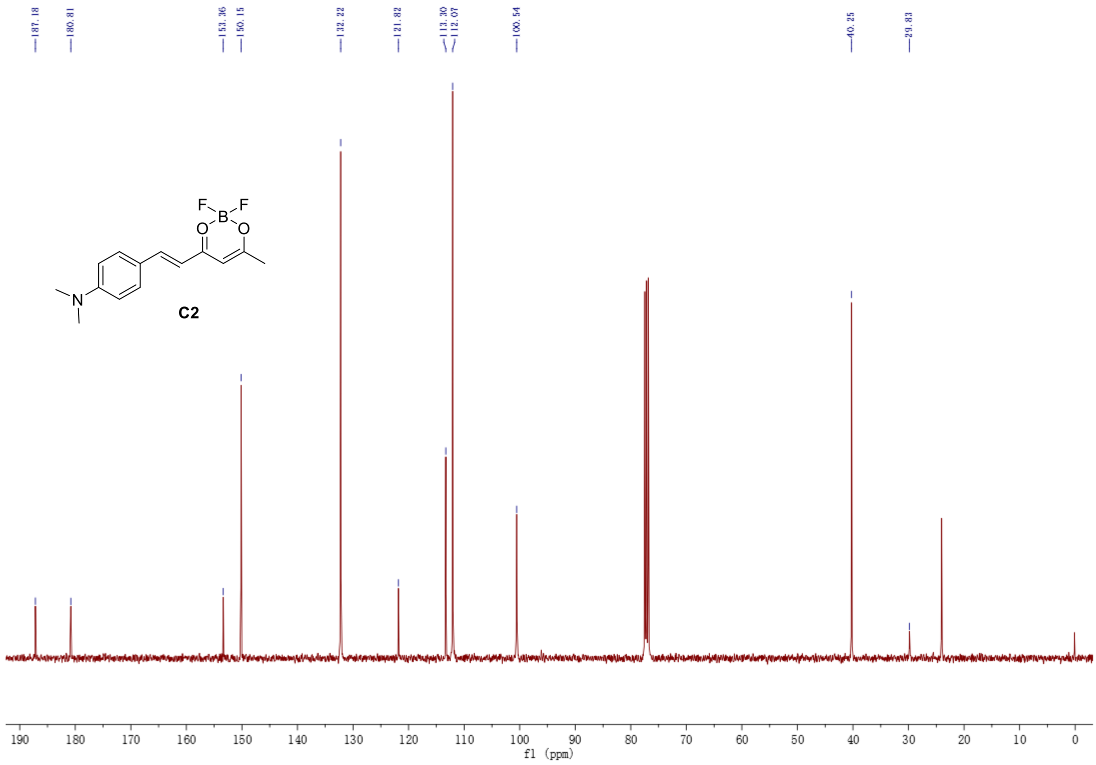

^{13}C-NMR spectra of **C2** (101 MHz, $CDCl_3$)

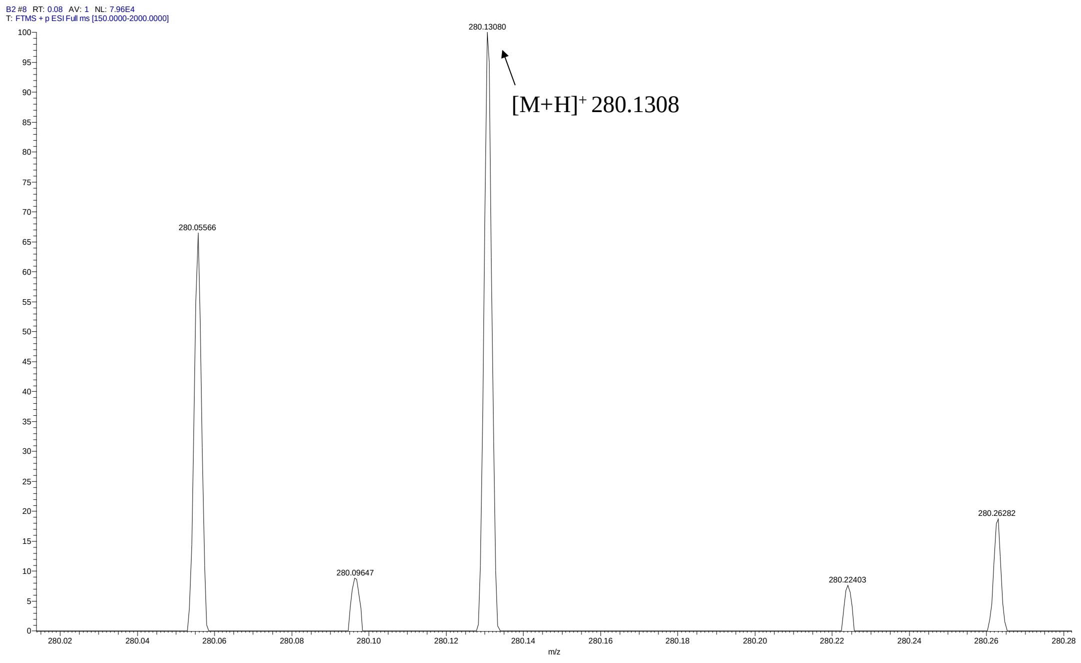

HRMS spectrum of **C2**

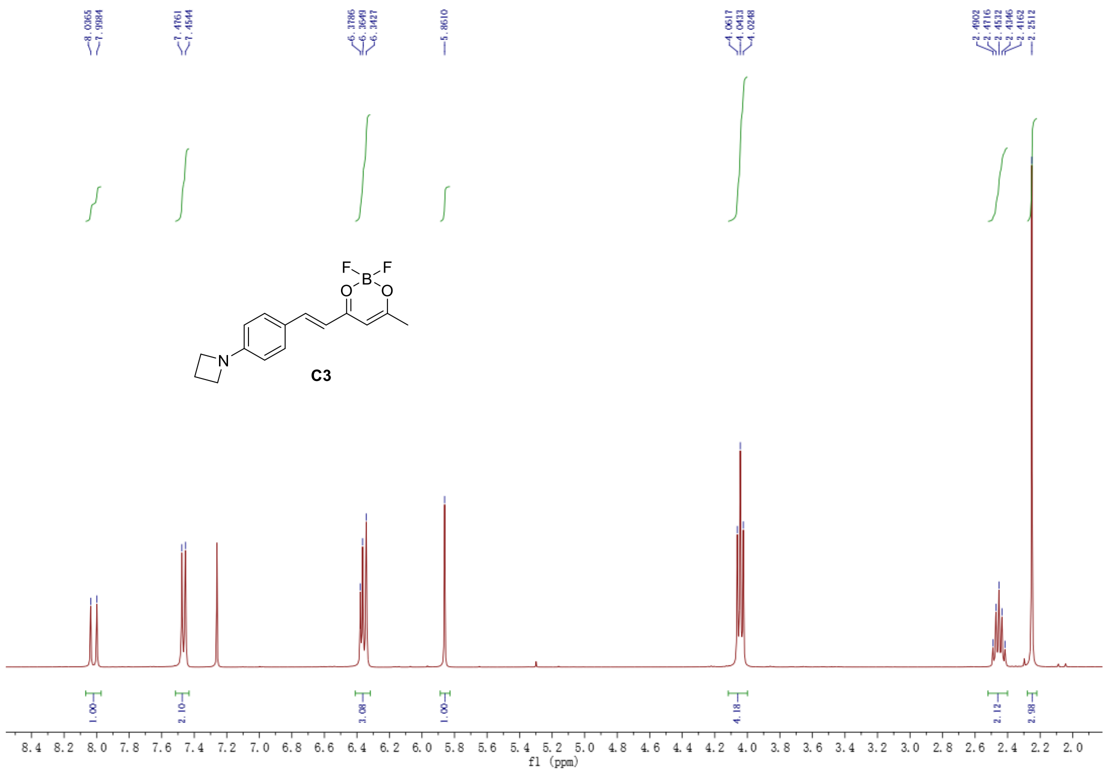

^{1}H-NMR spectra of **C3** (400 MHz, $CDCl_3$)

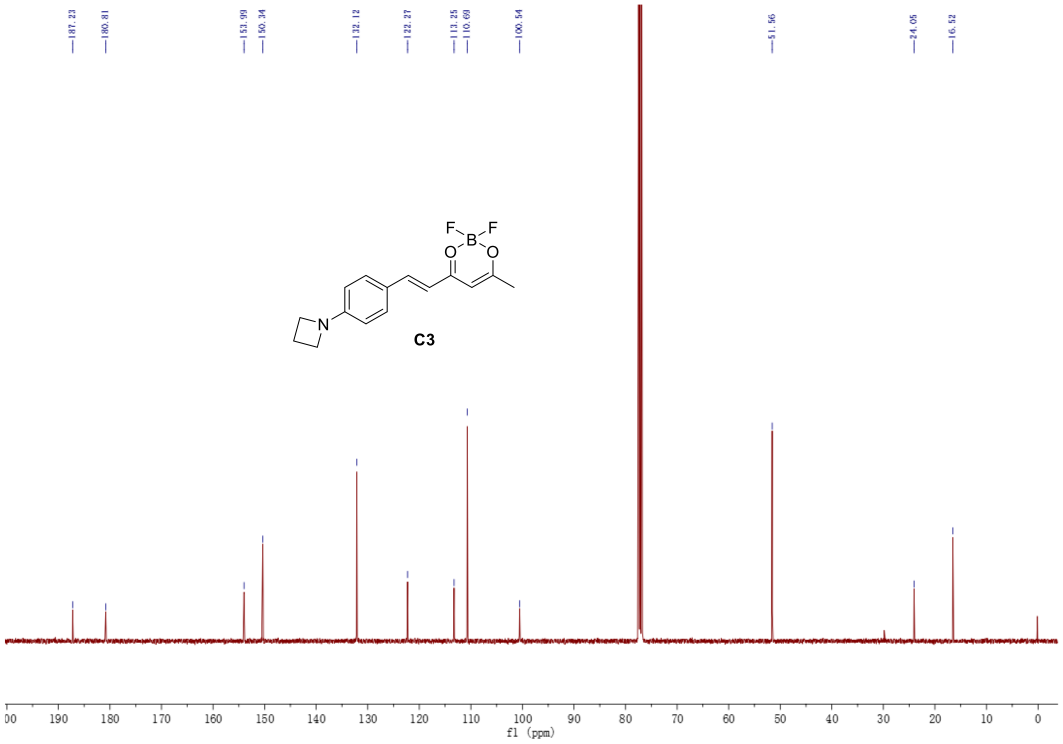

^{13}C-NMR spectra of **C3** (101 MHz, $CDCl_3$)

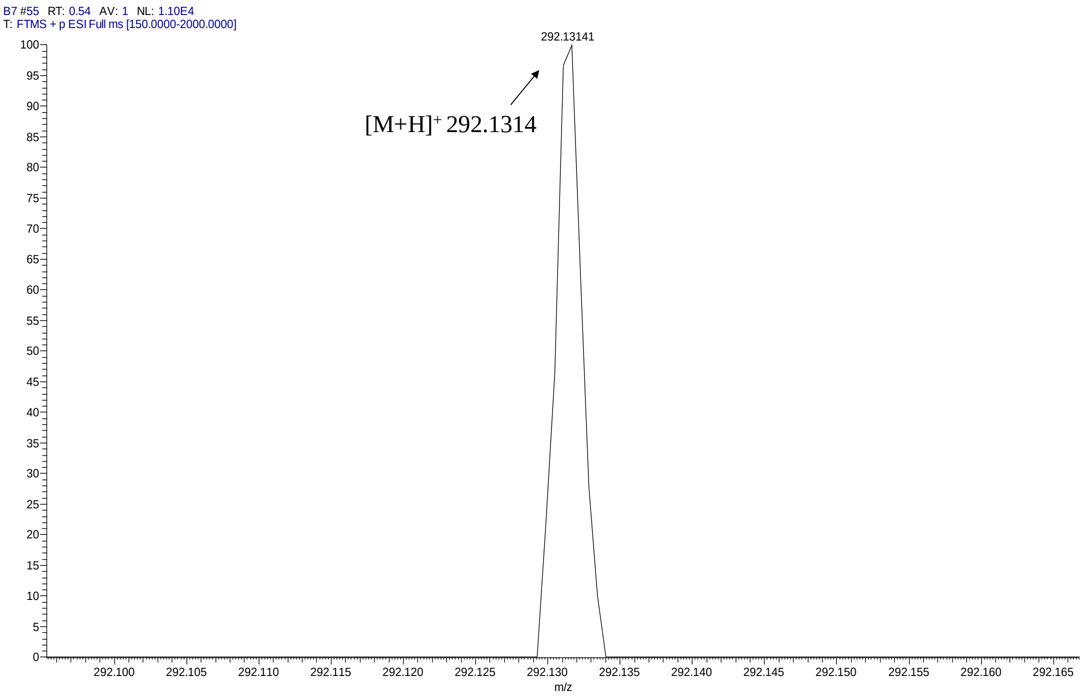

HRMS spectrum of **C3**

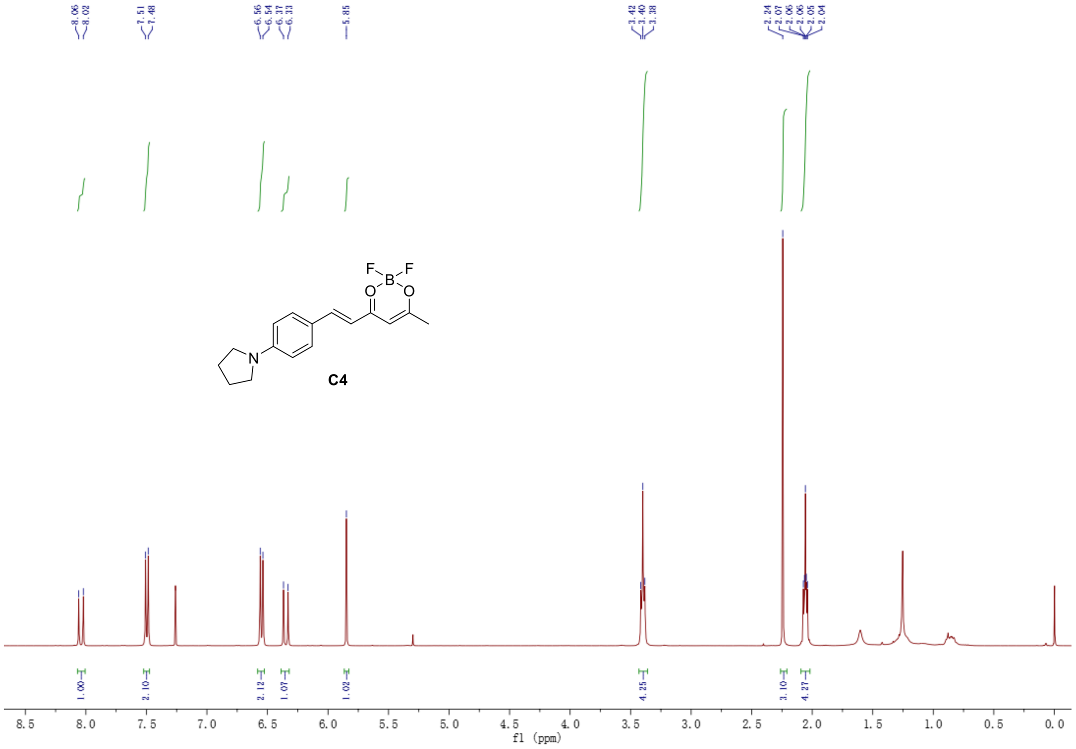

^{1}H-NMR spectra of **C4** (400 MHz, $CDCl_3$)

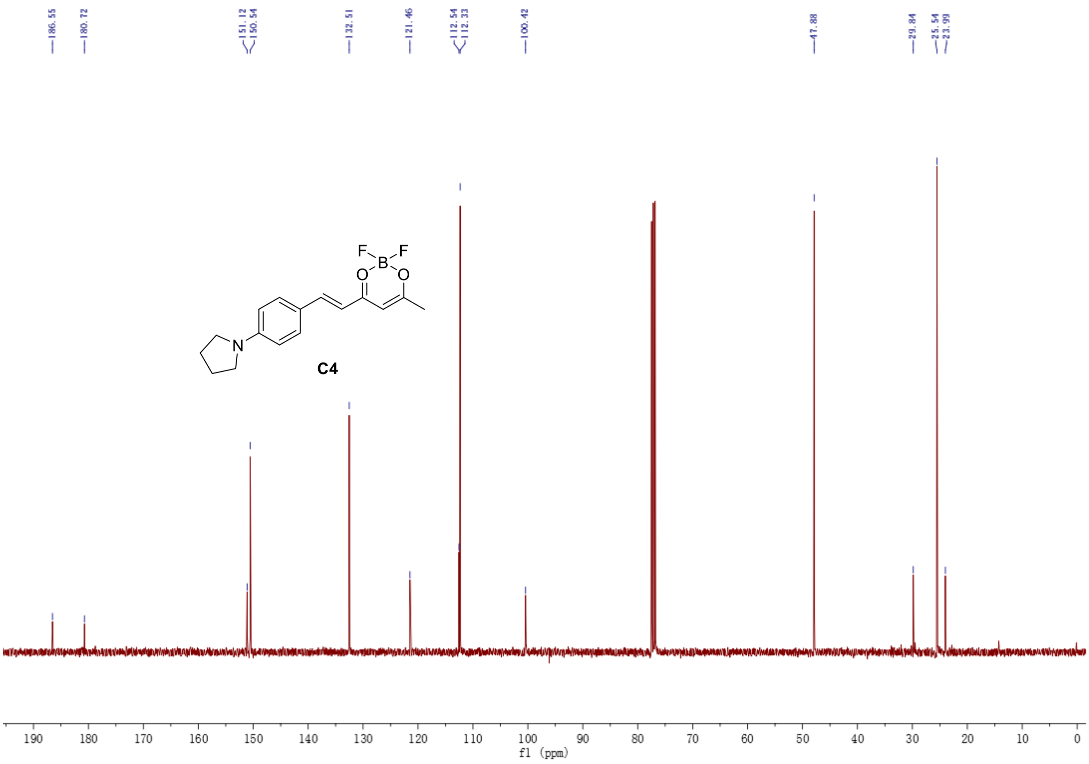

^{13}C-NMR spectra of **C4** (101 MHz, $CDCl_3$)

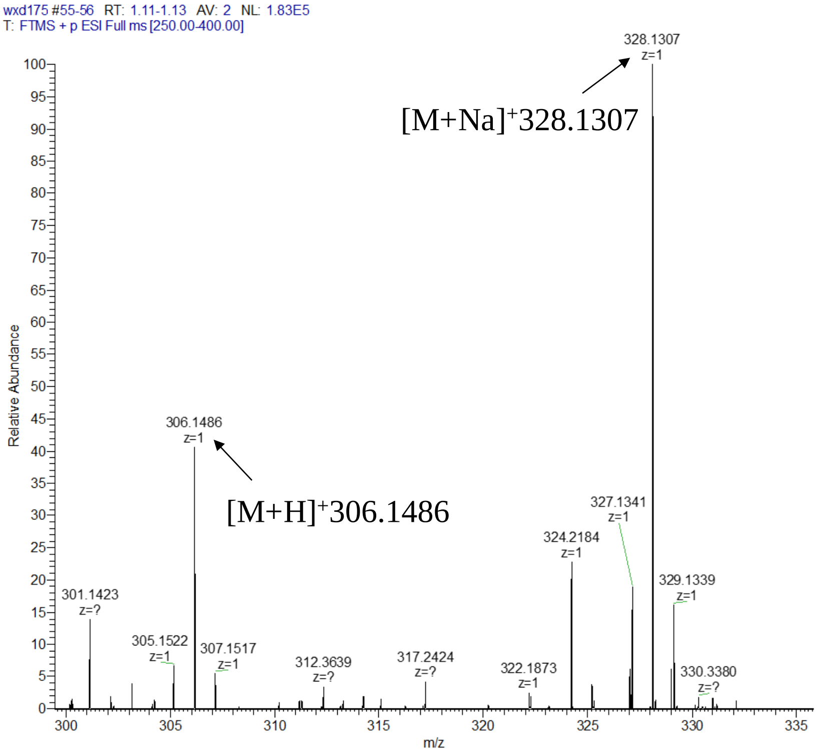

HRMS spectrum of **C4**

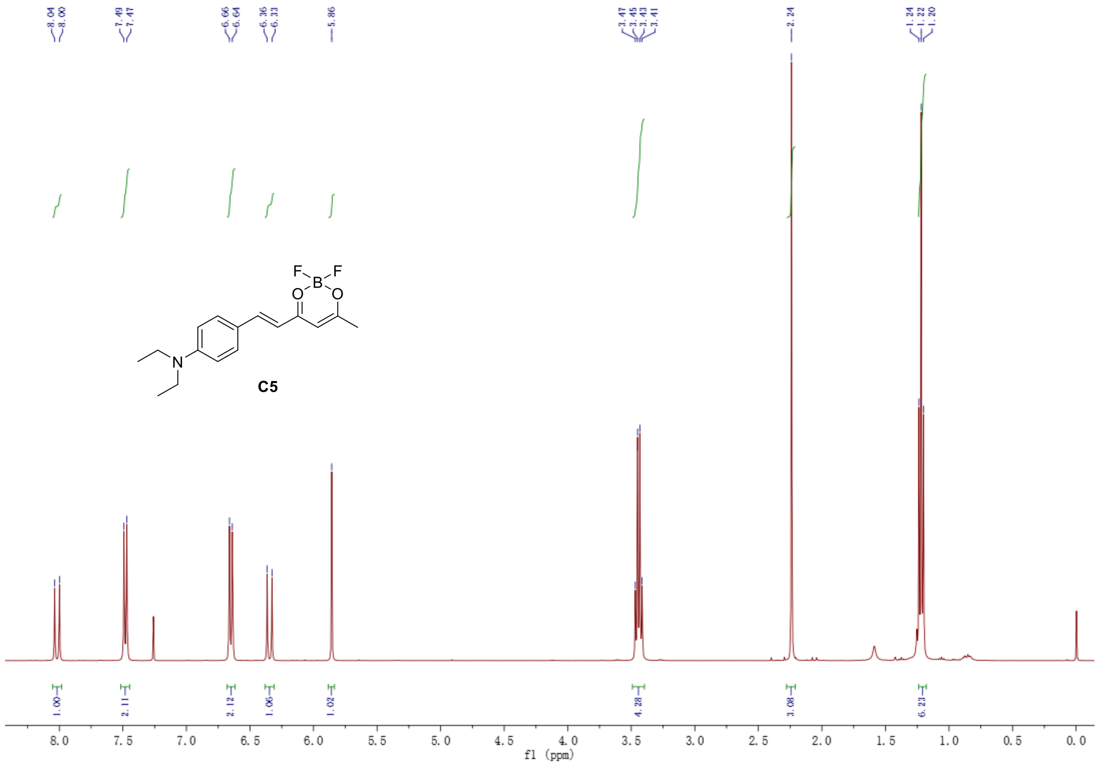

^{1}H-NMR spectra of **C5** (400 MHz, $CDCl_3$)

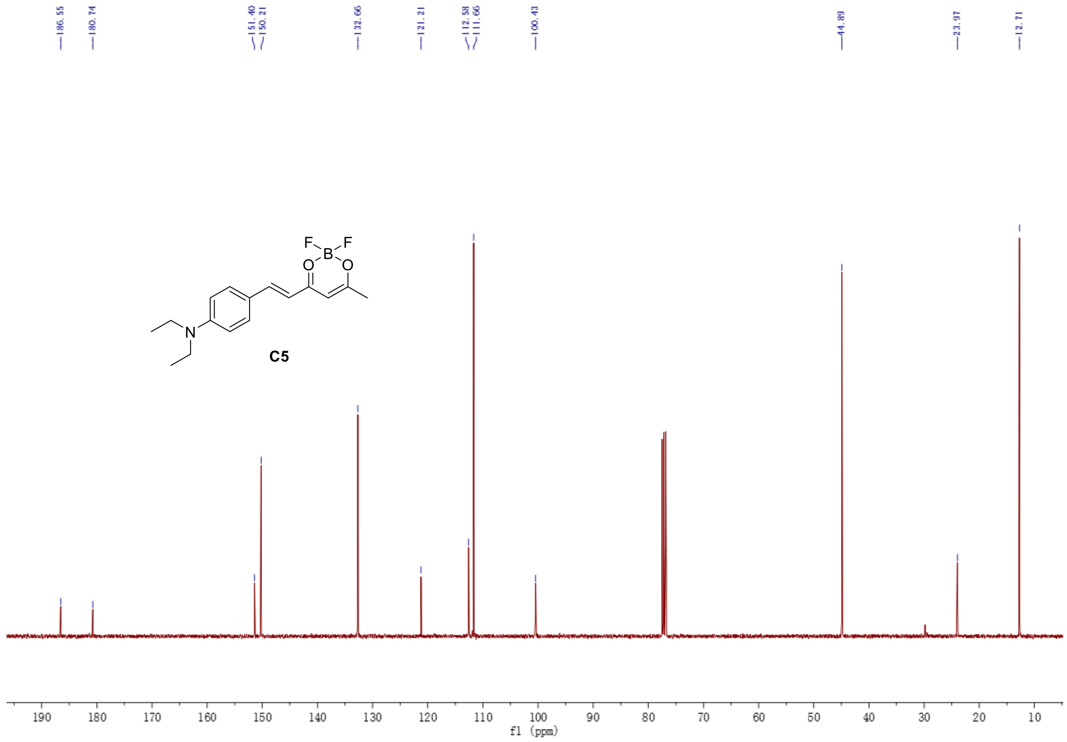

^{13}C-NMR spectra of **C5** (101 MHz, $CDCl_3$)

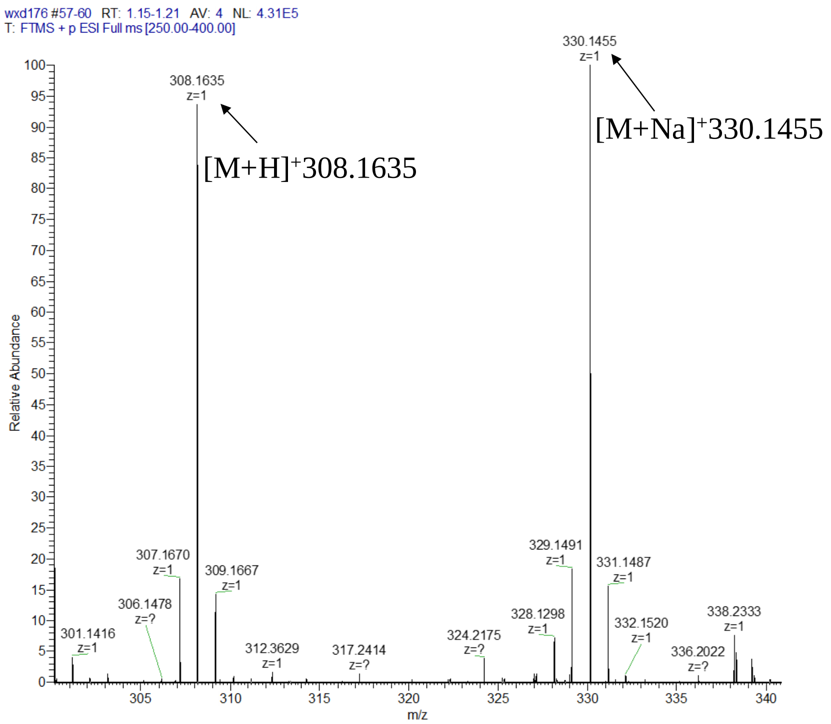

HRMS spectrum of **C5**

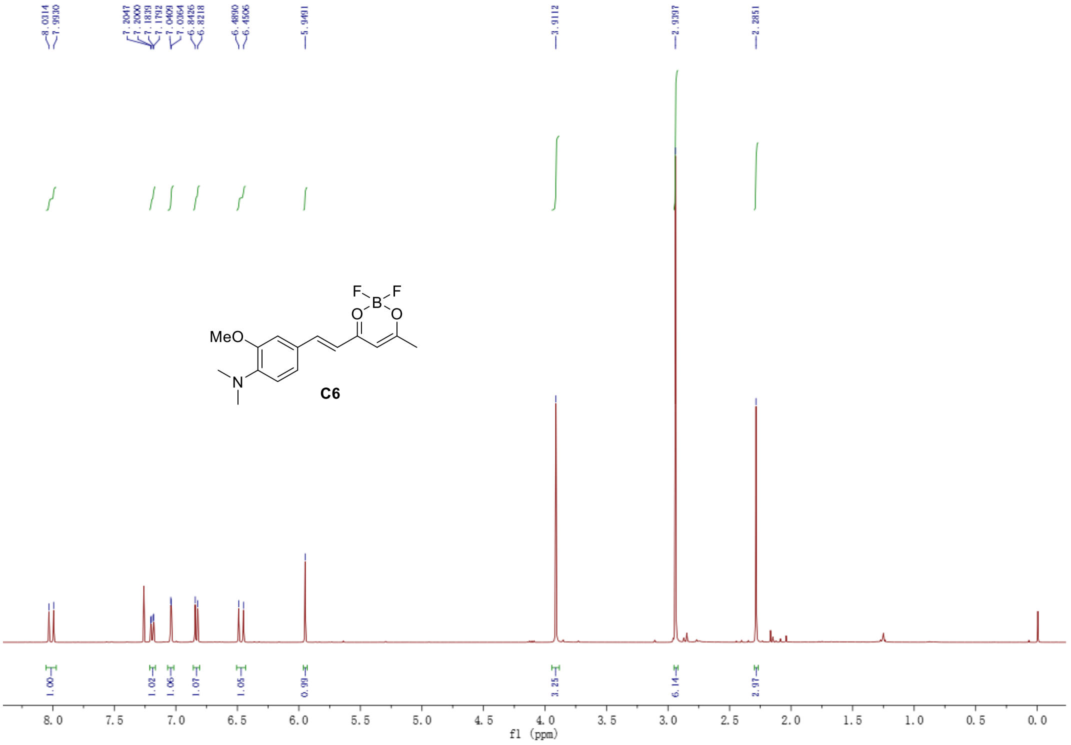

^{1}H-NMR spectra of **C6** (400 MHz, $CDCl_3$)

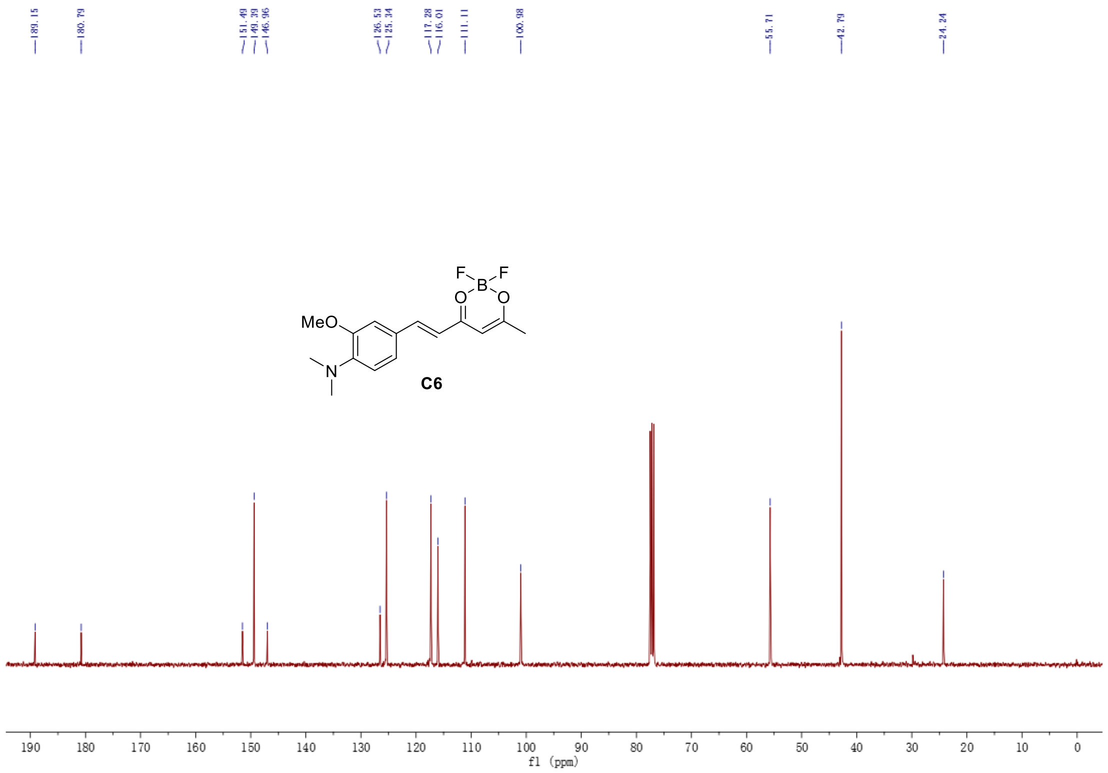

^{13}C-NMR spectra of **C6** (101 MHz, $CDCl_3$)

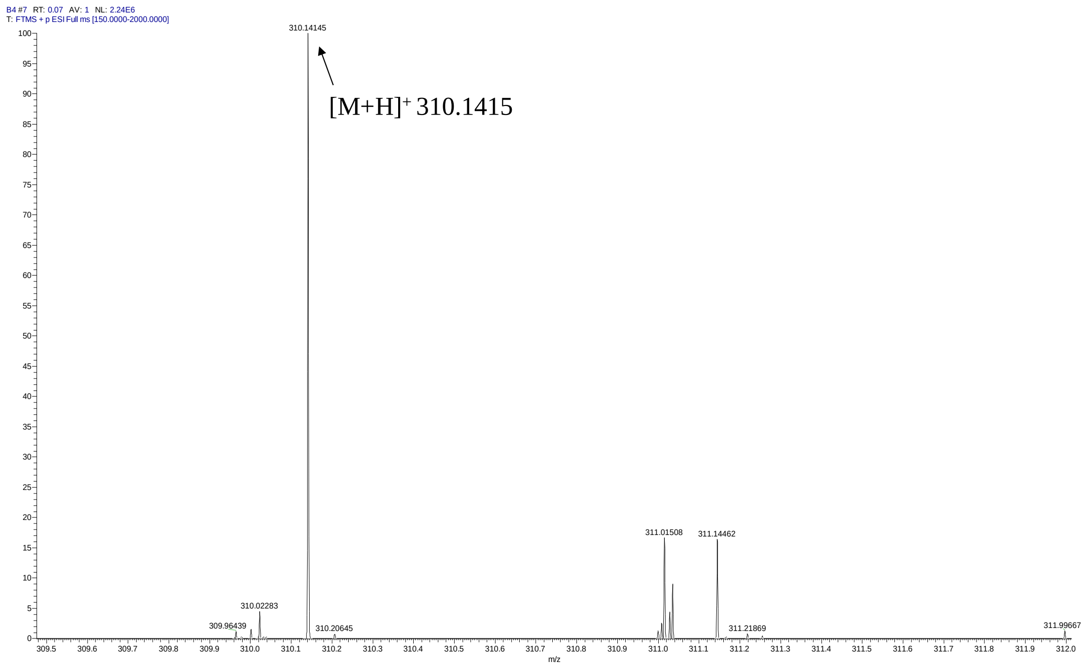

HRMS spectrum of **C6**

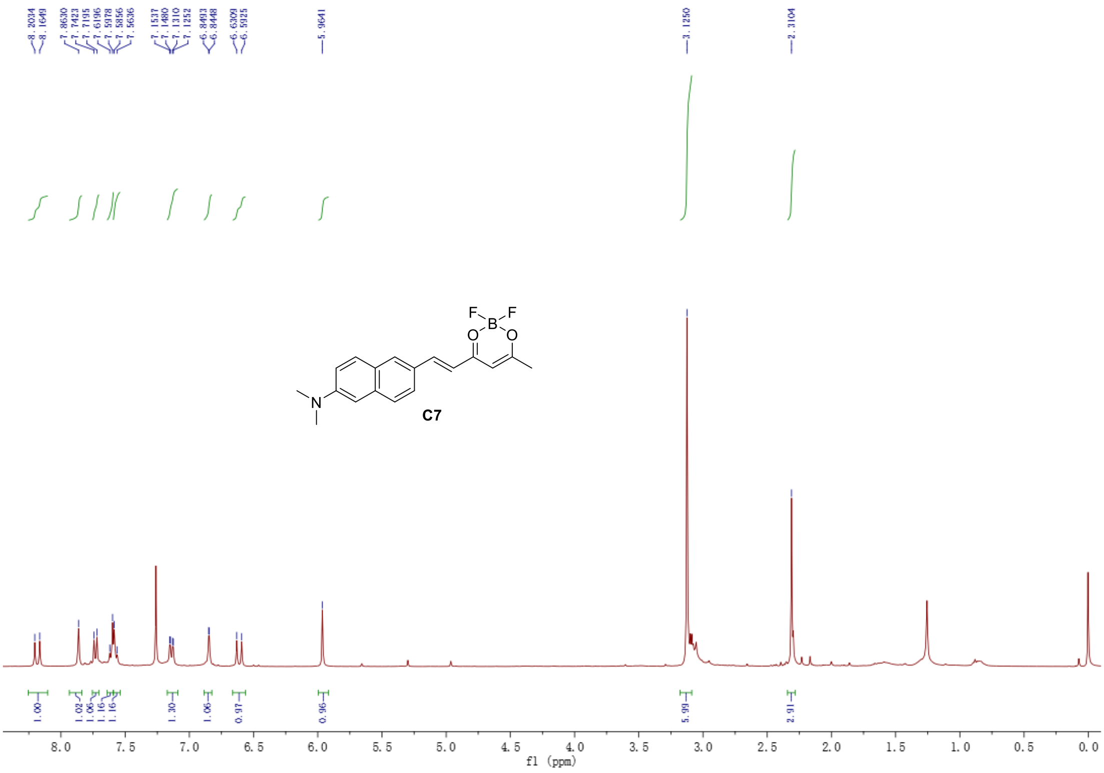

^{1}H-NMR spectra of **C7** (400 MHz, $CDCl_3$)

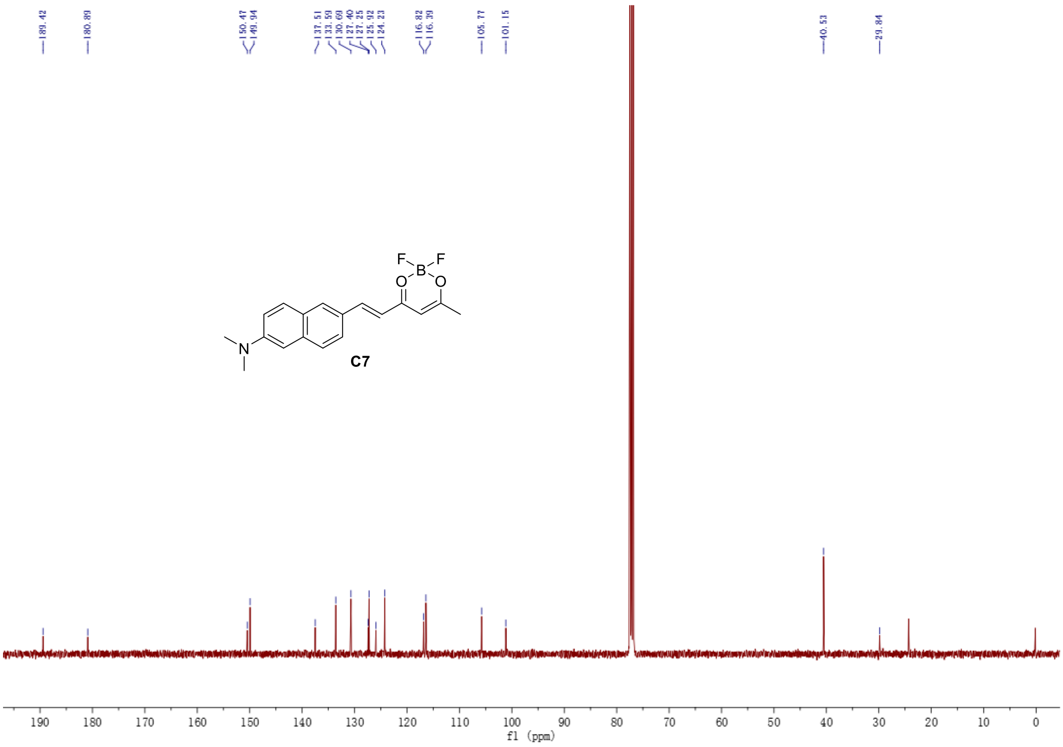

^{13}C-NMR spectra of **C7** (101 MHz, $CDCl_3$)

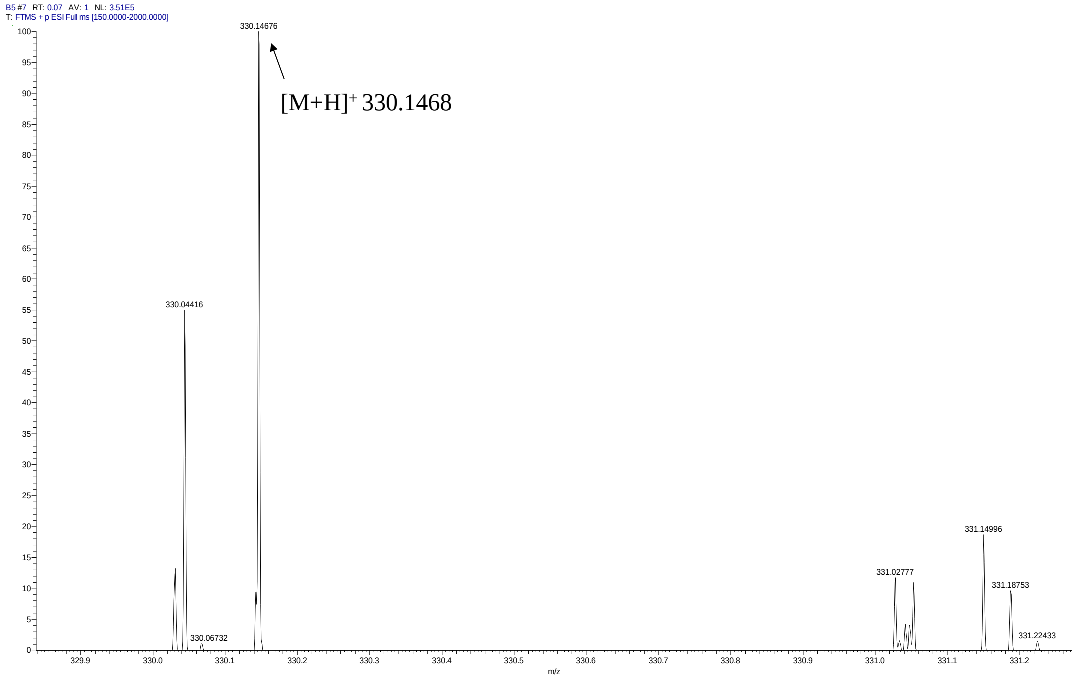

HRMS spectrum of **C7**

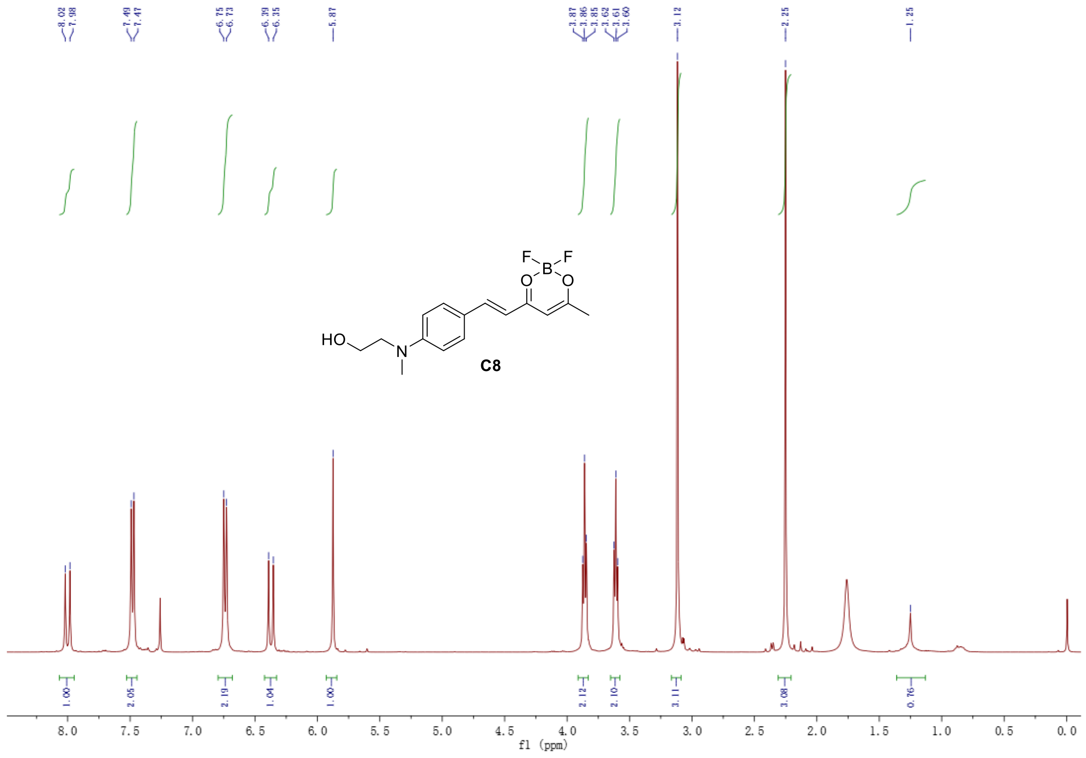

^{1}H-NMR spectra of **C8** (400 MHz, $CDCl_3$)

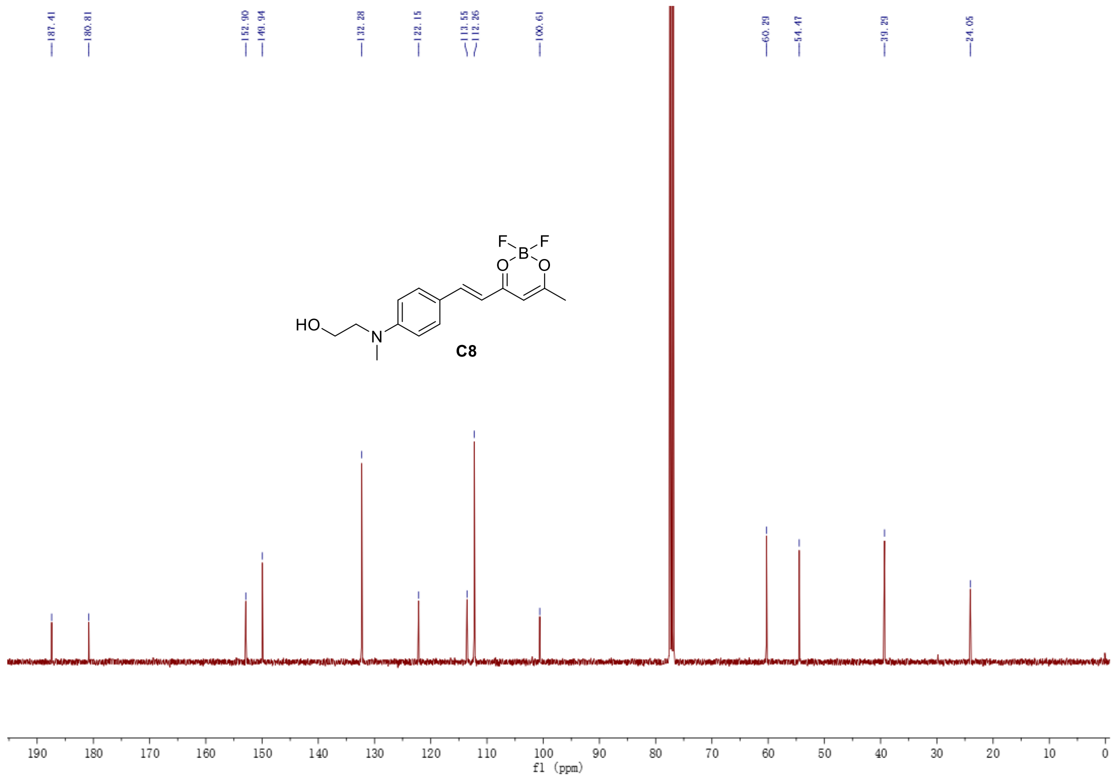

^{13}C-NMR spectra of **C8** (101 MHz, $CDCl_3$)

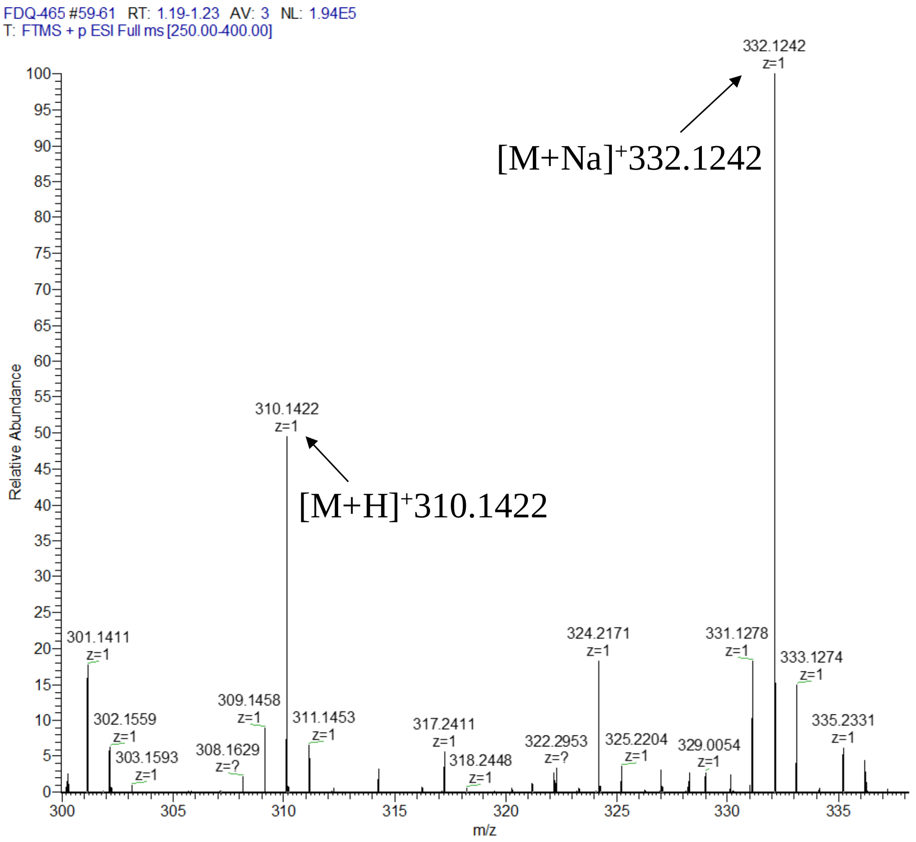

HRMS spectrum of **C8**

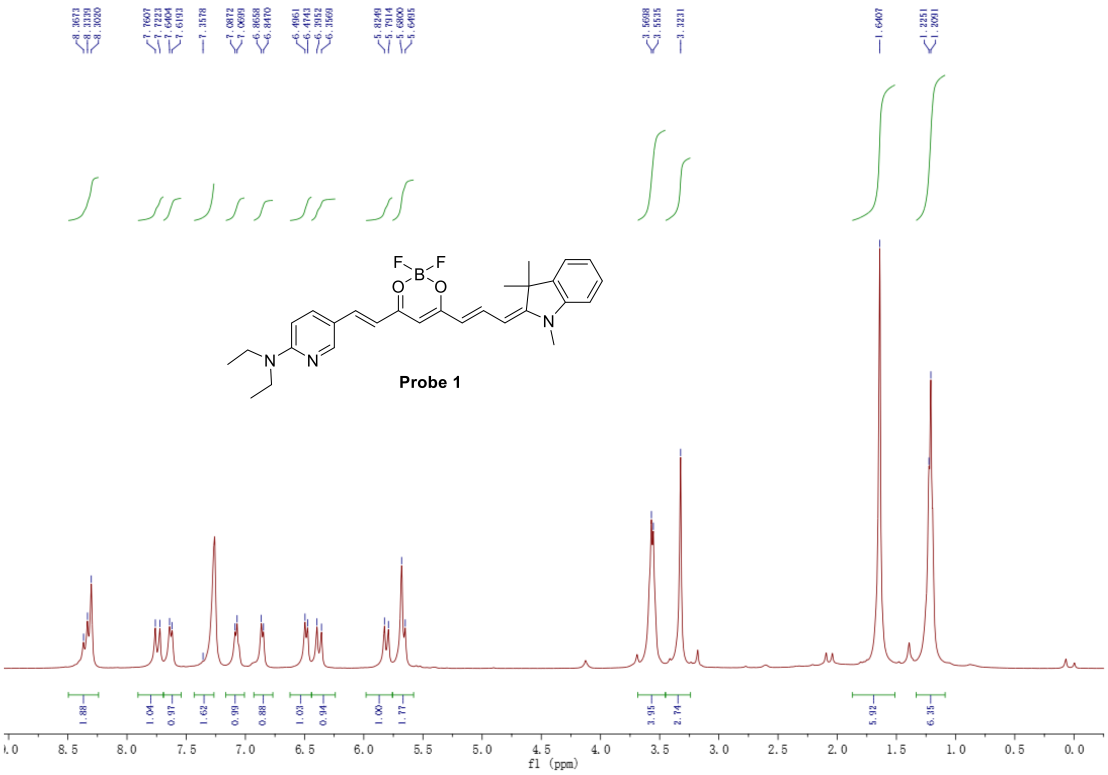

^{1}H-NMR spectra of probe **1** (400 MHz, $CDCl_3$)

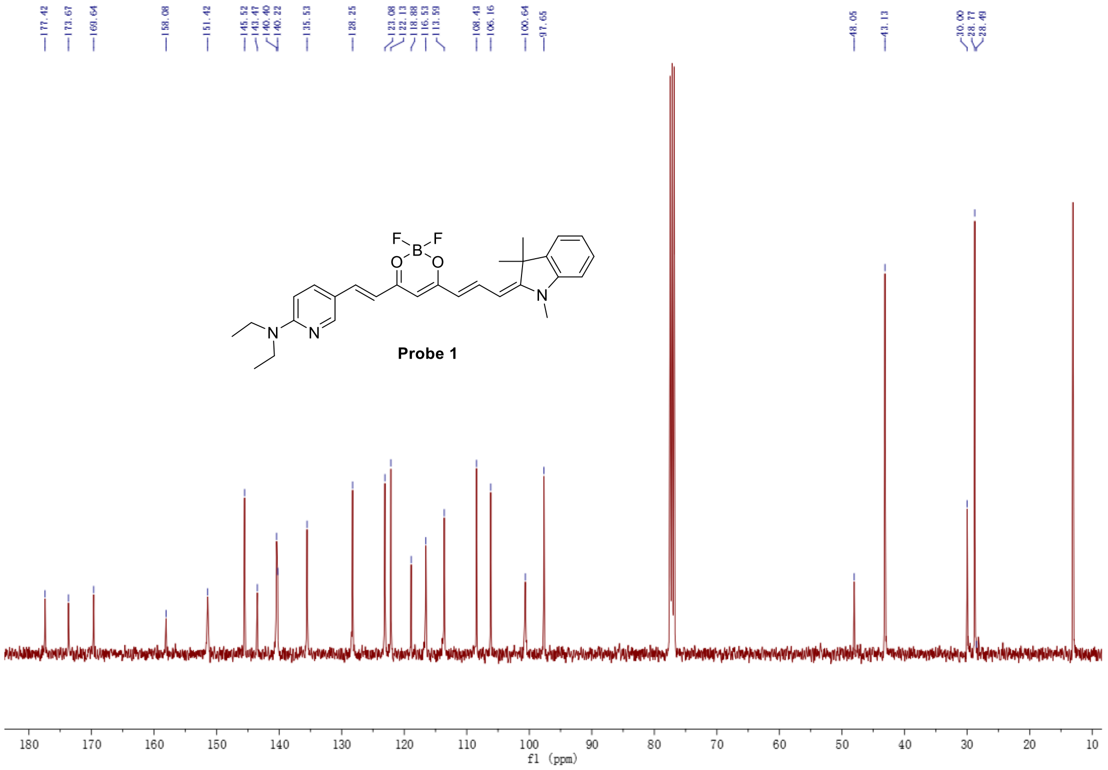

^{13}C-NMR spectra of probe **1** (101 MHz, $CDCl_3$)

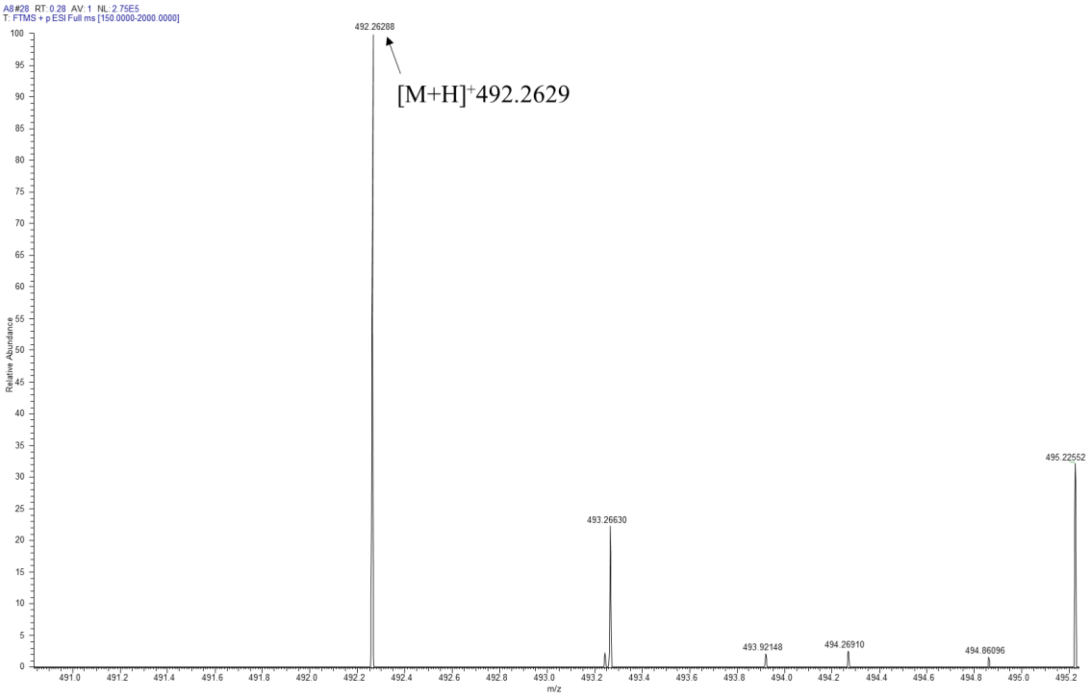

HRMS Spectrum of probe **1**

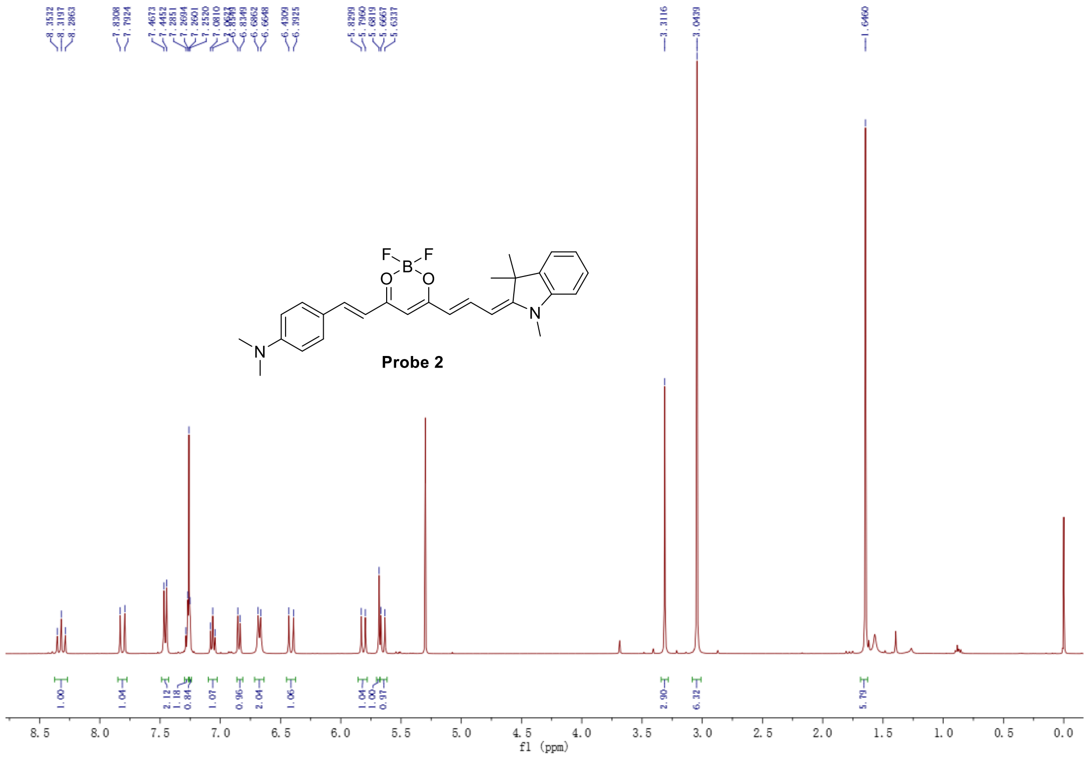

^{1}H-NMR spectra of probe **2** (400 MHz, $CDCl_3$)

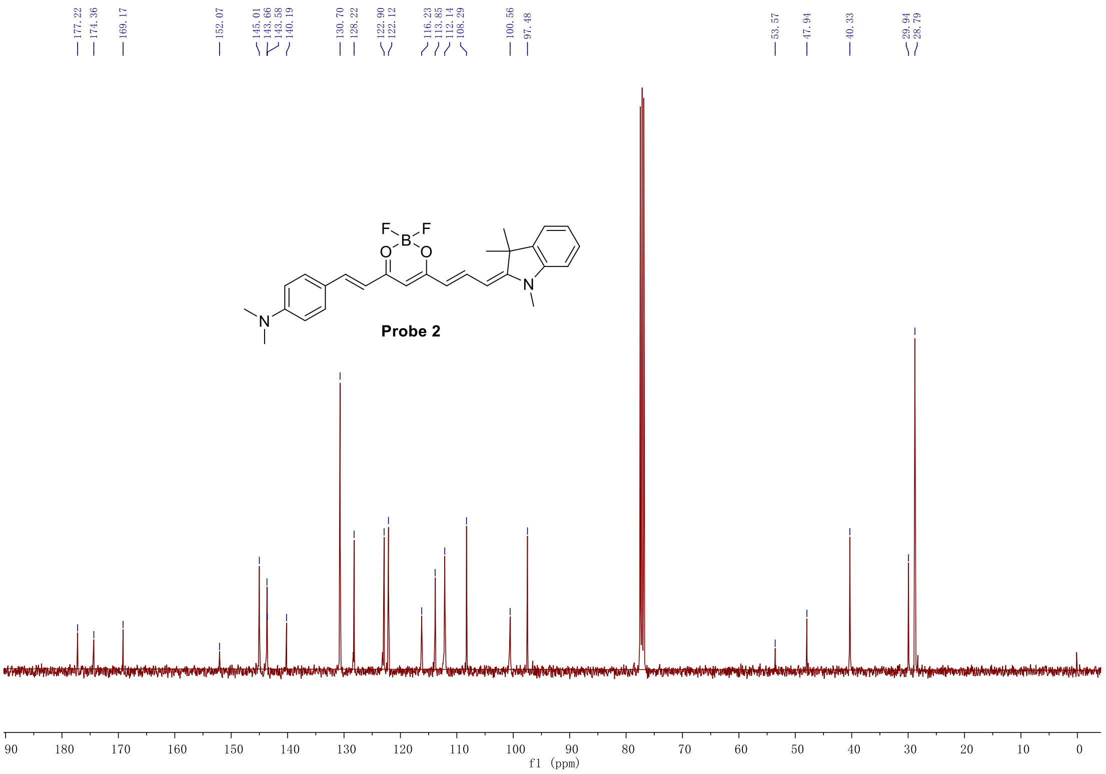

^{13}C-NMR spectra of probe **2** (101 MHz, $CDCl_3$)

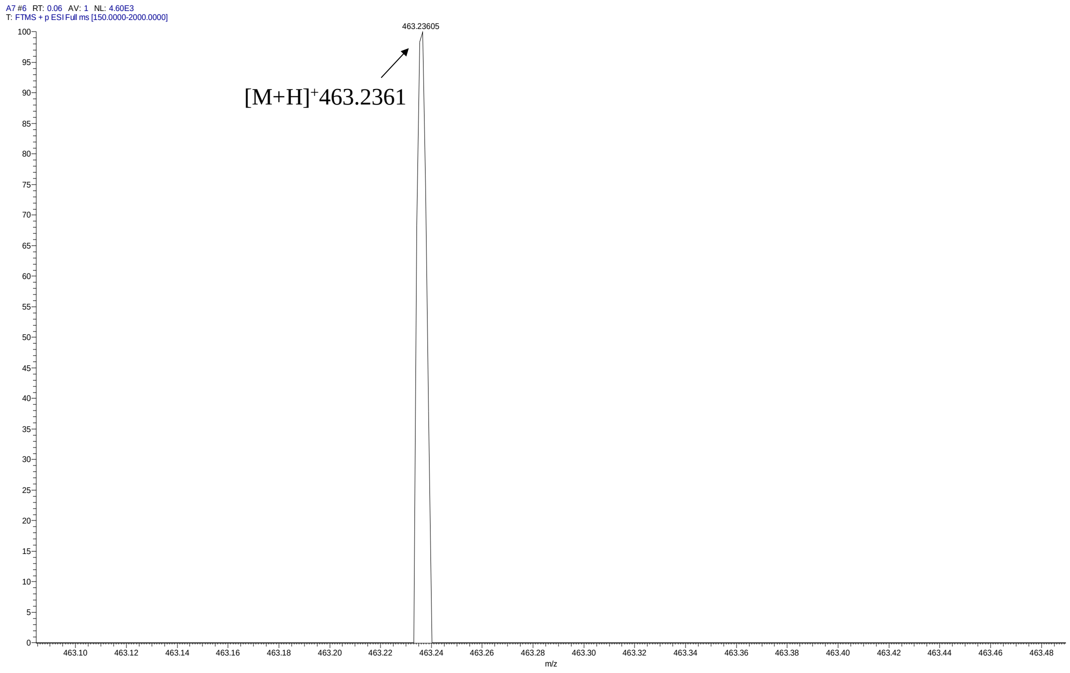

HRMS Spectrum of probe **2**

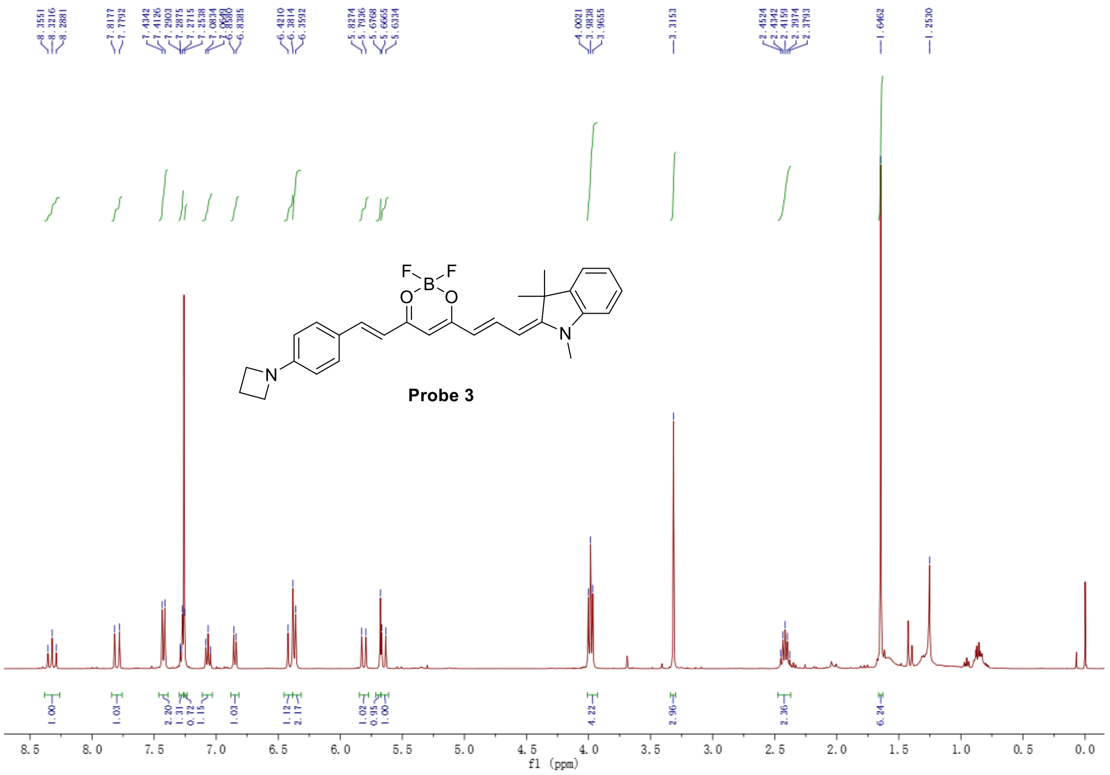

^{1}H-NMR spectra of probe **3** (400 MHz, $CDCl_3$)

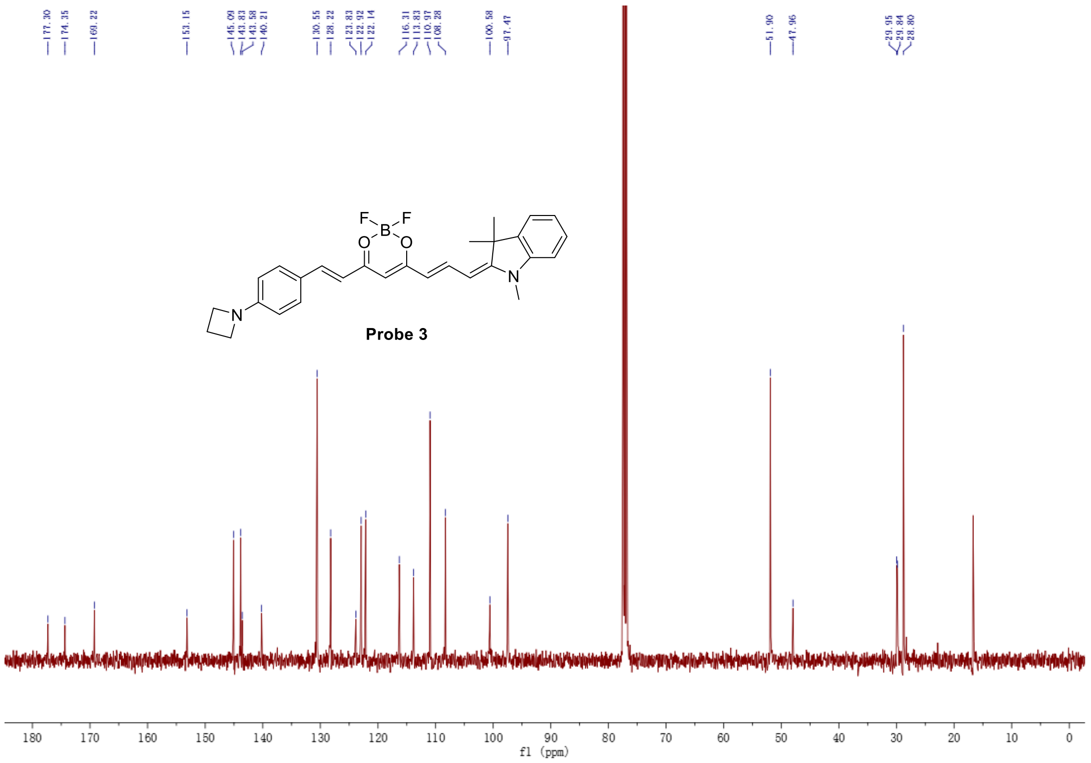

^{13}C-NMR spectra of probe **3** (101 MHz, $CDCl_3$)

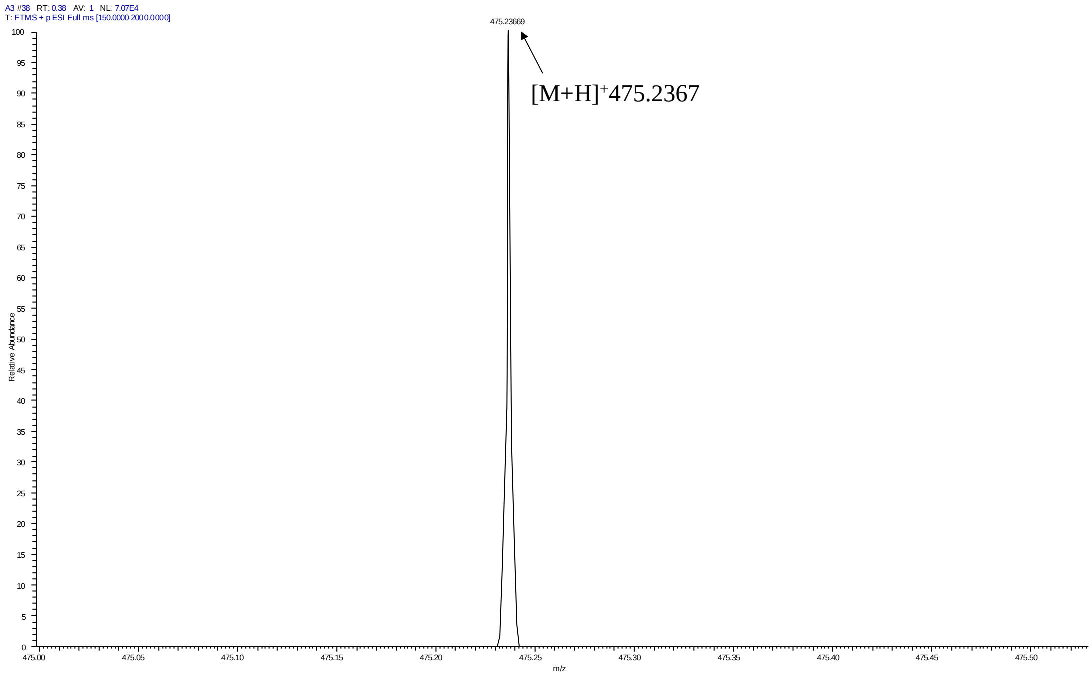

HRMS Spectrum of probe **3**

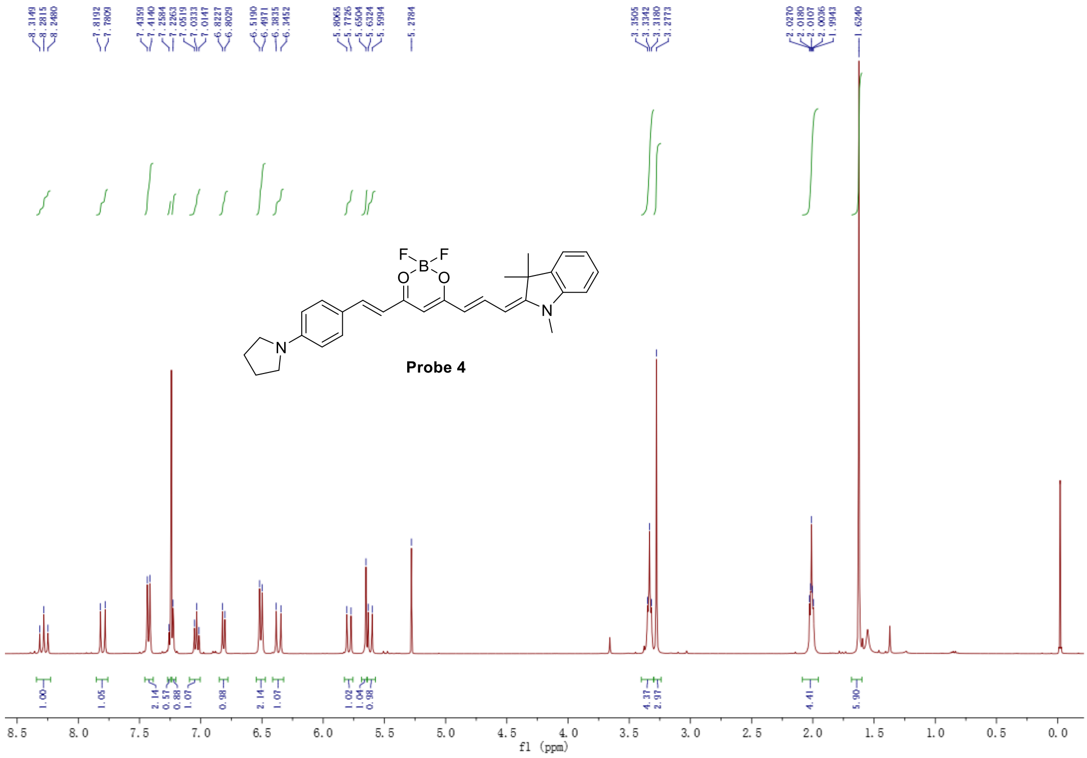

^{1}H-NMR spectra of probe **4** (400 MHz, $CDCl_3$)

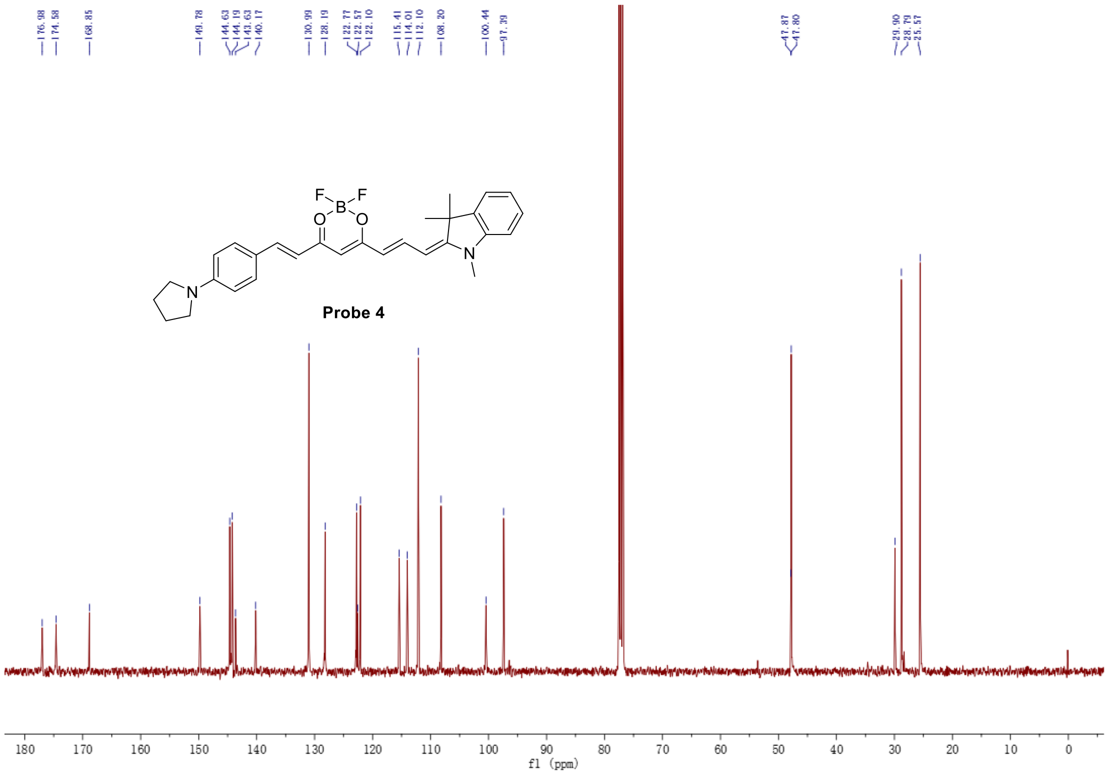

^{13}C-NMR spectra of probe **4** (101 MHz, $CDCl_3$)

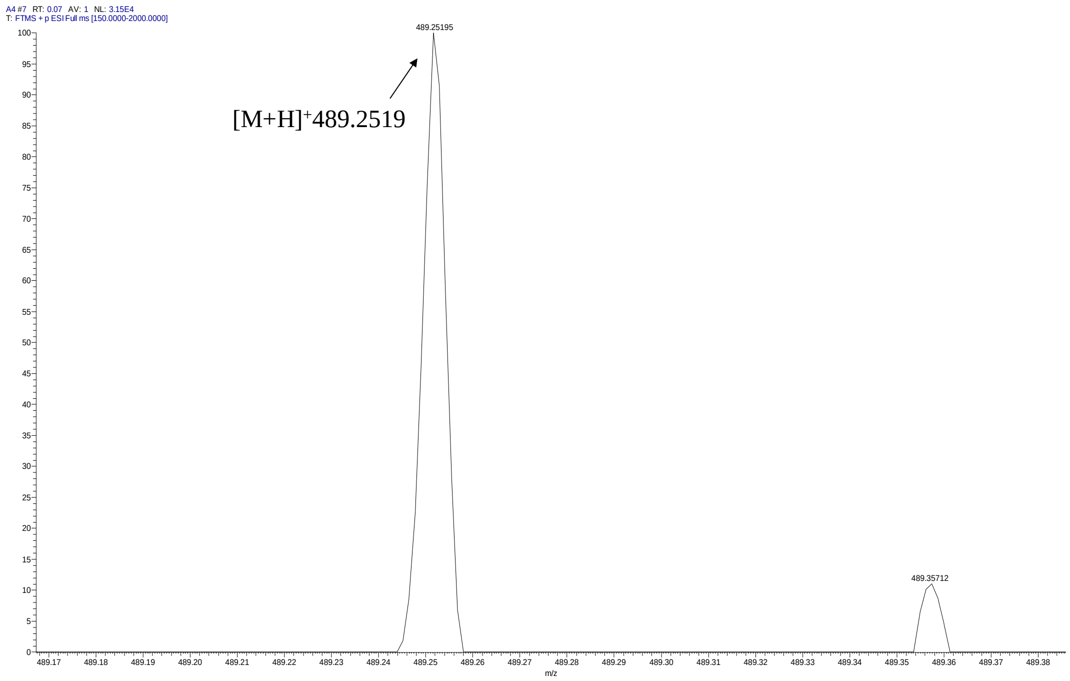

HRMS Spectrum of probe **4**

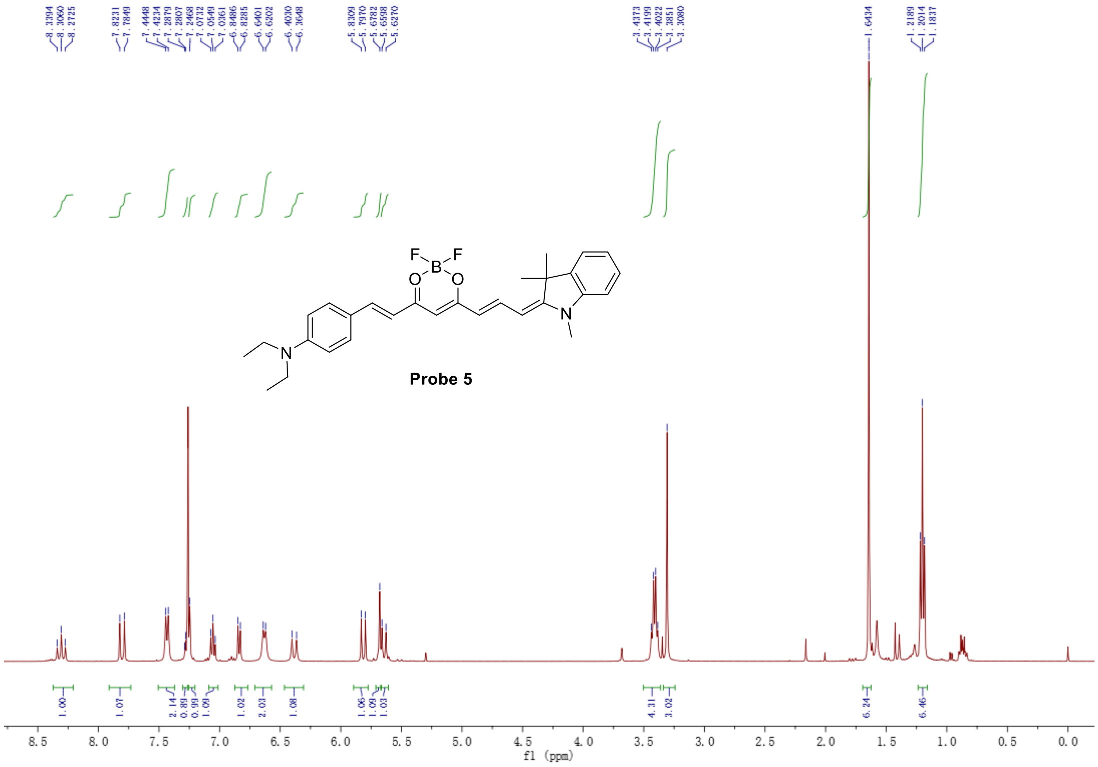

^{1}H-NMR spectra of probe **5** (400 MHz, $CDCl_3$)

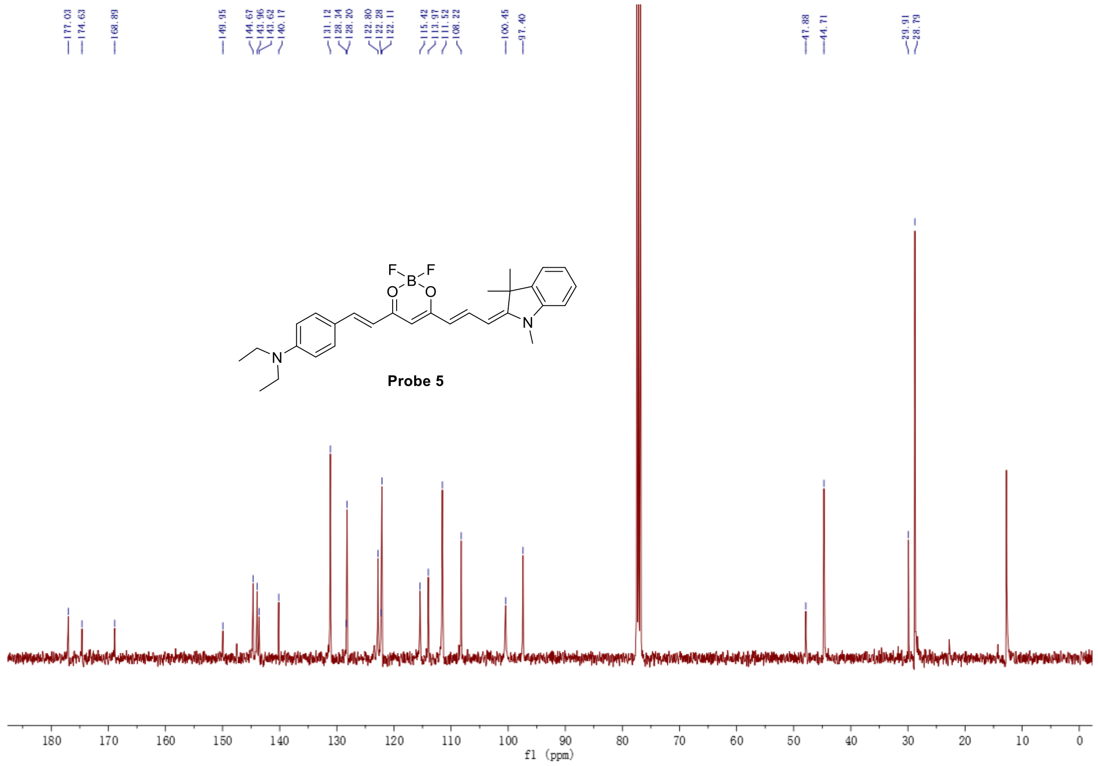

^{13}C-NMR spectra of probe **5** (101 MHz, $CDCl_3$)

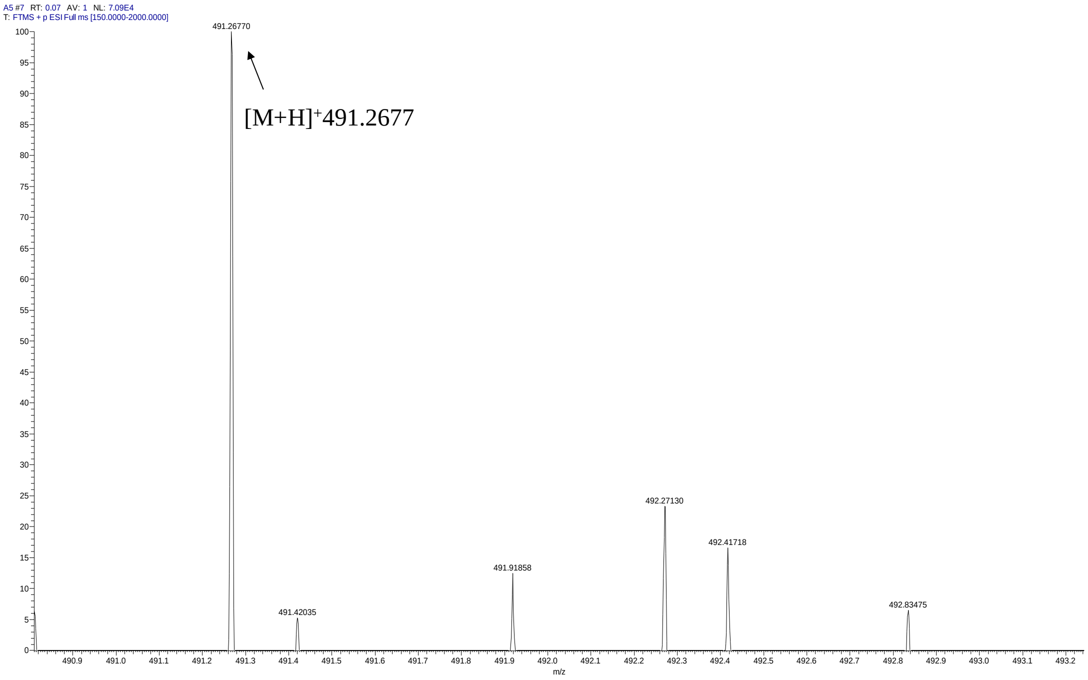

HRMS Spectrum of probe **5**

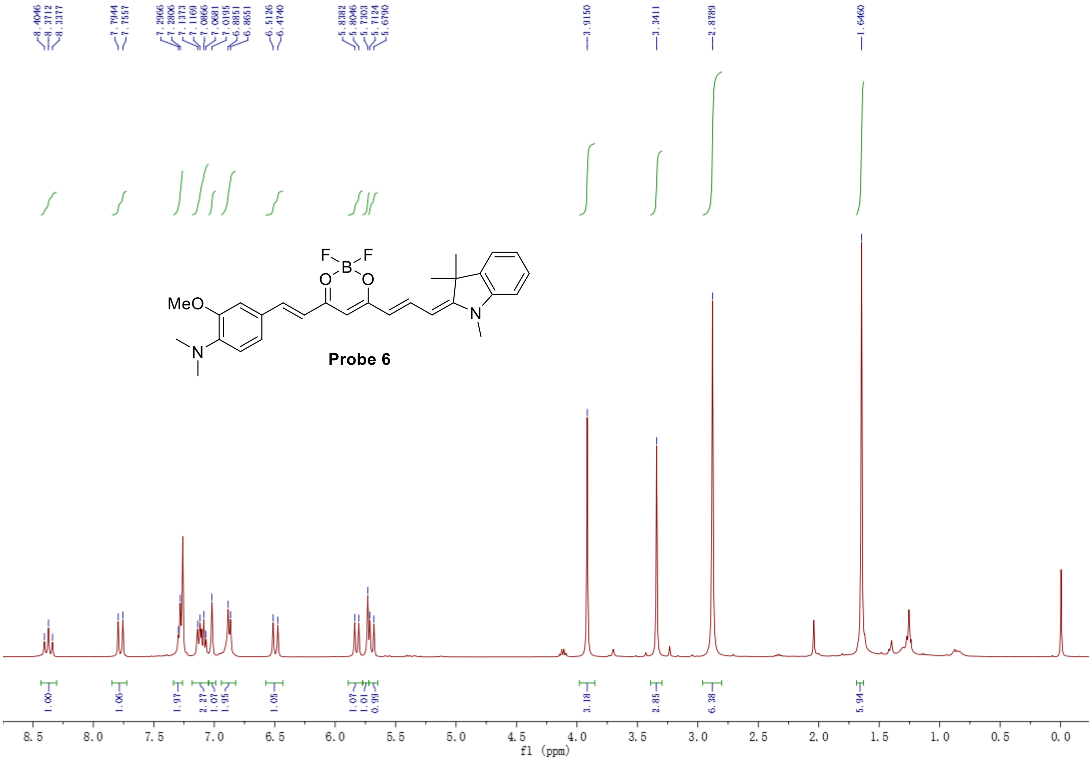

^{1}H-NMR spectra of probe **6** (400 MHz, $CDCl_3$)

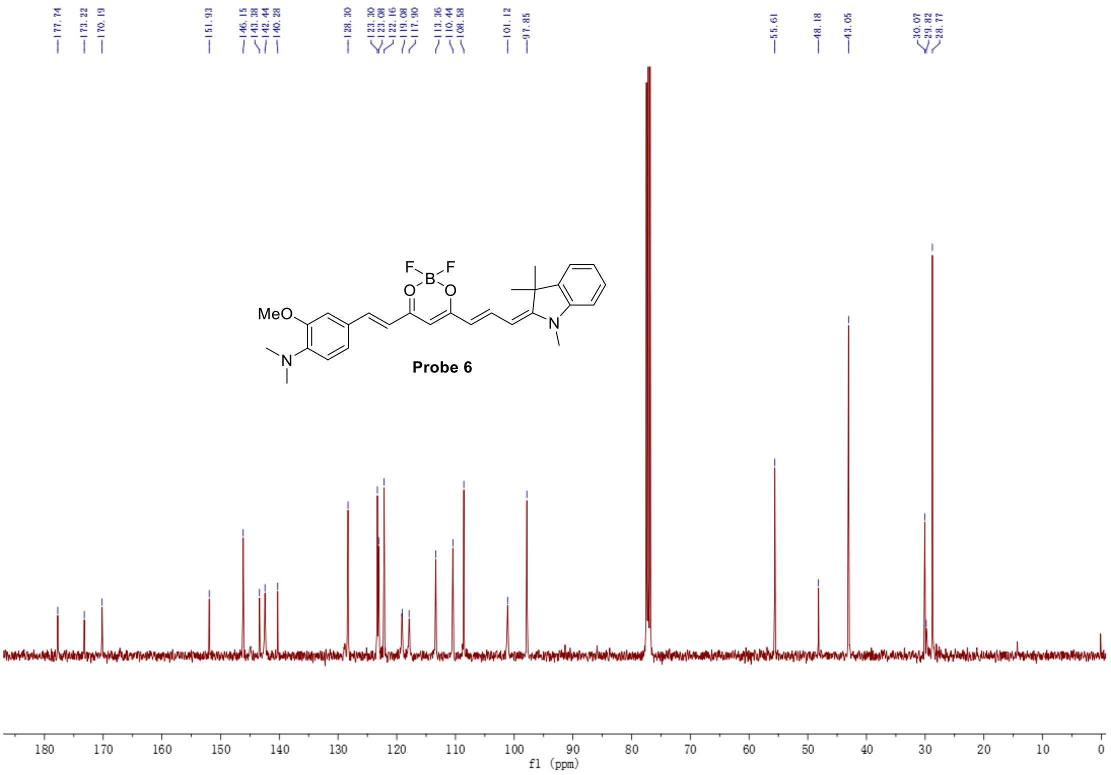

^{13}C-NMR spectra of probe **6** (101 MHz, $CDCl_3$)

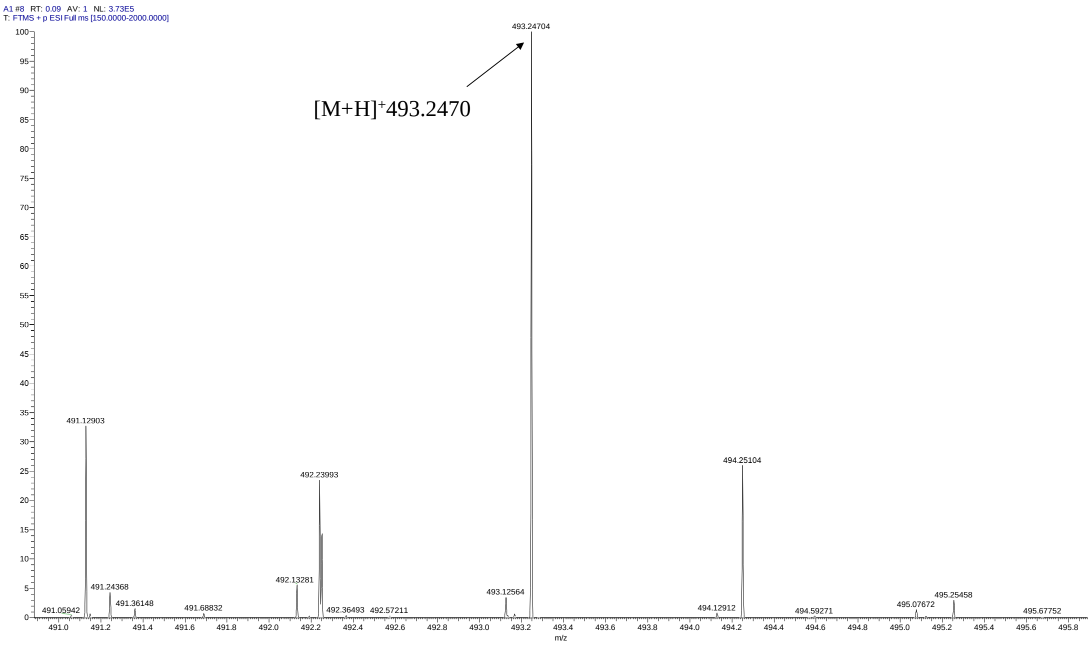

HRMS Spectrum of probe **6**

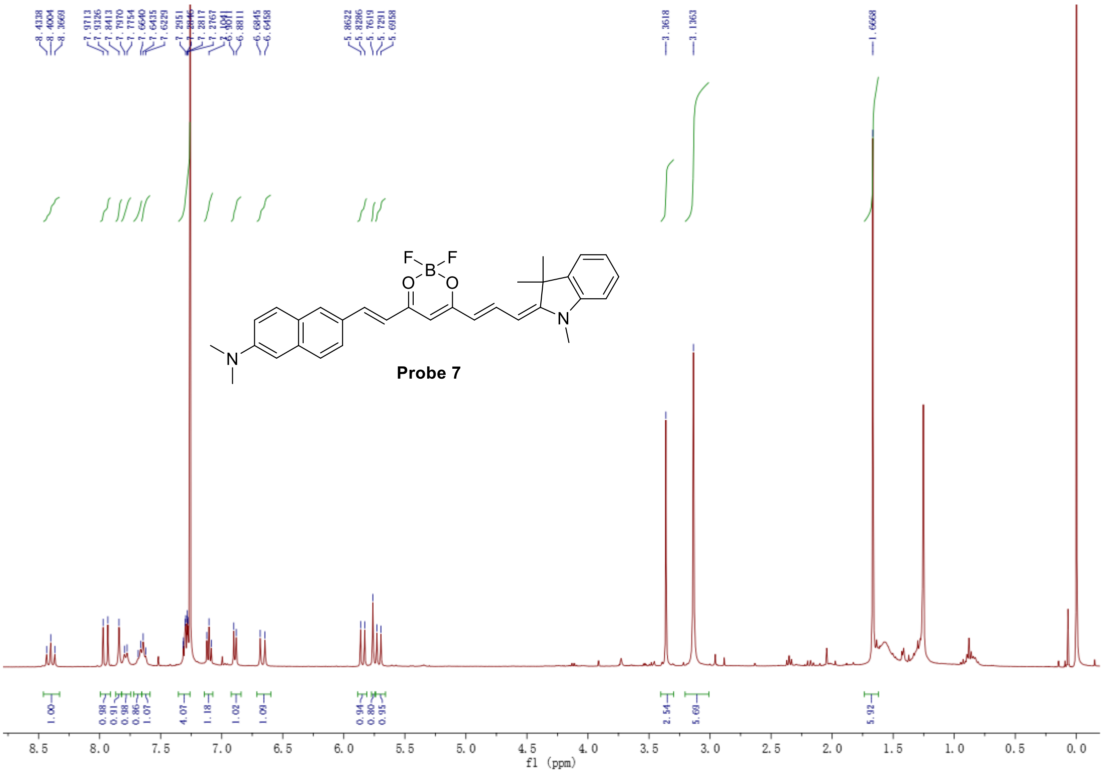

^{1}H-NMR spectra of probe **7** (400 MHz, $CDCl_3$)

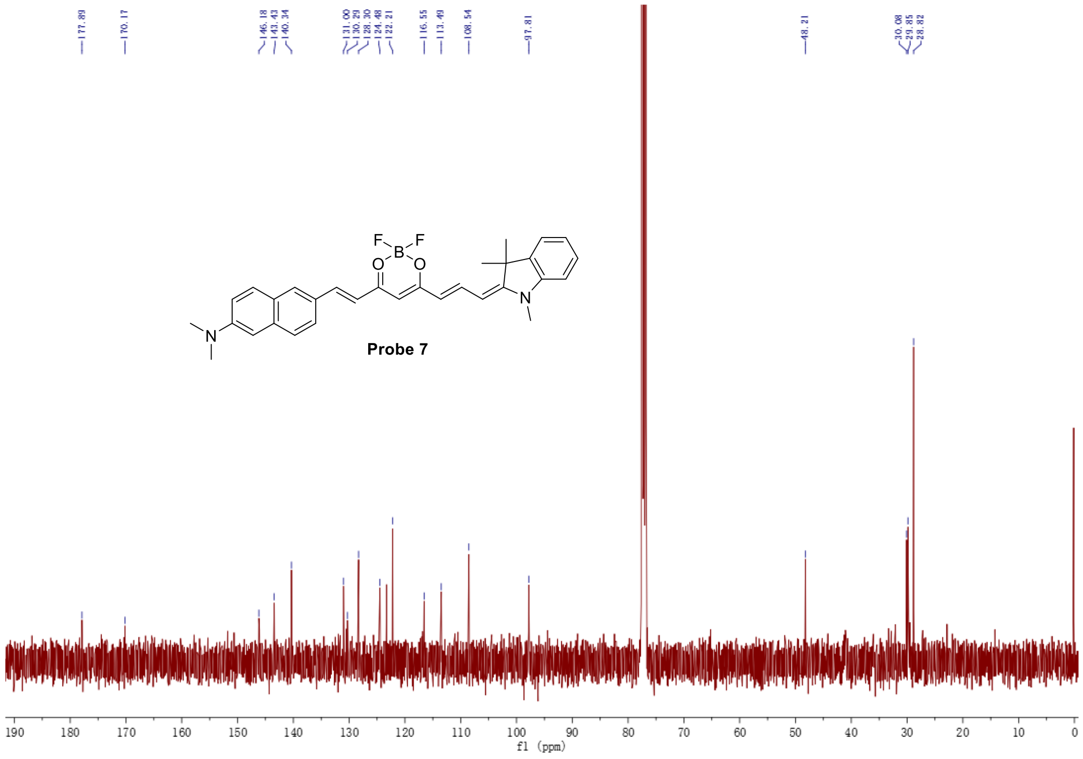

^{13}C-NMR spectra of probe **7** (101 MHz, $CDCl_3$)

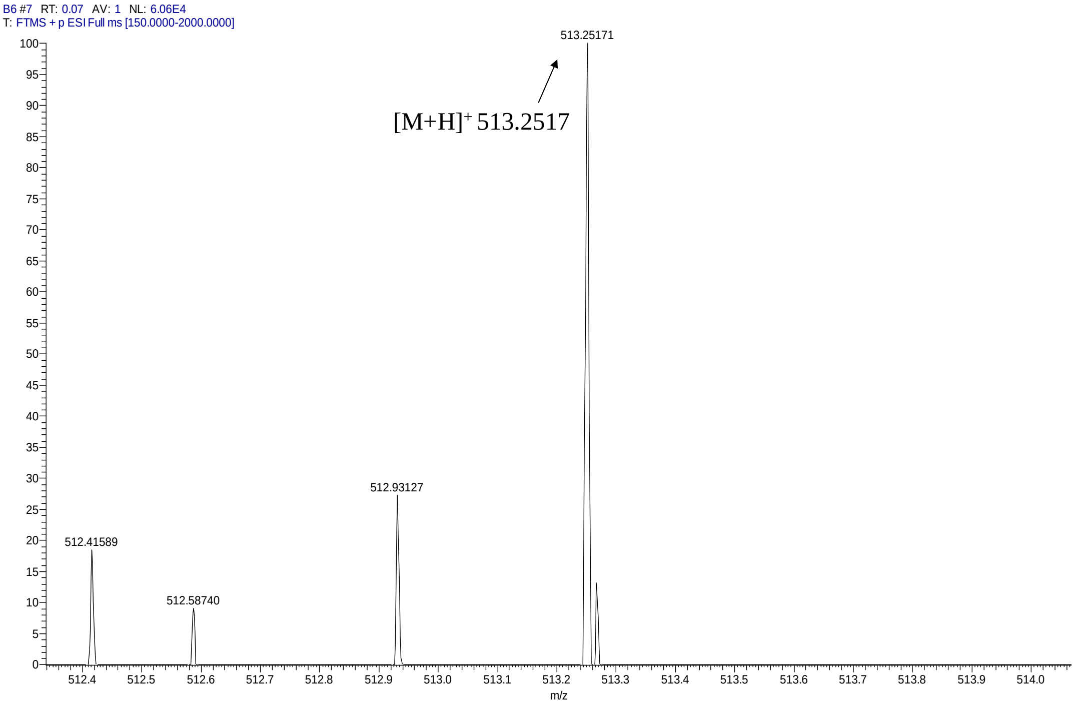

HRMS Spectrum of probe **7**

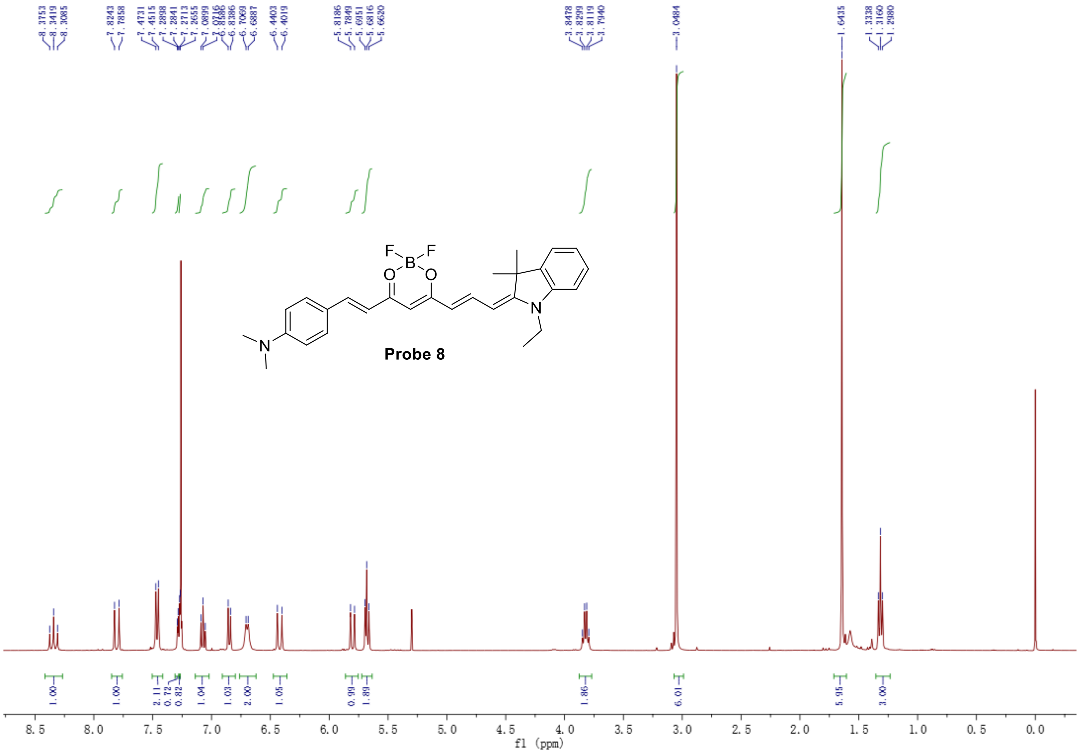

^{1}H-NMR spectra of probe **8** (400 MHz, $CDCl_3$)

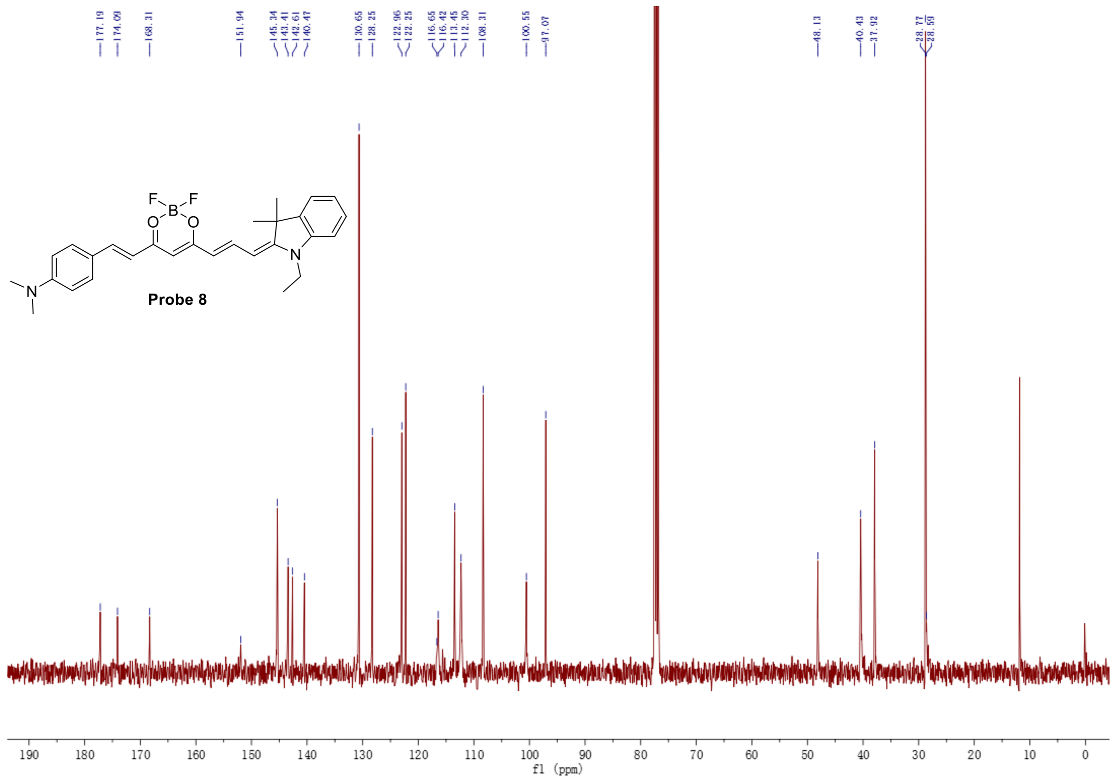

^{13}C-NMR spectra of probe **8** (101 MHz, $CDCl_3$)

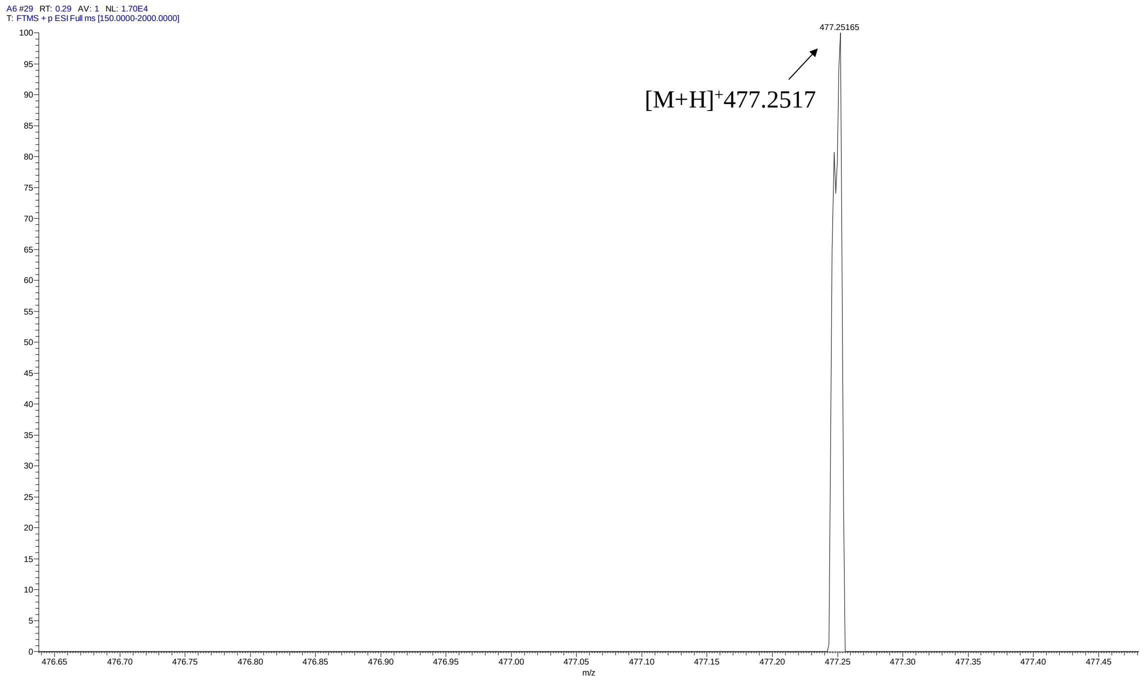

HRMS Spectrum of probe **8**

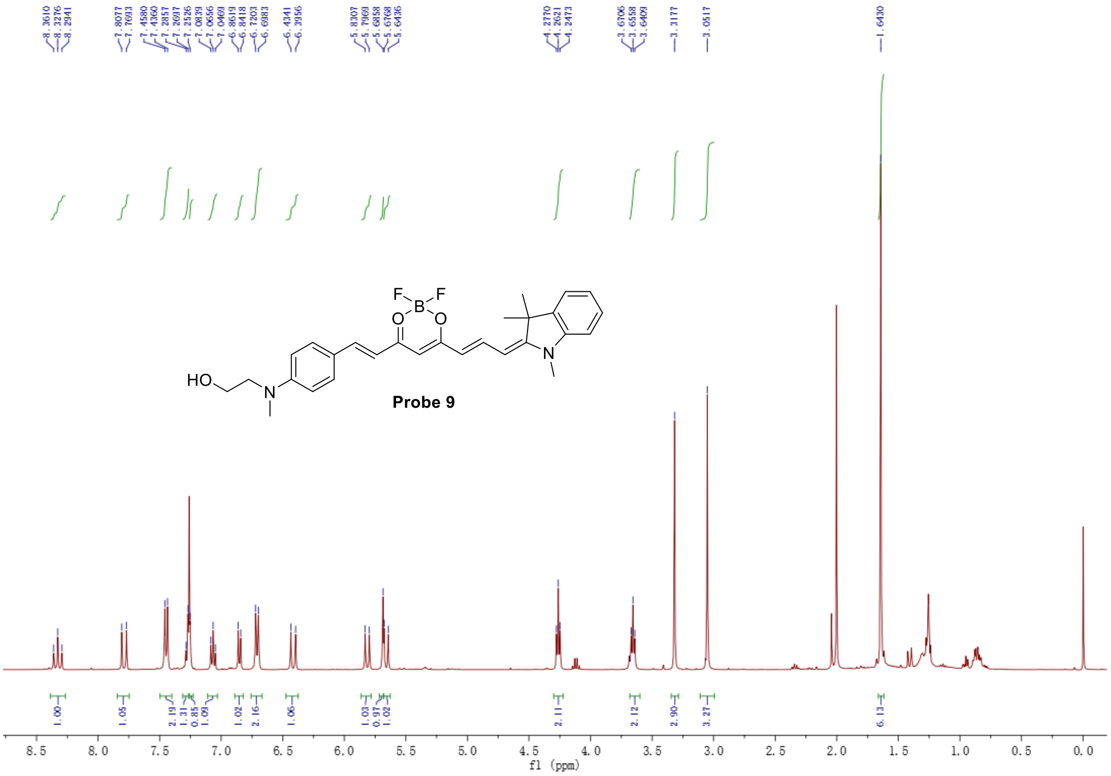

^{1}H-NMR spectra of probe **9** (400 MHz, $CDCl_3$)

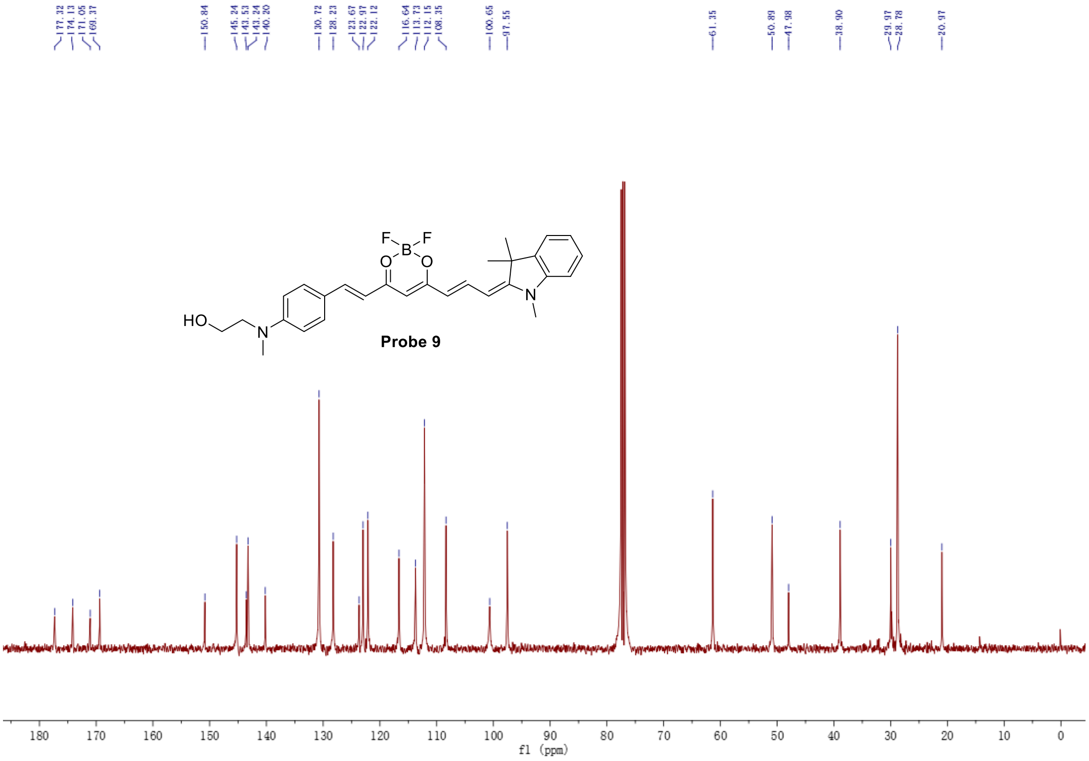

^{13}C-NMR spectra of probe **9** (101 MHz, $CDCl_3$)

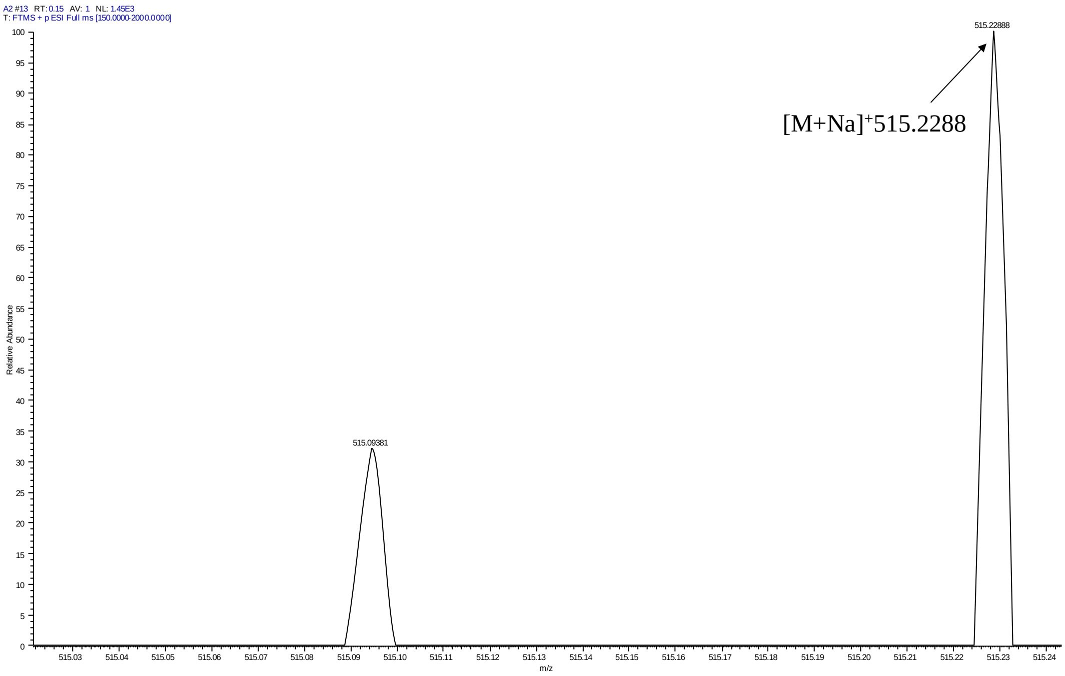

HRMS Spectrum of probe **9**
